# Systematic Comparison of Beetle Luciferase-Luciferin Pairs as Sources of Near-Infrared Light for In Vitro and In Vivo Applications

**DOI:** 10.3390/ijms23052451

**Published:** 2022-02-23

**Authors:** Bruce R. Branchini, Danielle M. Fontaine, Dawn Kohrt, Brian P. Huta, Allison R. Racela, Benjamin R. Fort, Tara L. Southworth, Aldo Roda

**Affiliations:** 1 Department of Chemistry, Connecticut College, New London, CT 06320, USA; dablamsk@conncoll.edu (D.M.F.); dkohrt@conncoll.edu (D.K.); bhuta@conncoll.edu (B.P.H.); aracela@conncoll.edu (A.R.R.); bfort@conncoll.edu (B.R.F.); tlsou@sbcglobal.net (T.L.S.); 2 Department of Chemistry “G. Ciamician”, University of Bologna, 40126 Bologna, Italy; aldo.roda@unibo.it; 3 National Institute of Biostructures and Biosystems (INBB), 00136 Rome, Italy

**Keywords:** bioluminescence, firefly, near-infrared, HEK293T, biomarker, imaging, luciferin analogs

## Abstract

Luciferases catalyze light-emitting reactions that produce a rainbow of colors from their substrates (luciferins), molecular oxygen, and often additional cofactors. These bioluminescence (BL) systems have afforded an incredible variety of basic research and medical applications. Driven by the importance of BL-based non-invasive animal imaging (BLI) applications, especially in support of cancer research, new BL systems have been developed by engineering beetle luciferase (Luc) variants and synthetic substrate combinations to produce red to near-infrared (nIR) light to improve imaging sensitivity and resolution. To stimulate the application of BLI research and advance the development of improved reagents for BLI, we undertook a systematic comparison of the spectroscopic and BL properties of seven beetle Lucs with LH_2_ and nine substrates, which included two new quinoline ring-containing analogs. The results of these experiments with purified Luc enzymes in vitro and in live HEK293T cells transfected with *luc* genes have enabled us to identify Luc/analog combinations with improved properties compared to those previously reported and to provide live cell BL data that may be relevant to in vivo imaging applications. Additionally, we found strong candidate enzyme/substrate pairs for in vitro biomarker applications requiring nIR sources with minimal visible light components. Notably, one of our new substrates paired with a previously developed Luc variant was demonstrated to be an excellent in vitro source of nIR and a potentially useful BL system for improved resolution in BLI.

## 1. Introduction

**Scheme 1:**
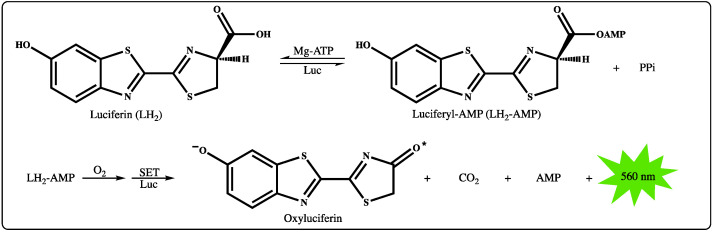
Mechanism of beetle luciferase-catalyzed bioluminescence.

Bioluminescence (BL), the emission of visible light by living organisms, is widely distributed in nature and has piqued the curiosity of humans from ancient times to the present. One commonly observed example of BL is that of the beetles including the widely studied North American firefly *Photinus pyralis*. As is the case for all characterized bioluminescent beetles, *P. pyralis* produces light from an enzyme (luciferase, Luc)-catalyzed reaction of a substrate (luciferin, LH_2_) requiring Mg-ATP and molecular oxygen. Luc-catalyzed light production (Scheme 1), which very likely proceeds through a single-electron transfer (SET) process [1,2], is an interesting example of the “substrate-assisted oxygenases” concept [3], wherein an organic substrate drives the oxidation process in the absence of cofactors. While *P. pyralis* Luc (often referred to as PpyWT and Fluc) normally produces yellow-green light (λ_max_ ~560 nm) with LH_2_, Luc mutants and various wild-type enzymes can produce emissions with maxima ranging from ~535 nm to ~630 nm [4,5,6,7]. Longer wavelength BL emission appears to be unattainable because of the limitations of the intrinsic photophysical properties of the emitter oxyluciferin (Scheme 1) [8]. 

**Figure 1:**
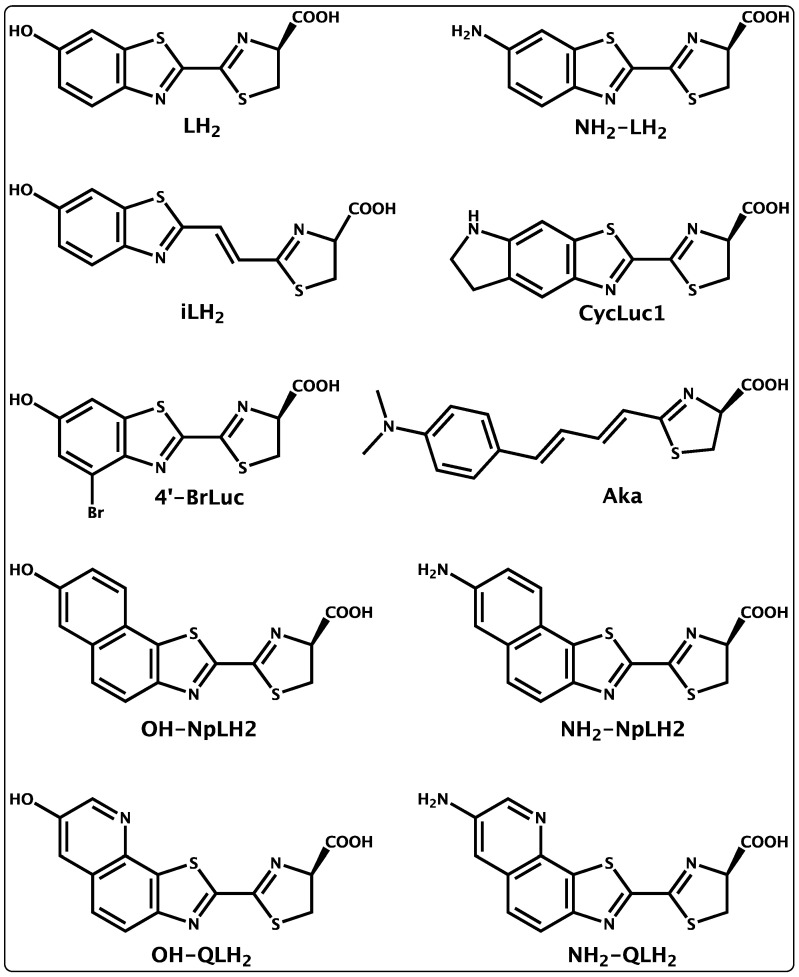
Chemical structures and abbreviations of firefly (Beetle) luciferin (LH_2_) and the substrate analogs evaluated in this study.

Paired primarily with the wild-type Luc from *P. pyralis* and several variants, a wide variety of LH_2_ analogs have been reported that extend the range of BL emission maxima from ~520 nm to ~750 nm [9,10,11,12,13,14]. Longer wavelength photon production has been accomplished without the assistance of energy transfer processes by, in most instances, extending the π conjugation of the natural substrate in several distinct structural designs [10,11,12,13,14], including the incorporation of a naphthalene ring (NH_2_-NpLH2 and OH-NpLH2) [14] (Figure 1). For substrate analogs AkaLumine-HCl (Aka) [15], infraluciferin (iLH_2_) [16,17], 4′-BrLuc [18,19], NH_2_-NpLH2 [14] and OH-NpLH2 [14], the initial BL properties determined with Fluc, a mammalian codon optimized version of *P. pyralis* Luc (Luc2), or click beetle red Luc (CBR) were optimized for noninvasive in vivo bioluminescence imaging (BLI) applications by mutagenesis strategies including directed evolution. 

The advantages of expanding BLI applications with synthetic substrate analogs and optimized orthogonal Luc/luciferin analog pairs have been discussed in highly informative recent reviews [20,21]. BLI [22], especially with methods using beetle enzymes [15], is a particularly exciting and important application of BL offering extremely low background due to the absence of inherent light emission in mammals [23,24,25]. This advantage, however, is tempered somewhat by generally weak light emission, although highly sensitive cooled CCD-based detectors mitigate this shortcoming. While the Luc2/LH_2_ system with λ_max_ ~605 nm at 37 °C has been widely used in BLI, it is limited due to absorption by hemoglobin, lipids, water and other cell components [26]. An active area of investigation to improve BLI resolution, especially for deep tissue imaging, is focused on the development of Luc/substrate pairs that emit in the near-infrared (nIR) “bio-optical” window between 650 nm and 900 nm [14,15,16,17,21,27].

A current major direction of our lab is to make highly efficient biological sources of nIR light with λ_max_ values > 700 nm (and minimal emission in the visible) for in vitro use in biosensors and biomarkers detectable with night vision technology. Previously, we made good strides towards this goal by developing an intramolecular Bioluminescence Resonance Energy Transfer (BRET) system [28] that consisted of a highly engineered PpyWT variant covalently labeled with nIR fluorescent dyes. In one example, we constructed a system that emitted with λ_max_ = 783 nm; however, ~20% of the total light emitted was at wavelengths below 700 nm due to incomplete BRET. To avoid the required, but undesirable and limiting, step of chemically modifying enzymes, we recently have focused on engineering Luc variant/luciferin analog pairs to accomplish our goal. Previously, we paired Luc2 variants with LH_2_ and a substrate analog to develop an economical and convenient dual color BL reporter gene assay to simultaneously monitor two gene expression events [29].

We report here the systematic evaluation of several purified luciferase proteins as potential in vitro sources of nIR light including: Luc2; RedFluc (Targeting Systems mammalian codon optimized red-shifted *L. italica* Luc); Akaluc (a Luc2 variant containing 28 mutations) [15]; Fluc_red (a mammalian codon optimized *P. pyralis* Luc variant containing 16 mutations) [17]; Mut51 (the Luc2 variant F243M/S347G) [30]; CBR2 (the mouse codon optimized CBR R334S/G351R variant) [14]; and PLR3 [31] (the G246A/F250H variant of the mammalian codon optimized PLR1) [29]. The seven enzymes were tested with LH_2_ and nine selected substrate analogs, including two novel quinoline ring-containing compounds that we are reporting here (Figure 1). With the exception of racemic iLH_2_, all the substrates in this study contain a 4-carboxylic acid substituted thiazoline ring in the D-configuration. 

Recognizing that enzyme/substrate pairs that produce nIR light are of great importance to the continued development and improvement of in vivo BLI methods, we focused on combinations that, with the exception of the quinoline analogs NH_2_-QLH_2_ and OH-QLH_2_, had been successfully employed in BLI studies [14,15,16,17,18,19,30,32,33,34,35,36,37,38,39]. PLR3 was matched with the quinoline-containing analogs based on the complete in vitro and live cell testing results with the seven Lucs (Table S1). While we intended to include in this study as many representative luciferase/substrate pairs as possible, a report by Viviani et al. [40] that the purified *P. hirtus* railroad worm R215K variant paired with 6′-(1-pyrrolidinyl) luciferin [10] produced bright 650 nm BL appeared after this investigation was completed. Moreover, additional interesting LH_2_ analogs have been synthesized [27,41,42] and several important additional comparative studies aimed at choosing optimal Luc/substrate pairs for in vivo BLI have appeared recently. Mezzanotte et al. compared [39] four Lucs and four substrates for this purpose, and a review by Saito-Moriya and coworkers emphasized [43] selected luciferin analogs, several of which are not included in our study. Our results have enabled us to identify strong candidate enzyme/substrate pairs for in vitro biomarker applications, to find Luc/analog combinations with improved properties compared to those previously reported, and to provide live cell BL data that may be relevant to in vivo imaging applications. 

## 2. Results and Discussion

### 2.1. Luciferin Analog Synthesis

**Scheme 2:**
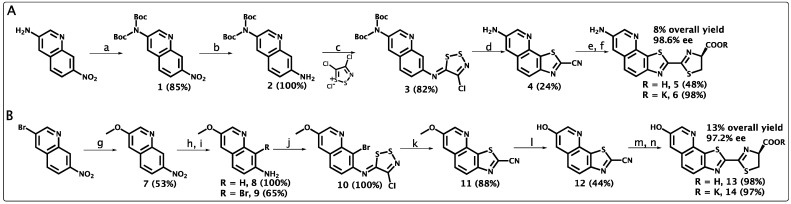
(**A**) a. THF, Boc_2_O, DMAP (cat), 50 °C, 2.5 h; b. MeOH/THF, 10% Pd/C, H_2_ 72 h; c. DCM, 1 h; d. sulfolane, microwave, 150 °C, 7 h; e. MeOH/50 mM NaHPO_4_ pH 8, D-cysteine, 3 h; f. H_2_O, KCl, KHCO_3_. (**B**) g. MeOH/toluene, Pd(OAc)_2_, Ad-BGPhos, Cs_2_CO_3_ 80 °C, 5 h; h. THF/MeOH, 10% Pd/C, H_2_, 16 h; i. CH_3_CN, NH_4_OAc (cat), NBS, −5 °C, 2.25 h; j. DCM, Appel’s salt, 4.5 h; k. pyridine, CuI, 130 ℃, 1.5 h; l. pyridine-HCl, microwave, 200 °C, 20 min; m. MeOH/50 mM NaHPO_4_ pH 8, D-cysteine, 30 min; n. H_2_O, KCl, KHCO_3_.

Based on prior results with simple quinoline- and naphthalene-substituted LH_2_ analogs [44,45], we designed the amino- and hydroxy-substituted fused quinoline ring substrates NH_2_-QLH_2_ and OH-QLH_2_, intending to similarly extend the long wavelength emission and improve the brightness of the isosteric fused naphthalene ring substrates NH_2_-NpLH2 and OH-NpLH2 [14] (Figure 1). The synthetic pathways (Scheme 2) that we developed for the preparation of the novel analogs were modeled after the approach to the corresponding naphthyl substrates [14]. The key Appel closures were accomplished using Prescher’s methodology [46] that we adapted to microwave conditions, which for NH_2_-QLH_2_ resulted in concomitant BOC group deprotection. The NH_2_-QLH_2_ compound (K^+^ salt) was prepared in six steps in 8% overall yield with 98.6% enantiomeric excess (ee) (Figure S1), while the OH-QLH_2_ (K^+^ salt) was obtained in 13% overall yield in eight steps with 97.2 % ee (Figure S2). After trials with several methods for the final condensation reaction with D-cysteine to produce the analogs, we found Miller’s procedure [47] provided highly pure products with excellent % ee and a more readily scalable approach not requiring HPLC purification. Moreover, our yields of the final condensation steps and conversion of the NH_2_-NpLH2 and OH-NpLH2 products into the corresponding potassium salts were accomplished in ~3.5-fold higher yields and with equal or greater % ee compared to the published values [14]. The syntheses of NH_2_-NpLH2, OH-NpLH2, NH_2_-QLH_2_, and OH-QLH_2_ are described in the Supplementary Materials.

### 2.2. Spectral Characterization of Substrates

**Figure 2:**
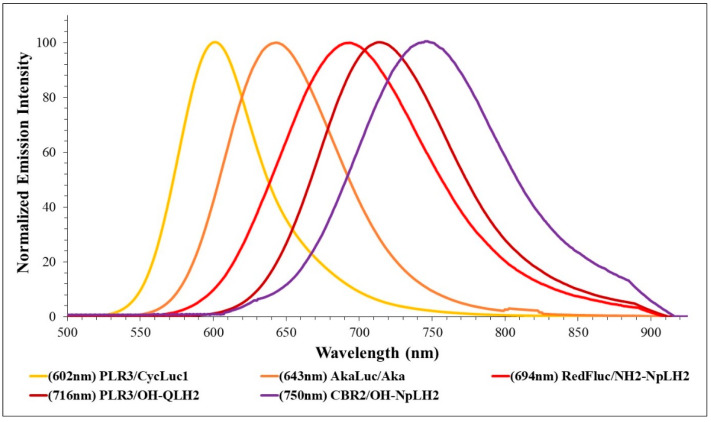
Normalized bioluminescence (BL) emission spectra of purified Luc protein/substrate pairs. BL was initiated and emission spectra were recorded at 37 °C as described in Materials and Methods, with the exception that 25 μg of protein was used for the RedFluc/NH_2_-NpLH2 measurements to enhance signal intensity.

The UV-visible (Figure S7) and fluorescence (FL) (Figure S8) spectra, and BL emission maxima (Table S1) of all Luc/substrates were measured. The BL emission spectra of representative Luc/substrate pairs that were obtained with purified Lucs at 37 °C (Figure 2) illustrate the wide range of “red” BL emission that is achievable. All substrates contain long wavelength UV peaks between 330 nm and 386 nm that somewhat correlate with the corresponding BL emission maxima. In contrast, the relationship of FL maxima to the highest BL emission maxima achieved for each substrate (Figure S9) produced an excellent linear fit (R^2^ = 0.99). While the corresponding oxyluciferins (Scheme 1) are the actual BL light emitters, the FL emission maxima of the substrates seem to have reasonably good predictive value for achievable long wavelength BL. Given that all of the substrates share the carboxy-substituted thiazoline ring and are likely to proceed through the same chemistry to form the oxyluciferin emitters, the substrate FL properties are apparently relevant. The FL quantum yields of the oxyluciferins are a key factor in determining the maximum BL efficiency, i.e., the conversion of reacted molecules of substrate into photons. While we were pleased that the FL quantum yields of the quinoline-containing substrates were ~2-fold greater than the corresponding naphthalene ones, the expected longer wavelength emission maxima were not achieved. Instead, both quinoline ring-containing substrates had ~25 nm shorter FL wavelength maxima (Figure S8). It is noteworthy that the six substrates with extended conjugation had very low FL quantum yields (0.02–0.16); whereas LH_2_ and the three analogs with equivalent conjugation had much higher values, ranging from 0.67 to 0.83 (Figure S8). 

### 2.3. Characterization of BL Properties of Luc/Substrate Analog Pairs

#### 2.3.1. Systematic Study 

A major objective of this study is to provide comparable data on the BL properties of our NH_2_-QLH_2_ and OH-QLH_2_ analogs with PLR3 and a series of Luc/substrate pairs that have been successfully applied in various in vivo BLI applications. While there is great value in applying standardized assay conditions in a systematic study, we recognize that even small changes in protocols could make it problematic to rigorously compare the results presented here to published data from other labs.

#### 2.3.2. BL Properties of Luc2 with LH_2_ and Substrate Analogs In Vitro

The results of BL testing of ten substrates with the widely used Luc2 enzyme are presented in Table 1. The in vitro measurements were made at 23 °C and BL emission spectra also were acquired at 37 °C to better estimate the emission profiles under live cell conditions. With the exception of LH_2_, whose emission shifted to ~600 nm, BL peaks remained within ± 5 nm at the higher temperature. 

Not surprisingly, the natural substrate LH_2_ is ~10 to ~3000 times brighter with Luc2 than all of the other substrates tested with this enzyme in vitro. The range of BL emission maxima for the analogs was an impressive 603 nm to 720 nm. For substrates with at least ~2% of the intensity of the Luc2/LH_2_ pair, only Aka produced light in the nIR optical window (λ_max_ = 677 nm). With the exception of 4′-BrLuc, all substrates had lower *K*_m_ values than LH_2_, and for the longest wavelength BL emitters they were 0.13 to 3.3 μM. While Luc2 can accommodate a wide array of substrate structures and can produce extraordinarily red-shifted emission (+158 nm compared to LH_2_), a great deal of brightness is lost with the longest wavelength emitters.

#### 2.3.3. BL Properties of LH_2_ with Lucs In Vitro

The results of the evaluation of the BL properties of the seven Lucs with the natural LH_2_ substrate are included in Table 2. The BL spectral data confirm that long wavelength emission beyond ~625 nm is highly unlikely to be achievable with the natural substrate.

#### 2.3.4. Enhanced In Vitro nIR Sources for Biosensor and Biomarker Applications

**Figure 3:**
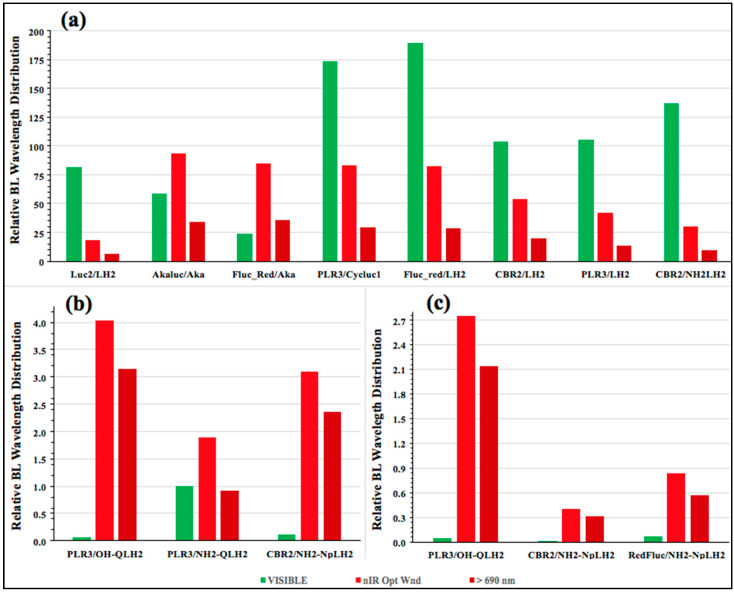
Relative (to Luc2/LH_2_) BL emission wavelength distribution of selected Luc/substrate pairs. BL reactions were monitored through filters and the relative percentages of visible, nIR window, and nIR emissions (≥690 nm) were determined as described in Materials and Methods. Live cell results. (**a**,**b**) Wavelength distributions were corrected for the live cell specific activities. In vitro results. (**c**) Wavelength distributions were corrected for the purified Luc specific activities. Intensities of visible (500–640 nm, green), nIR window (650–810 nm, red), and nIR (690–810 nm, maroon) light are shown.

Focusing on our goal to make highly efficient biological sources of nIR light with λ_max_ values > 690 nm and with minimal emission in the visible for use as biosensors and biomarkers, we measured the % BL emission > 690 nm of the Luc/substrates and adjusted the data for their relative in vitro specific activities. The top three enzyme/substrate pairs (Figure 3c) had BL λ_max_, % visible, and % > 690 nm emission values of: PLR3/OH-QLH_2_ (718 nm, 2%, 76%); CBR2/NH_2_-NpLH2 (721 nm, 3%, 73%); and Red_Fluc/NH_2_-NpLH2 (694 nm, 8%, 62%). PLR3/OH-QLH_2_ and CBR2/NH_2_-NpLH2 are excellent sources of nIR and their greater BL maxima are clearly the major contributing factor. While the intensities of these sources are low, for in vitro applications it should be possible to significantly improve the sensitivity mainly by increasing the concentrations of the enzymes and maintaining sufficient excess of the substrates.

#### 2.3.5. BL Properties of Luc2 with LH_2_ and Substrate Analogs in Live Cells

We obtained live HEK293T cell BL activity data for all of the Lucs (Table 1, Table 2 and Table 3 and Table S1), and normalized them to an internal transfection efficiency control (Nluc activity). The data are reported relative to the Luc2/LH_2_ pair with an underlying assumption that the expression levels of the other Lucs are very similar to that of Luc2. In the live cell experiments, Luc2 was expressed and assayed at 37 °C. The live cell BL emission in all experiments was consistently greatest when measured through the bandpass filter that corresponded to the 37 °C BL emission maximum measured with purified proteins (Section 3 and Table S1). Compared to the in vitro Luc protein results (Table 1) in which saturating concentrations of substrates were used, there are additional determinants of the intensity of the BL signals in live cells including the permeability of substrates across the cell membrane and the apparent affinity of the Lucs for the substrates (*K*_m_ in the cell environment). By normalizing the specific activity results to the purified Luc2 protein/LH_2_ pair and obtaining the live cell activities of the substrates with Luc2 (essentially eliminating protein expression as a variable), we could reasonably compare the cellular properties of the substrates (Table 1). The live cell specific activities followed the same general trend as the in vitro values; however, the ~4- to ~5-fold greater live cell values with CycLuc1, Aka, and NH_2_-LH_2_ were impressive. Interestingly, the *K*_m_ values of these substrates were ~3–25-fold lower than that of LH_2_. For these substrates, the *K*_m_ values may be correlated to cell permeability and, with the exception of NH_2_-LH_2_, the substrates have higher LogP values (Table S2) that are consistent with cell membrane permeability being an important determinant of specific activity. Notably, analogs containing amino group substituents had relative activities 2.2–5.7-fold higher in live cells. In particular, the membrane permeability of NH_2_-NpLH2 and NH_2_-QLH_2_ was greater than that of LH_2_ and OH-QLH_2_. (Figure S10). 

#### 2.3.6. BL Properties of LH_2_ with Lucs in Live Cells

A comparison of the relative in vitro and live cell specific activities revealed that greater (than Luc2) values were obtained in HEK293T cells for PLR3 (1.5-fold), CBR2 (1.6-fold) and Fluc_red (2.7-fold). Interestingly, these results suggest that enhanced sensitivity over the widely used Luc2/LH_2_ pair may be achieved in BLI with PLR3, as they have with CBR2 [14,39] and Fluc_red [17]. It is likely that the favorable properties of these enzymes reflect enzyme stability at 37 °C, which is greater than that of Luc2.

#### 2.3.7. BL Properties of Optimized and Novel Luc/Substrate Combinations in Live Cells

With the exception of our recently developed NH_2_-QLH_2_ and OH-QLH_2_, the substrates in this study have been widely employed in BLI applications. We determined the BL properties of these optimized enzyme/substrate pairs along with unreported combinations that, in some cases, exceeded them (Table 3). In PC3M cells expressing *P. pyralis* Luc, a ratio of ~5.5 was reported for LH_2_/NH_2_-LH_2_ [32]. Our results with Luc2 in live cells were similar (LH_2_/NH_2_-LH_2_: ratio = 2.8), while CBR2 produced the greatest signal strength with NH_2_-LH_2_ (Table 3); a result also observed with the F247L variant of Fluc [48]. The commercial availability of this substrate makes it an interesting possibility for BLI applications. Additionally, NH_2_-LH_2_ has been generated intracellularly from stable precursors in a BLI study with transfected MDA-MB-231 tumor cells [35]. 

We benchmarked our evaluation of CycLuc1 paired with Luc2 (Table 1). While pairing this cyclic amino group-containing substrate with PLR3 produced a 1.6-fold increase in in vitro activity, a more significant 5.5-fold enhancement was realized in HEK293T cells (Table 3). In other mammalian cells, an 18-fold improvement in BL has been reported for a *P. pyralis* Luc S347A mutant [48]. Interestingly, CycLuc1 has excellent bioavailability in mice in vivo, based on its outstanding performance compared to LH_2_ in BLI brain and deep tissue imaging studies [49]. The prospects of further improving BLI with this substrate by pairing it with PLR3 are exciting. Similarly, RedFluc displayed greater (1.8-fold) activity in live cells using Cycluc1 than with LH_2_, albeit with modestly blue-shifted emission (Table S1).

Among the longest wavelength BL systems, the optimized Fluc_red/iLH_2_ pair produced modest BL intensity at 707 nm (Table 3), yet has provided excellent sensitivity in a dual BLI format when paired with a green light emitting Fluc mutant and LH_2_ [17]. Even greater sensitivity and longer wavelength nIR BLI may be possible with CBR2/iLH_2_ as it increased live cell BL 2.3-fold, with maximum emission increased by 20 nm (Table 3) compared to Fluc_red/iLH2. 

The Akaluc enzyme contains 28 amino acid changes introduced through directed evolution [15]. Paired with Aka in HEK293T cells, we found an Akaluc/Luc2 ratio of ~15 in BL activity. While the activity of this optimized enzyme/substrate pair is poor in vitro, its live cell activity with 643 nm emission is the highest among all the long wavelength systems we measured (Table 3). The remarkable BLI imaging of single cells that has been realized with the Akaluc/Aka pair is a reminder of the limitations of using only in vitro assays under ideal conditions to predict strong BLI candidates. 

The nIR-emitting combination CBR2/NH_2_-NpLH2 has been successfully employed in BLI applications [14,39] despite its unimpressive activity using purified enzyme. Only PLR3 and Fluc_red produced good BL with this substrate in live cells, but at the expense of ~30 nm blue shifts (Table S1). The activity of the OH-NpLH2 substrate paired with CBR2 was 1.4-fold lower than that of CBR2/NH_2_-NpLH2 in live cells (Table 3). However, the emission maximum obtained with OH-NpLH2 was an impressive 750 nm.

NH_2_-QLH_2_ and OH-QLH_2_ paired optimally and very effectively with PLR3. Interestingly, as with the isosteric naphthyl-containing substrates, the hydroxyl substituent produced longer wavelength nIR (λ_max_ = 716 nm) BLI; however, with OH-QLH_2_, the BL intensity was also greater. Moreover, the PLR3/OH-QLH_2_ pair produced live cell BL ~1.3-fold greater than the CBR2/NH_2_-NpLH2 pair with similar nIR emission (λ_max_ = 718 nm) (Table 3). These encouraging results (Figure S11) will be pursued in future BLI studies. With CBR2, wild-type CBR was improved by the introduction of the amino acid changes R337S and G354R (Luc2 numbering) [14]. Introduction of these changes into PLR3 did not improve its BL properties. Based on sequence comparisons and the testing of OH-QLH_2_, with several previously published Lucs [5,31], it appears that the mutations G246A and F250H in PLR3 are major contributors to enabling the considerably larger quinoline ring-containing substrates to be accommodated productively at the enzyme’s active site. We had shown previously that these residues which occupy the first turn of an active site defining helix [50], can form side chain to main chain H-bonds [50]. Perhaps this type of interaction allows local conformation changes that enables productive binding of the larger aromatic ring systems.

Mut51 was discovered in the Prescher lab using a novel parallel screening strategy [30]. In BLI of mice expressing the Mut51 variant in DB7 cells, 4′-BrLuc and LH_2_ were shown to be an excellent orthogonal pair with 4′-BrLuc providing a sufficiently strong signal and LH_2_ an essentially undetectable one [30]. We found the specific activity ratio (4′-BrLuc/LH_2_) with purified Mut51 was ~1 and in HEK293T cells the ratio was ~2 (Table 2 and Table 3). Unfortunately, our methods were unable to predict the selectivity of Mut51 observed in the BLI study. While differences in light measuring methodology and/or BL in the different cell lines is important, it is also possible that the bioavailability of 4′-BrLuc in mice is an important determinant. Notably, the LogP and RP-HPLC retention times (Table S2) strongly suggest that the halogen-containing substrate is much more hydrophobic than LH_2_. In addition, while pairing PLR3 with 4′-BrLuc may not produce a useful orthogonal pair with LH_2_, PLR3 produces 23- and 32-fold greater BL in vitro and in HEK293T cells, respectively, than does Mut51 (Table 3). 

#### 2.3.8. Enhanced nIR Sources for BLI Applications

We addressed the question of improving the resolution of the widely employed Luc2/LH_2_ combination in BLI that would require increased photon output and/or greater emission in the nIR optical window. Using experiments in live HEK293T cells as a model, we measured the percentage of visible and nIR emission of the Luc/substrate pairs and adjusted the data for their relative specific activities (Figure 3a). Seven of the enzyme/substrate combinations had relative nIR window output that exceeded that of Luc2/LH_2_. Excellent BLI results have been reported for Akaluc/Aka [15], CBR2/LH_2_ [14,39] and Fluc_red/LH_2_ [17]. Our model data show that the 4.4-fold Fluc_red/LH_2_ improvement is almost entirely due to its higher specific activity; whereas Akaluc/Aka (5.0-fold) and CBR2/LH_2_ (2.9-fold) have greater specific activity and considerably longer wavelength emission. The live cell model data indicated that the previously unreported combinations Fluc_red/Aka (4.6-fold), CBR2/NH_2_-LH_2_ (1.6-fold), PLR3/LH_2_ (2.3-fold), and PLR3/CycLuc1 (4.5-fold) are potential candidates to achieve high resolution BLI. Extraordinarily high percentages of nIR optical window emission were observed for CBR2/NH_2_-NpLH2 (97%) and PLR3/OH-QLH_2_ (98%) (Figure 3b). The former pair has successfully provided higher resolution in deeper tissue BLI images [14,39] that may also be realized with PLR3/OH-QLH_2_.

## 3. Materials and Methods

### 3.1. Materials

The following materials were obtained from the sources indicated: Mg-ATP (bacterial source) and ATP (disodium salt hydrate) from Sigma-Aldrich (St. Louis, MO, USA); Glutathione Sepharose 4B media and pGEX-6P-2 vector from GE Healthcare (Piscataway, NJ, USA); Ni-NTA Agarose resin (Valencia, CA, USA); the basic pNL3.1 [*Nluc*/minP] vector, containing the mammalian codon optimized *Nluc* gene, which is under the control of a minimal promotor and contains a multiple cloning region for insertion of a promotor and/or gene of choice (Promega, Madison, WI, USA); HEK293T cells were donated by Martha Grossel (Connecticut College, New London, CT, USA). Plasmids containing *luc* genes for the following were obtained as generous gifts: Luc2 in the pF4Ag vector from Lance Encell (Promega, Madison, WI, USA); CBR2opt (CBR2) (ATG-1929) [14] in the pF4Ag vector from Keith Wood (Addgene plasmid # 108712; http://n2t.net/addgene:108712 accessed on 8 May 2019; RRID:Addgene_108712); Mutant51 (Mut51) in the pET28a His-tag vector from Jennifer A. Prescher (University of California, Irvine, CA, USA); Fluc_red gene in the SFG vector from Cassandra L. Stowe (University College London, London, UK); and RedFluc in the pCMV vector from Rampyari Walia (Targeting Systems, El Cajon, CA, USA). Akaluc in the pCDH-EF1-MCS-T2A-copGFP vector was obtained from Gene Dynamics, LLC (Portland, OR, USA), and PLR3 in the pF4Ag vector was described previously [31]. 

The following Luc proteins were expressed in *Escherichia coli* strain BL21(DE3) pLysS from the pGex-6P-2 vector: Luc2, Akaluc, CBR2, RedFluc, and PLR3. The Luc2, Akaluc and CBR2 genes were subcloned from their respective vectors. The RedFluc gene previously described in US Patent number 7,807,429 B2; LitS-S-11/F467R was used directly and the PLR3 gene was constructed by inserting the G246A and F250H mutations into the PLR1 gene [29]. The Fluc_red gene was constructed by introducing the S284T, H354R, and A357Y mutations into the x11a Fluc gene in the pET16b His-tag vector (Amit Jathoul, Cardiff University, Cardiff, Wales, UK), and the Mut51 gene in the pET28a His-tag vector was used as received. 

The HEK293T cell studies were performed using mammalian codon optimized *luc* genes under the CMV promoter in the pNL3.1 [*Nluc*/minP] Vector. The genes encoding Luc2, CBR2, PLR3, and Mut51 were subcloned from the pF4Ag vector by ligation into the multiple cloning region of the pNL3.1 vector using the XhoI and EcoRV restriction sites. The Fluc_red, Akaluc and RedFluc genes were subcloned by replacing the Luc2 gene in the CMV-Luc2-pNL3.1 vector using the AsiSI and EcoRV restriction sites. 

The structures of the substrates used in this study are shown in Figure 1. ((*S*)-2-((1*E*,3*E*)-4-(4-(dimethylamino)phenyl)buta-1,3-dien-1-yl)-4,5-dihydrothiazole-4-carboxylic acid) was purchased from Sigma Aldrich (St. Louis, MO, USA) as TokeOni and is also known as AkaLumine-HCl (Aka). The following substrates were generous gifts: potassium salts of Beetle LH_2_ ((*S*)-2-(6-hydroxybenzo[*d*]thiazol-2-yl)-4,5-dihydrothiazole-4-carboxylic acid) and (*S*)-2-(6-amino benzo[*d*]thiazol-2-yl)-4,5-dihydrothiazole-4-carboxylic acid (NH_2_-LH_2_) from Promega (Madison, WI, USA); ((*S*,*E*)-2-(2-(6-hydroxybenzo[*d*]thiazol-2-yl)vinyl)-4,5-dihydrothiazole-4-carboxylic acid) (iLH_2_) from James C. Anderson (University College, London, UK); ((*S*)-2-(6,7-dihydro-5*H*-thiazolo[4,5-*f*]indol-2-yl)-4,5-dihydrothiazole-4-carboxylic acid) (CycLuc1) from Stephen C. Miller (University of Massachusetts-Amherst, Amherst, MA, USA); and ((*S*)-2-(4-bromo-6-hydroxybenzo[*d*]thiazol-2-yl)-4,5-dihydrothiazole-4-carboxylic acid) (4′-BrLuc) from Jennifer A. Prescher (University of California, Irvine, CA, USA). The syntheses of NH_2_-NpLH2, OH-NpLH2, NH_2_-QLH_2_, and OH-QLH_2_ are described in the Supplementary Materials.

### 3.2. General Methods

All *luc* gene sequences were verified by DNA sequencing at the W. M. Keck Biotechnology Laboratory (Yale University, New Haven, CT, USA). The Luc2, Akaluc, RedFluc, CBR2 and PLR3 genes were expressed in the pGEX-6P-2 expression vector and the purified enzymes contain the N-terminal peptide extension GlyProLeuGlySer-. The Mut51 and Fluc_red genes were expressed in His-tag expression vectors pET28a and pET16b, respectively. Concentrations of purified proteins were determined using a NanoDrop™ Lite Spectrophotometer. Detailed protocols for protein expression and purification and K*_m_* measurements are found in the Supplementary Methods.

### 3.3. Bioluminescence Emission Spectra

BL was initiated by mixing equal volumes (0.25 mL) of a solution of 50 mM Tricine pH 7.4 containing 2 mM ATP and 6 mM MgSO_4_ with a solution of assay buffer containing 12.5 μg of enzyme and 0.1 mM of the indicated analog in a quartz cuvette. All solutions were pre-warmed to either 23 °C or 37 °C. The final concentrations of the mixture (0.5 mL) in 50 mM Tricine pH 7.4 were 0.4 μM enzyme, 50 μM of the indicated analog, 1 mM ATP, and 3 mM MgSO_4_. Emission spectra were acquired at 23 °C and 37 °C after a 1 min delay with a Horiba Jobin-Yvon iHR 320 imaging spectrometer equipped with a liquid N_2_ cooled CCD detector. Data were collected over the wavelength range 450–925 nm, with the excitation source turned off and the emission slit width set to 10 nm, and were corrected for the spectral response of the detector using a correction curve provided by the manufacturer. The pH values were confirmed before and after spectra were obtained (Figure 2).

### 3.4. In Vitro Specific Activities

All assays were performed in triplicate in white 96-well microtiter plates containing 2.5 μg of purified enzyme and 50 μL of 0.1 mM analog in 50 mM Tricine pH 7.4. BL was initiated by the automated injection of 50 μL of 50 mM Tricine pH 7.4 containing 2 mM ATP, and 6 mM MgSO_4_. Signals were monitored over 2 min using a Synergy™ 2 microplate luminometer (BioTek, Winooski, VT, USA). Data were integrated and corrected for the spectral response of the Hamamatsu R928 PMT detector.

### 3.5. In Vitro Bioluminescence Wavelength Emission Distribution

Assays were performed in 96-well black clear bottom plates containing 0.5 μg purified Lucs (except that 0.1 μg of Luc2 was used with LH_2_). Assay mix (0.1 mL) containing 0.1 mM analog, 0.1 mM Na-ATP, 1 mM MgSO_4_ in 50 mM Tricine, pH 7.4 was then added to each well. After a 45 s incubation to ensure that emission decay was minimal, BL was measured with an IVIS Spectrum III (Perkin Elmer, Waltham, MA, USA) using the auto setting, FOV B, and filters set at OPEN, 520 ± 20 nm, 570 ± 20 nm, 620 ± 20 nm, 670 ± 20 nm, 710 ± 20 nm, 755 ± 15 nm and 790 ± 20 nm. Data were analyzed with the Living Image 4.7 Software (Perkin Elmer, Waltham, MA, USA) by selecting the appropriate region of interest (ROI) and were reported as radiance (p/s/cm^2^/sr). The radiance values were corrected for any emission decay that occurred as the filters were sequentially imaged. Experiments were performed in triplicate and individual trials were repeated at least three times. The intensities used to calculate the values presented in Figure 3 are based on the sum of the data through the following filters: visible (520 nm, 570 nm, 620 nm, and 670 nm); nIR optical window (670 nm, 710 nm, 755 nm, and 790 nm); and nIR (710nm, 755 nm, and 790 nm).

### 3.6. Cell Culture and Transfection

HEK293T cells were grown in Dulbecco’s modified Eagles medium supplemented with 10% fetal bovine serum. Cells were counted using a TC10 automated cell counter (BioRad, Hercules, CA, USA) and plated at 1,250,000 cells per well in a 6-well plate and grown at 37 °C with 5% CO_2_ for 4–6 h prior to transfection.

One microgram of *luc-pNL3.1* plasmid DNA plus 1 μg pF4Ag empty vector in 0.125 mL OptiMEM (Invitrogen/Thermofisher, Waltham, MA, USA) was added to 0.125 mL OptiMEM containing 6 μL Lipofectamine 2000 transfection reagent (Invitrogen/Thermofisher, Waltham, MA, USA). The mixtures were incubated for 10 min at room temp and were added to the prepared HEK293T cells in 6-well plates.

### 3.7. Live Cell Imaging in Transfected HEK293T Cells

Transfected cells were grown for 20 h at 37 °C with 5% CO_2_ and were released from the plate with 0.05% trypsin-EDTA (Gibco, Waltham, MA, USA), resuspended in growth media, and counted. Each well of a black clear-bottom 96-well plate was seeded with 50,000 cells in 0.1 mL growth media and grown for an additional 24 h. For all series of substrates examined by each Luc, a separate set of quadruplicate wells was seeded for Nluc activity analysis using the Nano Glo Luciferase Assay Kit (Promega, Madison WI, USA). Growth media was removed and replaced with 0.05 mL DMEM without phenol red plus 10% FBS. Nluc assay buffer mix (0.05 mL) was added, mixed by pipetting and BL was measured after a 3 min incubation at room temp. BL was measured using an IVIS Spectrum III (Perkin Elmer) with the auto exposure, FOV B, and OPEN filter settings. For the Luc-substrate assays, growth media was removed and 0.1 mL of 0.5 mM solutions of LH_2_ or analogs in assay buffer (50 mM Tricine (pH 7.4), growth media (1:1, *v*/*v*), and 5 μM ATP) were added to each well. After a 30 s incubation, BL was measure at 37 °C. Signals were monitored for 8 min with a measurement at 30 s intervals. Data were analyzed with the Living Image 4.7 Software (Perkin Elmer) after selecting the appropriate region of interest (ROI). The highest average Luc–substrate activity recorded within the first 5 min was used to calculate the final average radiance (p/s/cm^2^/sr). In order to account for daily variations in transfection efficiencies, the reported radiance values were calculated from the mean ± standard deviation of BL signals corrected by the respective Nluc activities. Each experiment was repeated at least 3 times.

## 4. Conclusions

We have provided a systematic comparison of the spectroscopic and BL properties of seven beetle Lucs with LH_2_ and nine substrate analogs including NH_2_-QLH_2_ and OH-QLH_2_ that were disclosed here. The major general findings include: (1) the excellent correlation of FL maxima with the maximum achievable BL emission wavelengths; (2) the reasonable effectiveness of live HEK293T testing, but less reliable predictive ability of in vitro purified Luc specific activity data, in predicting BLI performance; (3) the incredible ~160 nm range of red-shifted BL emission maxima catalyzed by Luc2; (4) further documentation of the ~625 nm limitation of BL with the natural LH_2_ substrate; and (5) the unfortunate correlation of weak FL quantum yields with poor BL brightness among systems that emit at the longest nIR wavelengths. Specific significant findings included: (1) identifying Aka as the best substrate that combines brightness and long wavelength BL with Luc2 in live cells; (2) demonstrating that CBR2 and PLR3 produce brighter and significantly longer wavelength BL with LH_2_ than Luc2 in live cells; (3) finding that iLH_2_ produced brighter and longer wavelength nIR with CBR2 than with Fluc_red; (4) showing that the Akaluc/Aka pair emitted the brightest long wavelength (>640 nm) BL in HEK293T cells; (5) determining that PLR3 should be advantageous in improving brightness with substrates CycLuc1 and 4′-BrLuc; and (6) identifying the CBR2/NH_2_-NpLH2 and PLR3/OH-QLH_2_ combinations as the best pairs for high resolution nIR BL in live cells (Figure S11) and for in vitro applications requiring “pure” nIR sources. While the PLR3/OH-QLH_2_ combination is a successfully engineered in vitro biological source of nIR light, it is likely that further optimization of PLR3 by directed evolution will be necessary for superior BLI applications.

## Figures and Tables

**Table 1:** BL emission and relative activities of substrates with Luc2.

Substrate	Specific Activity ^1^	BL, 23 °C (λ_max_ ± 2 nm) ^2^	BL, 37 °C (λ_max_ ± 2 nm) ^2^	Live Cell Activity ^3^	*K*_m_ (μM) ^4^
LH_2_	100 ± 4	562 (84)	600, 574 (105)	100 ± 9.9	15 ± 2
NH_2_-LH_2_	9.1 ± 0.48	603 (85)	602 (80)	36 ± 3.9	5 ± 1.2
CycLuc1	8.3 ± 1.9	609 (61)	606 (64)	47 ± 4.3	0.61 ± 0.2
Aka	1.9 ± 0.01	677 (90)	674 (88)	10 ± 1.0	2.2 ± 0.2
iLH_2_	0.07 ± 0.001	709 (96)	708 (99)	0.06 ± 0.007	0.13 ± 0.05
4′-BrLuc	8.3 ± 0.78	617 (70)	618 (70)	5.7 ± 1.7	44 ± 11
NH_2_-NpLH2	0.18 ± 0.003	708 (135)	706 (126)	0.58 ± 0.06	3.3 ± 0.6
OH-NpLH2	b.d.	b.d.	b.d.	b.d.	b.d.
NH_2_-QLH_2_	0.08 ± 0.001	678 (139)	678 (130)	0.18 ± 0.01	1.5 ± 0.2
OH-QLH_2_	0.03 ± 0.003	720 (154)	719 (133)	0.03 ± 0.003	1.8 ± 0.6

^1^ Specific activities were obtained from assays at pH 7.4 and 23 °C by monitoring and integrating signal intensity for 2 min using purified enzyme (2.5 µg), 50 µM substrate, 1 mM ATP, and 3 mM MgSO_4_. Data are expressed as the mean ± standard deviation and are reported relative to the Luc2/LH_2_ value, defined as 100 and were corrected for the spectral response of the CCD detector. ^2^ Data determined from BL emission spectra measured in in vitro assays were obtained as described in Materials and Methods. Bandwidths at full width at half-maximum values are given in parentheses. ^3^ Average radiance [p/s/cm^2^/sr] of BL measured from *luc2* transfected HEK293T cells with the open filter setting of an IVIS Spectrum III instrument. BL is measured at the highest level attained within 5 min of 0.5 mM substrate addition. Data are expressed as the mean ± standard deviation and are reported relative to the Luc2/LH_2_ value, as described in Materials and Methods. b.d.; below detection having a relative activity of <0.03. ^4^ V_0_ values used to determine the constants were obtained from peak-height measurements as described in detail in the Supplementary Materials.

**Table 2:** BL emission maxima and relative activities of seven luciferases with LH_2_.

Luc/LH_2_	Specific Activity ^1^	BL, 23 °C (λ_max_ ± 2 nm) ^2^	BL, 37 °C (λ_max_ ± 2 nm) ^2^	Live Cell Activity ^3^	*K*_m_ (μM) ^4^
Luc2	100 ± 4	562 (84)	600, 574 (105)	100 ± 9.9	15 ± 2
RedFluc	51 ± 1.5	612 (60)	613 (63)	27 ± 2.5	66 ± 7
Akaluc	0.1 ± 0.01	598 (73)	597 (73)	4.2 ± 0.6	64 ± 10
Fluc_red	47 ± 2.6	602 (76)	604 (68)	272 ± 27	11 ± 0.9
Mut51	1.9 ± 0.02	568 (101)	612 (86)	0.9 ± 0.03	98 ± 7
CBR2	52 ± 0.26	621 (65)	619 (64)	156 ± 15	250 ± 24
PLR3	74 ± 8	613 (61)	611 (62)	149 ± 17	15 ± 4

Footnotes ^1–4^ can be found in Table 1.

**Table 3:** BL emission maxima and relative activities of optimized and intriguing Luc/substrate pairs.

Luc/Substrate	Specific Activity ^1^	BL, 23 °C (λ_max_ ± 2 nm) ^2^	BL, 37 °C (λ_max_ ± 2 nm) ^2^	Live Cell Activity ^3^	*K*_m_ (μM) ^4^
Luc2/LH_2_	100 ± 4	562 (84)	600, 574 (105)	100 ± 9.9	15 ± 2
CBR2/NH_2_-LH_2_	10 ± 0.24	598 (71)	600 (72)	168 ± 30	9.5 ± 1.3
PLR3/CycLuc1	13.2 ± 0.24	606 (62)	602 (65)	257 ± 13	0.45 ± 0.1
Fluc_red/iLH_2_	0.14 ± 0.02	707 (93)	707 (86)	0.56 ± 0.03	0.17 ± 0.05
CBR2/iLH_2_	0.14 ± 0.002	730 (89)	727 (87)	1.3 ± 0.04	0.9 ± 0.08
Akaluc/Aka	1.6 ± 0.03	642 (93)	643 (89)	152 ± 18	0.3 ± 0.05
CBR2/NH_2_-NpLH2	0.42 ± 0.01	721 (120)	718 (117)	3.2 ± 0.09	0.8 ± 0.1
CBR2/OH-NpLH2	0.48 ± 0.01	750 (117)	749 (116)	2.2 ± 0.13	0.93 ± 0.13
PLR3/NH_2_-QLH_2_	1.23 ± 0.003	649 (111)	649 (113)	2.9 ± 0.14	0.7 ± 0.15
PLR3/OH-QLH_2_	2.8 ± 0.06	718 (107)	716 (107)	4.1 ± 0.35	1.6 ± 0.1
Mut51/4′-BrLuc	1.9 ± 0.08	617 (77)	618 (76)	2.1 ± 0.34	2.3 ± 0.2
PLR3/4′-BrLuc	44 ± 2	615 (63)	615 (69)	68 ± 5.8	4.5 ± 0.4

Footnotes ^1–4^ can be found in Table 1.

## Data Availability

Not applicable.

## Supplementary Materials

The following supporting information can be downloaded at: https://www.mdpi.com/article/10.3390/ijms23052451/s1.

## References

[1] Mofford D.M., Reddy G.R., Miller S.C. (2014). Latent luciferase activity in the fruit fly revealed by a synthetic luciferin. Proc. Natl. Acad. Sci. USA.

[2] Branchini B.R., Behney C.E., Southworth T.L., Fontaine D.M., Gulick A.M., Vinyard D.J., Brudvig G.W. (2015). Experimental Support for a Single Electron-Transfer Oxidation Mechanism in Firefly Bioluminescence. J. Am. Chem. Soc..

[3] Fetzner S., Steiner R.A. (2010). Cofactor-independent oxidases and oxygenases. Appl. Microbiol. Biotechnol..

[4] Viviani V.R. (2002). The origin, diversity, and structure function relationships of insect luciferases. Cell. Mol. Life Sci..

[5] Branchini B.R., Ablamsky D.M., Davis A.L., Southworth T.L., Butler B., Fan F., Jathoul A.P., Pule M.A. (2010). Red-emitting luciferases for bioluminescence reporter and imaging applications. Anal. Biochem..

[6] Viviani V.R., Amaral D., Prado R., Arnoldi F.G.C. (2011). A new blue-shifted luciferase from the Brazilian Amydetes fanestratus (Coleoptera: Lampyridae) firefly: Molecular evolution and structural/functional properties. Photochem. Photobiol. Sci..

[7] Nishiguchi T., Yamada T., Nasu Y., Ito M., Yoshimura H., Ozawa T. (2015). Development of red-shifted mutants derived from luciferase of Brazilian click beetle Pyrearinus termitilluminans. J. Biomed. Opt..

[8] Naumov P., Ozawa Y., Ohkubo K., Fukuzumi S. (2009). Structure and Spectroscopy of Oxyluciferin, the Light Emitter of the Firefly Bioluminescence. J. Am. Chem. Soc..

[9] Woodroofe C.C., Meisenheimer P.L., Klaubert D.H., Kovic Y., Rosenberg J.C., Behney C.E., Southworth T.L., Branchini B.R. (2012). Novel Heterocyclic Analogues of Firefly Luciferin. Biochemistry.

[10] Iwano S., Obata R., Miura C., Kiyama M., Hama K., Nakamura M., Amano Y., Kojima S., Hirano T., Maki S. (2013). Development of simple firefly luciferin analogs emitting blue, green, red, and near-infrared biological window light. Tetrahedron.

[11] Miura C., Kiyama M., Iwano S., Ito K., Obata R., Hirano T., Maki S., Niwa H. (2013). Synthesis and luminescence properties of biphenyl-type firefly luciferin analogs with a new, near-infrared light-emitting bioluminophore. Tetrahedron.

[12] Jathoul A.P., Grounds H., Anderson J.C., Pule M.A. (2014). A Dual-Color Far-Red to Near-Infrared Firefly Luciferin Analogue Designed for Multiparametric Bioluminescence Imaging. Angew. Chem. Int. Ed..

[13] Anderson J.C., Grounds H., Jathoul A.P., Murray J.A.H., Pacman S.J., Tisi L. (2017). Convergent synthesis and optical properties of near-infrared emitting bioluminescent infra-luciferins. Rsc Adv..

[14] Hall M.P., Woodroofe C.C., Wood M.G., Que I., Van’T Root M., Ridwan Y., Shi C., Kirkland T.A., Encell L.P., Wood K.V. (2018). Click beetle luciferase mutant and near infrared naphthyl-luciferins for improved bioluminescence imaging. Nat. Commun..

[15] Iwano S., Sugiyama M., Hama H., Watakabe A., Hasegawa N., Kuchimaru T., Tanaka K.Z., Takahashi M., Ishida Y., Hata J. (2018). Single-cell bioluminescence imaging of deep tissue in freely moving animals. Science.

[16] Jathoul A., Law E., Gandelman O.A., Pule M., Tisi L., Murray J. (2012). Development of a pH-Tolerant Thermostable Photinus pyralis Luciferase for Brighter In Vivo Imaging.

[17] Stowe C.L., Burley T.A., Allan H., Vinci M., Kramer-Marek G., Ciobota D.M., Parkinson G.N., Southworth T.L., Agliardi G., Hotblack A. (2019). Near-infrared dual bioluminescence imaging in mouse models of cancer using infraluciferin. ELife.

[18] Steinhardt R.C., Rathbun C.M., Krull B.T., Yu J.M., Yang Y.H., Nguyen B.D., Kwon J., McCutcheon D.C., Jones K.A., Furche F. (2017). Brominated Luciferins Are Versatile Bioluminescent Probes. ChemBioChem.

[19] Liu M.D., Warner E.A., Morrissey C.E., Fick C.W., Wu T.S., Ornelas M.Y., Ochoa G.V., Zhang B.S., Rathbun C.M., Porterfield W.B. (2018). Statistical Coupling Analysis-Guided Library Design for the Discovery of Mutant Luciferases. Biochemistry.

[20] Adams S.T., Miller S.C. (2014). Beyond D-luciferin: Expanding the scope of bioluminescence imaging in vivo. Curr. Opin. Chem. Biol..

[21] Williams S.J., Prescher J.A. (2019). Building Biological Flashlights: Orthogonal Luciferases and Luciferins for In Vivo Imaging. Acc. Chem. Res..

[22] Liu S., Su Y., Lin M.Z., Ronald J.A. (2021). Brightening up Biology: Advances in Luciferase Systems for in Vivo Imaging. ACS Chem. Biol..

[23] Contag C.H., Bachmann M.H. (2002). Advances in vivo bioluminescence imaging of gene expression. Annu. Rev. Biomed. Eng..

[24] Nasu Y., Campbell R.E. (2018). Unnaturally aglow with a bright inner light. Science.

[25] Abe M., Nishihara R., Ikeda Y., Nakajima T., Sato M., Iwasawa N., Nishiyama S., Paulmurugan R., Citterio D., Kim S.B. (2019). Near-Infrared Bioluminescence Imaging with a through-Bond Energy Transfer Cassette. ChemBioChem.

[26] Weissleder R., Ntziachristos V. (2003). Shedding light onto live molecular targets. Nat. Med..

[27] Saito R., Kuchimaru T., Higashi S., Lu S.W., Kiyama M., Iwano S., Obata R., Hirano T., Kizaka-Kondoh S., Maki S.A. (2019). Synthesis and luminescence properties of near-infrared N-heterocyclic luciferin analogues for in vivo optical imaging. Bull. Chem. Soc. Jpn..

[28] Branchini B.R., Ablamsky D.M., Rosenberg J.C. (2010). Chemically Modified Firefly Luciferase Is an Efficient Source of Near-Infrared Light. Bioconjugate Chem..

[29] Branchini B.R., Southworth T.L., Fontaine D.M., Kohrt D., Florentine C.M., Grossel M.J. (2018). A Firefly Luciferase Dual Color Bioluminescence Reporter Assay Using Two Substrates to Simultaneously Monitor Two Gene Expression Events. Sci. Rep..

[30] Rathbun C.M., Porterfield W.B., Jones K.A., Sagoe M.J., Reyes M.R., Hua C.T., Prescher J.A. (2017). Parallel Screening for Rapid Identification of Orthogonal Bioluminescent Tools. Acs Cent. Sci..

[31] Branchini B.R., Southworth T.L., Fontaine D.M., Kohrt D., Welcome F.S., Florentine C.M., Henricks E.R., DeBartolo D.B., Michelini E., Cevenini L. (2017). Red-emitting chimeric firefly luciferase for in vivo imaging in low ATP cellular environments. Anal. Biochem..

[32] Shinde R., Perkins J., Contag C.H. (2006). Luciferin derivatives for enhanced in vitro and in vivo bioluminescence assays. Biochemistry.

[33] Evans M.S., Chaurette J.P., Adams S.T., Reddy G.R., Paley M.A., Aronin N., Prescher J.A., Miller S.C. (2014). A synthetic luciferin improves bioluminescence imaging in live mice. Nat. Methods.

[34] Simonyan H., Hurr C., Young C.N. (2016). A synthetic luciferin improves in vivo bioluminescence imaging of gene expression in cardiovascular brain regions. Physiol. Genom..

[35] Zheng Z., Li G.Y., Wu C.F., Zhang M.M., Zhao Y., Liang G.L. (2017). Intracellular synthesis of D-aminoluciferin for bioluminescence generation. Chem. Commun..

[36] Abakumov M., Kilpeläinen A., Petkov S., Belikov S., Klyachko N., Chekhonin V., Isaguliants M. (2019). Evaluation of cyclic luciferin as a substrate for luminescence measurements in in vitro and in vivo applications. Biochem. Biophys. Res. Commun..

[37] Parkins K.M., Dubois V.P., Hamilton A.M., Makela A.V., Ronald J.A., Foster P.J. (2018). Multimodality cellular and molecular imaging of concomitant tumour enhancement in a syngeneic mouse model of breast cancer metastasis. Sci. Rep..

[38] Zhang X., Lin C., Chan W., Liu K., Lu A., Lin G., Hu R., Shi H., Zhang H., Yang Z. (2019). Dual-Functional Liposomes with Carbonic Anhydrase IX Antibody and BR2 Peptide Modification Effectively Improve Intracellular Delivery of Cantharidin to Treat Orthotopic Hepatocellular Carcinoma Mice. Molecules.

[39] Zambito G., Gaspar N., Ridwan Y., Hall M.P., Shi C., Kirkland T.A., Encell L.P., Löwik C., Mezzanotte L. (2020). Evaluating Brightness and Spectral Properties of Click Beetle and Firefly Luciferases Using Luciferin Analogues: Identification of Preferred Pairings of Luciferase and Substrate for In Vivo Bioluminescence Imaging. Mol. Imaging Biol..

[40] Viviani V.R., Bevilaqua V.R., de Souza D.R., Pelentir G.F., Kakiuchi M., Hirano T. (2021). A very bright far-red bioluminescence emitting combination based on engineered railroad worm luciferase and 6′-amino-analogs for bioimaging purposes. Int. J. Mol. Sci..

[41] Anderson J.C., Chang C.H., Jathoul A.P., Syed A.J. (2019). Synthesis and bioluminescence of electronically modified and rotationally restricted colour-shifting infraluciferin analogues. Tetrahedron.

[42] Ikeda Y., Nomoto T., Hiruta Y., Nishiyama N., Citterio D. (2020). Ring-Fused Firefly Luciferins: Expanded Palette of Near-Infrared Emitting Bioluminescent Substrates. Anal. Chem..

[43] Saito-moriya R., Nakayama J., Kamiya G., Kitada N., Obata R., Maki S.A., Aoyama H. (2021). How to select firefly luciferin analogues for in vivo imaging. Int. J. Mol. Sci..

[44] Branchini B.R., Hayward M.M., Bamford S., Brennan P.M., Lajiness E.J. (1989). Naphthylluciferin and Quinolylluciferin-Green and Red-Light Emitting Firefly Luciferin Analogs. Photochem. Photobiol..

[45] Takakura H., Sasakura K., Ueno T., Urano Y., Terai T., Hanaoka K., Tsuboi T., Nagano T. (2010). Development of Luciferin Analogues Bearing an Amino Group and Their Application as BRET Donors. Chem.-Asian J..

[46] McCutcheon D.C., Porterfield W.B., Prescher J.A. (2015). Rapid and scalable assembly of firefly luciferase substrates. Org. Biomol. Chem..

[47] Mofford D.M., Reddy G.R., Miller S.C. (2014). Aminoluciferins Extend Firefly Luciferase Bioluminescence into the Near-Infrared and Can Be Preferred Substrates over D-Luciferin. J. Am. Chem. Soc..

[48] Harwood K.R., Mofford D.M., Reddy G.R., Miller S.C. (2011). Identification of Mutant Firefly Luciferases that Efficiently Utilize Aminoluciferins. Chem. Biol..

[49] Ji X., Adams S.T., Miller S.C. (2020). Bioluminescence imaging in mice with synthetic luciferin analogues. Methods Enzymol..

[50] Branchini B.R., Ablamsky D.M., Rosenman J.M., Uzasci L., Southworth T.L., Zimmer M. (2007). Synergistic mutations produce blue-shifted bioluminescence in firefly luciferase. Biochemistry.

