# Supplementary material for: Systematic Comparison of Beetle Luciferase-Luciferin Pairs as Sources of Near-Infrared Light for In Vitro and In Vivo Applications
Source: Int J Mol Sci. 2022 Feb 23;23(5):2451. doi: 10.3390/ijms23052451 (PMC8910387; doi:10.3390/ijms23052451)
Supplement: Supplementary file 1 [file ijms-23-02451-s001.zip › ijms-1604695-supplementary.pdf]

SUPPLEMENTARY MATERIALS

Systematic Comparison of Beetle Luciferase – Luciferin Pairs as Sources of Near-Infrared Light for *In Vitro* and *In Vivo* Applications

Bruce R. Branchini[1]*, Danielle M. Fontaine[1], Dawn Kohrt[1], Brian P. Huta[1], Allison R. Racela[1], Benjamin R. Fort[1], Tara L. Southworth[1] and Aldo Roda[2]

[1]Department of Chemistry, Connecticut College, New London, Connecticut 06320, United States; brbra@conncoll.edu

[2] Department of Chemistry "G. Ciamician", University of Bologna, Via Selmi 2, 40126 Bologna, Italy;

National Institute of Biostructures and Biosystems (INBB), Rome, Italy; aldo.roda@unibo.it,

*Correspondence: brbra@conncoll.edu; Tel.: +01-860-439-2479

CONTENTS

I. *Materials and Methods* S2

II. *Synthesis of NH_2-QLH_2* S4

III. *Synthesis* of OH-QLH_2 S7

IV *Synthesis of NH_2-NpLH2 and OH-NpLH2.* S9

V. *NMR Spectra* S12

VI. *IR Spectra* S26

VII. *Figures S7 -S11: Characterization of Substrates* S32

VIII. *Tables: Characterization of Substrates* S36

IX. *References* S37

I. MATERIALS AND METHODS

Anhydrous solvents: pyridine, toluene, tetrahydrofuran (THF), dichloromethane (DCM), and acetonitrile; methyl *tert*-butyl ether (MTBE), di-*tert*-butyl dicarbonate, 10% Pd/C, $Pd(OAc)_2$, Cs_2CO_3, CuI, pyridine HCl (dried *in vacuo* at 50 °C over anhydrous P_2O_5), DMSO-d_6, Celite 545, Na_2SO_4, $NaHPO_4$, NH_4OAc, KCl, HPLC-grade formic acid, HPLC-grade TFA, sulfolane, and $CDCl_3$, were obtained from Fisher Chemical (Hampton, NH). Chloroacetonitrile, S_2Cl_2, *N*-bromosuccinimide, D-cysteine HCl monohydrate, 4-dimethylaminopyridine (DMAP), and 5-[Di(1-adamantyl)phosphino]-1',3',5'-triphenyl-1'*H*-[1,4']bipyrazole (Ad-BGPhos) were purchased from Sigma-Aldrich (St. Louis, MO). 3-bromo-7-nitroquinoline was from Aurum Pharmatech LLC (Franklin Park, NJ) and 7-nitroquinolin-3-amine was from Enamine (Kiev, Ukraine). All reagents were used without further purification. All glassware was oven-dried and cooled under N_2 prior to use. Anhydrous Na_2SO_4 was used for drying organic extracts. Appel's salt (4,5-dichloro-1,2,3-dithiazolium chloride) was synthesized according to literature procedures.[1, 2] Microwave syntheses were carried out using a Biotage Initiator EXP US. All reaction products were purified using the Biotage Isolera One with gradients of hexanes and ethyl acetate and 25 μm SNAP Ultra HP-Sphere cartridges unless otherwise specified. All reactions and column fractions were monitored by TLC using glass-backed TLC plates (Merck silica gel 60 F_{254}). The plates were observed under 254 nm and 365 nm UV light. Reactions were also monitored by LC/MS using a Thermo Scientific Ultimate 3000 liquid chromatography system paired with a Thermo Scientific LTQ XL mass spectrometer utilizing a C_{18} 50 x 2.10 mm column (Phenominex). LC/MS solvent system consisted of ddH_2O/CH_3CN with a gradient elution of 3% CH_3CN to 95% CH_3CN over six min. The HPLC conditions for the retention times of the substrates (see Table S1) were: Hold at 16% B for 3 minutes, gradient from 16% B to 50% B from 3 to 37 minutes, gradient from 50% B to 95% B from 37 to 38 minutes. Hold at 95% B from 38 minutes to 43 minutes, gradient from 95% B to 16% B from 43 minutes to 45 minutes, and hold at 16% B from 45 minutes to 60 minutes. IR of the products were obtained using a Bruker PLATINUM-ATR ALPHA spectrometer compatible with OPUS 7.5 software. Enantiopurity was determined using a CHIRALCEL® OD-RH chiral column (5 μm particle size) following literature procedures.[3] High-resolution mass spectrometry (HRMS) ESI analyses were performed at the University of Illinois at Urbana-Champaign, Chemical Sciences Mass Spectrometry Laboratory. ^{1}H-NMR spectra were acquired at 500 MHz and ^{13}C-NMR spectra were acquired at 126 MHz at 295K with an Agilent DD2-500 MHz spectrometer. Coupling constants (*J*) given in Hz. Chemical shifts are provided in ppm. Reference values for residual solvents were taken as $\delta = 7.26$ ($CDCl_3$) and 2.51 (DMSO-d_6).

Fluorescence Emission Spectra. Spectra were obtained using a Horiba Jobin-Yvon *i*HR imaging spectrometer equipped with a liquid nitrogen-cooled CCD detector and slit widths were set to 1 nm. All spectra were corrected for the spectral response for the CCD using a correction curve provided by the manufacturer. Data were collected at 25 °C in a 0.8 mL quartz cuvette between 400-900 nm. Concentrations of the substrates were determined using a Perkin Elmer Lambda 365 UV/Vis Spectrophotometer and a 1 mL quartz cuvette.

Fluorescence Quantum Yields. Relative fluorescence quantum yields were determined by preparing solutions of the substrates in 50 mM $NaHCO_3$ at pH 11 with absorbance values of 0.02, 0.04, 0.06, 0.08, and 0.10 Au when measured at 370 nm. The emission integrals were plotted against the

absorbance at 370 nm. The slopes of the lines of fluorescein (in 0.1 M NaOH, $\Phi_{Fl} = 0.79$) and quinine sulfate (in 0.5 M H_2SO_4, $\Phi_{Fl} = 0.55$) were used to calculate the relative fluorescence quantum yields.

Protein Expression and Purification. Glutathione S-transferase (GST) fusion constructs of all enzymes in the pGEX-6P-2 bacterial expression vector were expressed in *Escherichia coli* strain BL21(DE3) pLysS as previously described.[4,5] Cultures (0.25 L in Luria–Bertani media with 0.1 mg/mL ampicillin) were grown in 1 L flasks at 37 °C to mid-log phase (A_{600} = 0.5-0.7) and then induced with 0.1 mM isopropyl-β-d-thiogalactopyranoside (IPTG) and incubated at 22 °C for 18-20 h. Cells were harvested by centrifugation at 4 °C and then frozen at −80 °C for 15 min. Cell pellets were resuspended in 25 mL of phosphate-buffered saline (PBS) containing 0.1 mM phenylmethylsulfonyl fluoride and 0.5 mM dithiothreitol (DTT). After the addition of lysozyme (2.5 mL of 10 mg/mL solution in PBS), the cells were lysed by sonication (three 10 s bursts at setting 3 of a Virsonic model 475 instrument) and treated with DNase (5 μg/mL) and RNase (10 μg/mL) for 10 min on ice. Triton X-100 was added to the lysates (1% final volume), and the whole-cell extracts were isolated by centrifugation at 20,000 x g for 45 min. Proteins were further purified using Glutathione Sepharose 4B affinity chromatography according to the manufacturer's instructions. During the purification, luciferases were released from GST by incubation with PreScission protease in 20 mM Tris-HCl (pH 7.4) containing 0.15 M NaCl, 1 mM EDTA and 1 mM DTT (CB; cleavage buffer) for 18-20 h at 4 °C with gentle mixing. Proteins were eluted with CB and were either flash frozen in liquid N_2 for long-term storage at −80 °C or stored at 4 °C in CB containing 0.8 M ammonium sulfate and 2% glycerol (CBA). Protein yields were determined to be 7-10 mg per 0.25 L protein preparation.

His-tag fusion constructs containing Mut51 and Fluc_red in the pET28a and pET16b bacterial expression vectors, respectively, were expressed in *Escherichia coli* strain BL21(DE3) pLysS. Cultures (0.25 L of Luria-Bertani media supplemented with 0.1 mg/mL ampicillin for the pET16b vector and 25 μg/mL kanamycin for the pET28a vector) were grown in 1 L flasks at 37 °C to mid log phase (A_{600} = 0.5-0.7), moved to a 22 °C incubator, allowed to equilibrate for 10 min, induced with 0.1 mM IPTG and incubated at 22 °C for 18-20 h. Cells were harvested by centrifugation at 4 °C and then frozen at -80 °C for 15 min. The cell pellet was resuspended in 25 mL of phosphate-buffered saline (PBS) containing 0.1 mM phenylmethylsulfonyl fluoride and 5 mM imidazole. After the addition of 2.5 mL lysozyme (10 mg/mL in PBS), the cells were lysed by sonication (three 10 s bursts at setting 3 of a Virsonic model 475 instrument) and treated with DNase (5 μg/mL) and RNase (10 μg/mL) for 5 min on ice. Triton X-100 was added (1% final volume) and the whole-cell extracts were isolated by centrifugation at 20,000 x g for 45 min. His-tagged fusion proteins were purified using Ni-NTA agarose affinity chromatography according to the manufacturer's instructions. Fractions eluted with 0.05-0.5 M imidazole were pooled (~6 mL) and dialyzed (3 changes, 1 L each) against CB. Protein yields were 5-10 mg per 0.25 L culture and storage conditions are described above.

***K_m* Measurements**. Values of K_m for the substrates/analogs were determined from BL activity assays in which measurements of maximal light intensities (bursts) were taken as estimates of initial velocities. The equipment used to make these measurements has been previously described.[6] Data were collected in 0.525 mL reactions in CBA, pH 7.4. Reactions were initiated by injection of 0.12 mL of a solution of Mg-ATP (9 mM). K_m values were determined using a nonlinear least squares method of the Enzyme Kinetics Pro software (SynTex), which fits data from the Michaelis–Menten equation to a rectangular hyperbola.[7]

Membrane Permeability. HEK293T cells were transfected as described in the main text and re-plated at 25,000 cells per well of 96-well black plates. The assay was performed as described,[3] except that bioluminescence was measured using the IVIS Spectrum III instrument and reported as the ratio of Ave. Radiance (p/s/cm^2/sr) from lysed (using PLB) to un-lysed cells.

Cell Culture and Transfection. HEK293T cells were grown in Dulbecco's Modified Eagles Medium supplemented with 10% fetal bovine serum. Cells were counted using a TC10 automated cell counter (BioRad, Hercules, CA) and plated at 1,250,000 cells per well in a 6-well plate and grown at 37 °C with 5% CO_2 for 4-6 h prior to transfection. One microgram of *luc-pNL3.1* plasmid DNA plus 1 µg pF4Ag empty vector in 0.125 mL OptiMEM (Invitrogen /Thermofisher, Waltham, MA) was added to 0.125 mL OptiMEM containing 6 µL Lipofectamine 2000 transfection reagent (Invitrogen/Thermofisher, Waltham, MA). The mixtures were incubated for 10 min at room temp and were added to the prepared HEK293T cells in 6-well plates.

Live Cell Imaging in transfected HEK293T cells. Transfected cells were grown for 20 h at 37 °C with 5% CO_2 and were released from the plate with 0.05% trypsin-EDTA (Gibco), resuspended in growth media, and counted. Each well of a black clear-bottom 96-well plate was seeded with 50,000 cells in 0.1 mL growth media and grown for an additional 24 h. For all series of substrates examined by each Luc, a separate set of quadruplicate wells was seeded for Nluc activity analysis using the Nano Glo Luciferase Assay Kit (Promega, Madison WI). Growth media was removed and replaced with 0.05 mL DMEM without phenol red plus 10% FBS. Nluc assay buffer mix (0.05 mL) was added, mixed by pipetting and BL was measured after a 3 min incubation at room temp. BL was measured using an IVIS Spectrum III (Perkin Elmer) with the auto exposure, FOV B, and OPEN filter settings. For the Luc-substrate assays, growth media was removed and 0.1 mL of 0.5 mM solutions of LH_2 or analogs in assay buffer (50 mM Tricine (pH 7.4), growth media (1:1, v/v), and 5 µM ATP) were added to each well. After a 30 sec incubation, BL was measure at 37 °C. Signals were monitored for 8 min with a measurement at 30 sec intervals. Data were analyzed with the Living Image 4.7 Software (Perkin Elmer) after selecting the appropriate region of interest (ROI). The highest average Luc - substrate activity recorded within the first 5 min was used to calculate the final average radiance (p/s/cm^2/sr). In order to account for daily variations in transfection efficiencies, the reported radiance values were calculated from the mean ± standard deviation of BL signals corrected by the respective Nluc activities. Each experiment was repeated at least 3 times. , BL was measured with an IVIS Spectrum III (Perkin Elmer, Waltham, MA) using the auto setting, FOV B, and filters set at OPEN, 520 ± 20 nm, 570 ± 20 nm, 620 ± 20 nm, 670 ± 20 nm, 710 ± 20 nm, 755 ± 15 nm and 790 ± 20 nm.

II. SYNTHESIS OF NH_2-QLH_2

Bis-tert-butyl (7-nitroquinolin-3-yl)carbamate (1). To a 100 mL round bottom flask equipped with a stir bar was added 1.0 g (5.29 mmol) of 7-nitroquinolin-3-amine, 388 mg (3.17 mmol) DMAP, and 67 mL anhydrous THF. The mixture was placed under an argon atmosphere and stirred for 5 min. Di-*tert*-butyl-dicarbonate (7.29 mL, 6.92 mg, 31.7 mmol) was added to the flask dropwise over 5 min. As the reaction was heated to 50 °C with stirring, a white precipitate formed, and after 2.5 h, the mixture became a clear orange solution. The solvent was removed under reduced pressure and the resulting solid was

purified by flash chromatography yielding (**1**) as a white solid (1.75 g, 4.50 mmol, 85%). m.p. 158-159 °C; R_f = 0.29 (25% EtOAc/hexanes); ^{1}H NMR: (500 MHz, $CDCl_3$) δ 9.02 (d, *J* = 2.0 Hz, 1H); 8.86 (d, *J* = 2.5 Hz, 1H); 8.34 (dd, *J* = 9.1, 2.2 Hz, 1H); 8.04 (d, *J* = 2.5 Hz, 1H); 7.98 (d, *J* = 8.8 Hz, 1H); 1.44 (s, 18H). ^{13}C NMR: (126 MHz, $CDCl_3$) δ 152.96, 150.84, 148.27, 145.45, 135.30, 133.14, 131.00, 129.43, 125.60, 120.67, 84.30, 27.89. HRMS (ESI$^+$) calculated for $C_{19}H_{24}N_3O_6$: 390.1665 $[M+H]^+$, found: 390.1652.

Bis-tert-butyl (7-aminoquinolin-3-yl)carbamate (2). H_2 was bubbled through a 1:1 mixture of THF and MeOH for ~30 min. To a 100 mL round bottom flask equipped with a stir bar was added **1** (1.75 g, 4.5 mmol) and 42 mL of the H_2-solvent mixture with stirring. The flask was fitted with a H_2-balloon. When all of the solid was dissolved, 10% Pd/C (116 mg, 1.09 mmol) was added. The reaction was allowed to stir at room temp, under hydrogen atmosphere, for 3 d and then was filtered through Celite 545 Filter Aid. The filtrate was collected and concentrated under reduced pressure to yield (**2**) as an off-white solid (1.61 g, 4.48 mmol, 100%). m.p. 184 – 186 °C; R_f = 0.32 (25% EtOAc/hexanes); ^{1}H NMR: (500 MHz, $CDCl_3$) δ 8.52 (d, *J* = 2.4 Hz, 1H); 7.73 (d, *J* = 2.4 Hz, 1H); 7.59 (d, *J* = 8.8 Hz, 1H); 7.20 (d, *J* = 2.4 Hz, 1H); 6.99 (dd, *J* = 8.6, 2.3 Hz, 1H); 4.12 (s, 2H); 1.40 (s, 18H). ^{13}C NMR: (126 MHz, $CDCl_3$) δ 151.52, 150.65, 148.60, 148.00, 133.38, 129.91, 129.02, 121.45, 118.98, 109.22, 83.22, 27.90. HRMS (ESI$^+$) calculated for $C_{19}H_{26}N_3O_4$: 360.1923 $[M+H]^+$, found: 360.1911.

(Z)-bistert-butyl (7-((4-chloro-5H-1,2,3-dithiazol-5-ylidene)amino)quinolin-3-yl)carbamate (3). Anhydrous DCM (45 mL) was added to a 100 mL round bottom flask equipped with a stir bar, containing **2** (1.61 g, 4.48 mmol), and under an argon atmosphere. The contents were stirred at room temp until the solid was completely dissolved. DCM-washed Appel's salt (1.03 g, 4.93 mmol) was added and stirring was continued under argon for 1 h. The reaction was diluted with EtOAc (75 mL) and washed with ddH_2O (3 x 30 mL). The water washes were combined and extracted with EtOAc (6 x 20 mL). The combined organic layers were washed with brine (1 x 25 mL), and dried over Na_2SO_4, and concentrated *in vacuo*. Purification via flash chromatography, afforded **3** as an orange solid (813 mg, 2.06 mmol, 46%). m.p. 155 – 156 °C; R_f = 0.70 (25% EtOAc/hexanes); ^{1}H NMR: (500 MHz, $CDCl_3$) δ 8.72 (d, *J* = 2.4 Hz, 1H); 7.98 (d, *J* = 2.0 Hz, 1H); 7.97 (s, 1H); 7.92 (d, *J* = 8.8 Hz, 1H); 7.47 (dd, *J* = 8.8, 1.9 Hz, 1H); 1.45 (s, 18H). ^{13}C NMR: (126 MHz, $CDCl_3$) δ 160.06, 152.60, 151.30, 150.90, 148.10, 147.00, 134.08, 132.77, 129.81, 126.20, 122.90, 115.60, 83.92, 27.93. HRMS (ESI$^+$) calculated for $C_{21}H_{24}N_4O_4S_2Cl$: 495.0927 $[M+H]^+$, found: 495.0918. Additionally, the singly-protected product ((*Z*)-*tert*-butyl (7-((4-chloro-5*H*-1,2,3-dithiazol-5-ylidene)amino)quinolin-3-yl)carbamate) was yielded as yellow-orange solid (805 mg, 1.63 mmol, 36%).

7-aminothiazolo[4,5-h]quinoline-2-carbonitrile (4). To a 20 mL Biotage microwave reaction tube was added **3** (250 mg, 0.504 mmol) and 17 mL sulfolane. The tube was sealed and microwaved at 150 °C for 7 h. The mixture was diluted with 170 mL of ddH_2O and extracted with MTBE (12 x 20 mL). The combined organic layers were washed with ddH_2O (6 x 30 mL), brine (1 x 30 mL), and dried over Na_2SO_4. The product was purified via flash chromatography, affording **4** as a yellow solid (53.9 mg, 120 mmol, 24%). m.p. 240 °C, dec.; R_f = 0.47 (25% EtOAc/hexanes); ^{1}H NMR: (500 MHz, DMSO-d_6) δ 8.50 (d, *J* = 2.9 Hz, 1H); 8.06 (d, *J* = 8.8 Hz, 1H); 7.87 (d, *J* = 9.3 Hz, 1H); 7.35 (d, *J* = 2.4 Hz, 1H); 6.18 (s, 2H). ^{13}C NMR: (126 MHz, DMSO-d_6) δ 149.57, 145.52, 143.38, 136.48, 134.45, 133.78, 129.47,

127.93, 122.65, 114.29, 113.21. HRMS (ESI$^+$) calculated for $C_{11}H_7N_4S$: 227.0391 [M+H]$^+$, found: 227.0387.

(S)-2-(7-aminothiazolo[4,5-h]quinolin-2-yl)-4,5-dihydrothiazole-4-carboxylic acid (5). To a 75 mL conical flask equipped with a stir bar was added **4** (65 mg, 0.286 mmol) and argon-bubbled MeOH (3.8 mL). The mixture was stirred at room temp for 15 min and 3.8 mL of a solution of D-cysteine HCl monohydrate (46.5 mg cysteine, 11.4 mL of 50 mM argon-bubbled $NaHPO_4$ buffer, pH 8) was added to the suspension. The suspension was stirred for 1 h under an argon atmosphere and an additional 3.8 mL of methanol and 3.8 mL of the D-cysteine solution was added. The reaction was stirred for 1 hr and an additional 3.8 mL of methanol and 3.8 mL of the D-cysteine solution was added and stirring continued for 1 hour. The reaction mixture was diluted with 15 mL of 50 mM sodium phosphate buffer, pH 8.0 and washed with EtOAc (1 x 15 mL). The aqueous layer was acidified to pH 2 (litmus) with 1 N HCl and extracted with EtOAc (4 x 15 mL). The organic layers were combined, washed with ddH_2O (3 x 15 mL), brine (1 x 10 mL), and dried over Na_2SO_4. The solvent was removed *in vacuo* affording **5, NH_2-QLH$_2$**, as an orange solid (45 mg, 0.136 mmol, 48% yield). m.p. 197 °C, dec.; ^{1}H NMR: (500 MHz, DMSO-d_6) δ 13.18 (br, 1H); 8.50 (d, *J* = 2.9 Hz, 1H); 8.01 (d, *J* = 8.8 Hz, 1H); 7.77 (d, *J* = 8.8 Hz, 1H); 7.33 (d, *J* = 2.4 Hz, 1H); 6.03 (br, 2H); 5.43 (dd, *J* = 9.6, 8.1 Hz, 1H); 3.78 (dd, *J* = 11.2, 9.8 Hz, 1H); 3.69 (dd, *J* = 11.1, 8.1 Hz, 1H). ^{13}C NMR: (126 MHz, DMSO-d_6) δ 171.67, 164.84, 158.54, 150.27, 144.73, 143.19, 135.53, 135.18, 128.85, 126.67, 122.74, 113.43, 78.57. HRMS (ESI$^+$) calculated for $C_{14}H_{11}N_4O_2S_2$: 331.0323 [M+H]$^+$, found: 331.0329.

NH_2-QLH$_2$ potassium salt (6). To a 15 mL conical tube containing 8 mL of ddH_2O was added 10 µL of 0.5 M (5.0 µmol) aq. KCl and the solution was cooled on ice in the dark. Compound **5** (16.5 mg, 50 µmol) was added and the mixture was vortexed. The resulting suspension was titrated carefully with 0.1 mL aliquots of 50 mM aq. $KHCO_3$ making sure that the pH remained below 7.0 (litmus). After the addition of each aliquot of aq. $KHCO_3$, the suspension was vortexed and the solid gradually dissolved until ~95% of the solid was in solution. After the addition of 0.975 mL of the aq. $KHCO_3$ solution (48.8 µmol), the pH was ~6.9 (litmus) and the solution was flash frozen in liquid N_2 and lyophilized overnight to yield **6**, the K^+ salt of NH_2-QLH$_2$, as an orange powder (18.5 mg, 49.2 µmol, 98% (8% overall yield), 98.6% ee); solubility of **6** in PBS is ~10mg/mL.

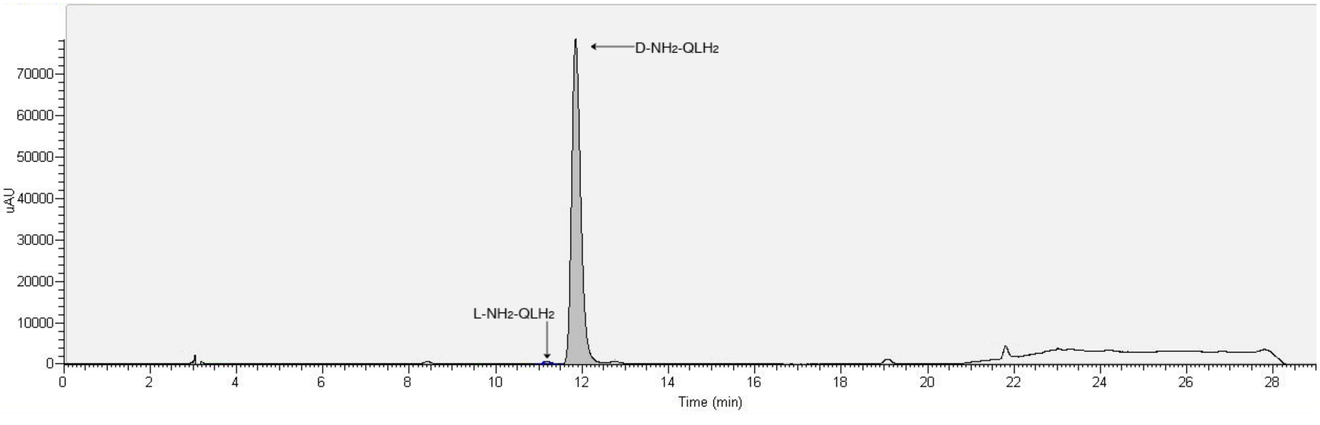

Figure S1. Enantiopurity analysis of NH_2-QLH$_2$ (K^+ salt), ee = 98.6%.

III. SYNTHESIS OF OH-QLH_2

3-methoxy-7-nitroquinoline (7). A 6 mL hydrolysis tube was charged with $Pd(OAc)_2$ (5.7 mg, 0.025 mmol), Ad-BGPhos (33.3 mg, 0.050 mmol), and Cs_2CO_3 (1.22 g, 3.75 mmol) and the contents were dried under vacuum for 10 minutes and 3-bromo-7-nitroquinoline (632.5 mg, 2.5 mmol), degassed MeOH (2.2 mL, 1.69 g, 52.75 mmol), and toluene (2.2 mL) were added. After one freeze-pump-thaw cycle, the mixture was warmed to room temp and submerged in an 80 °C oil bath, producing dark red color solution. After 5 h, the reaction was cooled to room temp and the resulting solid was suspended in EtOAc (25 mL), sonicated, and filtered through a Celite pad. The crude product was purified by flash chromatography yielding **7** as light yellow solid (270 mg, 1.32 mmol, 53%). m.p. 161 – 163 °C; R_f = 0.43 (50% EtOAc/hexanes); ^1H NMR: (500 MHz, $CDCl_3$) δ 8.96 (d, *J* = 2.5 Hz, 1H); 8.80 (d, *J* = 2.9 Hz, 1H); 8.30 (dd, *J* = 9.1 Hz, 2.3 Hz); 7.85 (d, *J* = 8.8 Hz, 1H); 7.44 (d, *J* = 3.0 Hz, 1H); 4.01 (s, 3H). ^{13}C NMR: (126 MHz, $CDCl_3$) δ 155.23, 147.10, 146.13, 141.43, 132.76, 127.87, 125.36, 120.97, 111.80, 55.86. HRMS (ESI^+) calculated for $C_{10}H_9N_2O_3$: 205.0613 $[M+H]^+$, found: 205.0606.

3-methoxyquinolin-7-amine (8). To a 100 mL round bottom flask equipped with a stir bar was added **7** (490 mg, 2.4 mmol), followed by 78 mL of H_2-bubbled THF:MeOH (1:1). The solution was stirred under a H_2 atmosphere and 10% Pd/C (61.8 mg, 0.576 mmol) was added. The reaction was allowed to stir at room temp overnight. The mixture was filtered through a Celite pad and concentrated under reduced pressure to yield **8** as a dark yellow solid (420 mg, 2.4 mmol) that was used without further purification. m.p. 148 °C, dec.; R_f = 0.15 (50% EtOAc/hexanes); ^1H NMR: (500 MHz, $CDCl_3$) δ 8.55 (d, *J* = 3.0 Hz, 1H); 7.53 (d, *J* = 8.8 Hz, 1H); 7.30 (d, *J* = 2.9 Hz, 1H); 7.21 (d, *J* = 2.5 Hz, 1H); 6.98 (dd, *J* = 8.6, 2.3 Hz, 1H); 3.98 (s, 3H); 3.71 (br, 2H). ^{13}C NMR: (126 MHz, $CDCl_3$) δ 151.19, 145.43, 144.98, 144.11, 127.62, 122.37, 119.21, 113.37, 109.99, 55.53. HRMS (ESI^+) calculated for $C_{10}H_{11}N_2O$: 175.0867 $[M+H]^+$, found: 175.0867.

8-bromo-3-methoxyquinolin-7-amine (9). To a 100 mL round bottom flask equipped with a stir bar was added **8** (375 mg, 2.153 mmol), NH_4OAc (16.6 mg, 0.215 mmol), and anhydrous CH_3CN (60 mL). The reaction was cooled to -5 °C with an ice-KCl bath. To the cloudy mixture was added dropwise over 90 minutes a solution of *N*-bromosuccinimide (402.3 mg, 2.260 mmol, dissolved in 16.5 mL of anhydrous CH_3CN). The reaction was stirred for 45 min and the solvent was removed under reduced pressure and the resulting solid was purified via flash chromatography to afford **9** as a bright yellow solid (355 mg, 1.403 mmol, 65%). m.p. 159 – 164 °C; R_f = 0.42 (50% EtOAc/hexanes); ^1H NMR: (500 MHz, DMSO-d_6) δ 8.51 (d, *J* = 2.9 Hz, 1H); 7.64 (d, *J* = 2.9 Hz, 1H); 7.59 (d, *J* = 8.8 Hz, 1H); 7.14 (d, *J* = 8.8 Hz, 1H); 5.75 (br, 2H); 3.85 (s, 3H). ^{13}C NMR: (126 MHz, DMSO-d_6) δ 151.22, 145.82, 143.56, 141.72, 127.11, 122.42, 119.67, 114.99, 102.85, 56.03. HRMS (ESI^+) calculated for $C_{10}H_{10}N_2OBr$: 252.9977 $[M+H]^+$, found: 252.9969.

(Z)-8-bromo-N-(4-chloro-5H-1,2,3-dithiazol-5-ylidene)-3-methoxyquinolin-7-amine (10). To a 100 mL round bottom flask equipped with a stir bar was added **9** (306.7 mg, 1.212 mmol) and anhydrous DCM (12 mL) under an argon atmosphere. The solution was stirred at room temp and DCM-washed Appel's salt (303.3 mg, 1.454 mmol) was added and stirring was continued for 1 h. An additional 12 mL

of anhydrous DCM was added twice with 2 h stirring between each addition. TLC analysis had indicated the reaction had not gone to completion, so an additional 364 mg (1.75 mmol) of the Appel's salt was added and the reaction was allowed to stir overnight. The suspension was diluted with ddH_2O (100 mL) and extracted with EtOAc (4 x 25 mL). The combined organic layers were washed with brine (1 x 15 mL) and dried over Na_2SO_4. The product was purified by flash chromatography to afford **10** as an orange solid (465 mg, 1.202 mmol, 99%). m.p. 145 – 147 °C, dec.; R_f = 0.45 (50% EtOAc/hexanes); ^{1}H NMR: (500 MHz, $CDCl_3$) δ 8.80 (d, *J* = 3.0 Hz, 1H); 7.77 (d, *J* = 8.3 Hz, 1H); 7.40 (d, *J* = 2.9 Hz, 1H); 7.25 (d, *J* = 8.3 Hz, 1H); 3.98 (s, 3H). ^{13}C NMR: (126 MHz, $CDCl_3$) δ 161.94, 153.61, 149.31, 147.38, 145.64, 141.39, 128.06, 127.84, 119.01, 114.79, 112.89, 55.76. HRMS (ESI$^+$) calculated for $C_{12}H_8N_3OS_2ClBr$: 387.8981 $[M+H]^+$, found: 387.8966.

7-methoxythiazolo[4,5-h]quinoline-2-carbonitrile (11). To a 25 mL round bottom flask equipped with a condenser and a stir bar was added **10** (160 mg, 0.412 mmol) and CuI (118 mg, 0.617 mmol) under an argon atmosphere. Anhydrous pyridine (12 mL) was added and the mixture was refluxed at 130 °C for 90 min. The solvent was removed under reduced pressure and the resulting black solid was added to 50 mL EtOAc and stirred vigorously for 15 min. The undissolved material was allowed to settle and the solution was decanted, washed with ddH_2O (2 x 10 mL) and with brine (1 x 10 mL), dried over Na_2SO_4, and the solvent was removed under reduced pressure. The product was purified via flash chromatography, yielding **11** as a brown, flakey solid (87.7 mg, 0.363 mmol, 88%). m.p. 208 °C, dec.; R_f = 0.35 (35% EtOAc/hexanes); ^{1}H NMR: (500 MHz, DMSO-d_6) δ 8.78 (d, *J* = 2.4 Hz, 1H); 8.28 (d, *J* = 8.8 Hz, 1H); 8.14 (d, *J* = 8.8 Hz, 1H); 8.09 (d, *J* = 2.9 Hz, 1H); 3.98 (s, 3H). ^{13}C NMR: (126 MHz, DMSO-d_6) δ 155.30, 151.12, 144.88, 137.37, 136.26, 135.92, 128.71, 123.37, 115.40, 113.98, 56.47. HRMS (ESI$^+$) calculated for $C_{12}H_8N_3OS$: 242.0388 $[M+H]^+$, found: 242.0379.

7-hydroxythiazolo[4,5-h]quinoline-2-carbonitrile (12). Pyridine HCl (3.35 g, 29.0 mmol) and **11** (100 mg, 0.414 mmol) were mixed together and added to a 2-5 mL Biotage microwave vessel, which was flushed with argon, sealed, and microwaved at 200 °C for 20 min. The yellow/brown mixture was added to 5 mL of 1 N HCl and extracted with 1:1 DCM:EtOAc (5 x 15 mL). The combined organic layers were washed with ddH_2O (1 x 10 mL), brine (1 x 10 mL), and dried over Na_2SO_4. The product was purified via flash chromatography to afford **12** as an orange solid (41.2 mg, 0.182 mmol, 44%). m.p. 228 °C, dec.; R_f = 0.41 (50% EtOAc/hexanes); ^{1}H NMR: (500 MHz, DMSO-d_6) δ 10.88 (s, 1H); 8.67 (d, *J* = 2.4 Hz, 1H); 8.18 (d, *J* = 8.8 Hz, 1H); 8.05 (d, *J* = 8.8 Hz, 1H); 7.77 (d, *J* = 2.4 Hz, 1H). ^{13}C NMR: (126 MHz, DMSO-d_6) δ 153.72, 150.75, 144.48, 136.71, 136.04, 135.65, 129.03, 128.46, 122.99, 117.93, 114.05. HRMS (ESI$^+$) calculated for $C_{11}H_6N_3OS$: 228.0232 $[M+H]^+$, found: 228.0225.

(S)-2-(7-hydroxythiazolo[4,5-h]quinolin-2-yl)-4,5-dihydrothiazole-4-carboxylic acid (13). To a 50 mL round bottom flask equipped with a stir bar was added **12** (19.4 mg, 0.085 mmol), argon-bubbled MeOH (12 mL), and 6 mL of argon-bubbled $NaHPO_4$ buffer, pH 8. The contents were stirred at room temp under an argon atmosphere and 6 mL of a solution of D-cysteine HCl monohydrate (14.9 mg, 0.085 mmol; in $NaHPO_4$ buffer, pH 8) was added. The reaction was stirred for 30 min and diluted with 24 mL of 50 mM sodium phosphate buffer, pH 8.0 and washed with EtOAc (1 x 10 mL). The aqueous layer was acidified to pH 2 (litmus) with 1 N HCl and extracted with EtOAc (5 x 15 mL). The combined organic layers were washed with ddH_2O (3 x 15 mL), brine (1 x 10 mL), and dried over Na_2SO_4. The

product was concentrated *in vacuo*, affording **13**, **OH-QLH$_2$**, as an orange solid (27.7 mg, 0.083 mmol, 98% yield, 13% overall). m.p. 191 °C, dec.; ^{1}H NMR: (500 MHz, DMSO-d_6) δ 13.22 (br, 1H); 10.71 (br, 1H); 8.67 (d, *J* = 2.4 Hz, 1H); 8.14 (d, *J* = 8.8 Hz, 1H); 7.96 (d, *J* = 8.8 Hz, 1H); 7.74 (d, *J* = 2.5 Hz, 1H); 5.45 (dd, *J* = 9.8, 8.3 Hz, 1H); 3.80 (dd, *J* = 11.3, 9.8 Hz, 1H); 3.71 (dd, *J* = 11.3, 8.4 Hz, 1H). ^{13}C NMR: (126 MHz, DMSO-d_6) δ 171.60, 164.90, 160.08, 152.99, 151.47, 144.19, 137.32, 135.14, 128.45, 127.29, 123.11, 117.85, 78.64, 35.35. HRMS (ESI$^+$) calculated for $C_{14}H_{10}N_3O_3S_2$: 332.0164 [M+H]$^+$, found: 332.0152.

OH-QLH$_2$ potassium salt (14). To a 15 mL conical tube containing 5 mL of ddH_2O was added 10 µL of 0.6 M (6.0 µmol) aq. KCl and the solution was cooled on ice in the dark. Compound **13** (20 mg, 60.4 µmol) was added and the mixture was vortexed. The resulting suspension was titrated carefully with 0.1 mL aliquots of 60 mM aq. $KHCO_3$ making sure that the pH remained below 7.0 (litmus). After the addition of each aliquot of aq. $KHCO_3$, the suspension was vortexed and the solid gradually dissolved until ~95% of the solid was in solution. After the addition of 0.975 mL of the aq. $KHCO_3$ solution (58.9 µmol), the pH was ~6.9 (litmus) and the solution was flash frozen in liquid N_2 and lyophilized overnight to yield **14**, the K^+ salt of OH-QLH$_2$, as an orange powder (22 mg, 58.3 µmol, 97% (13% overall yield), 97.2% ee). The solubility of the **14** in PBS is ~25 mg/mL.

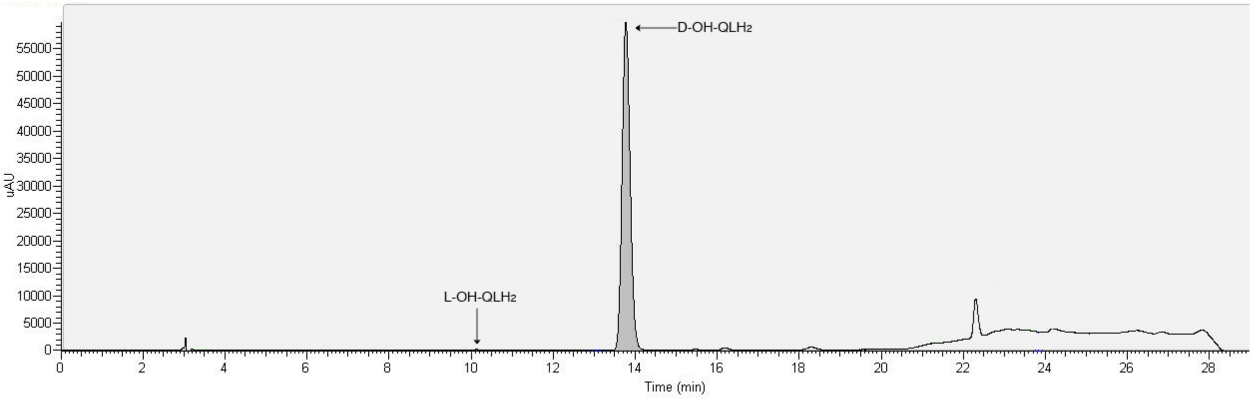

Figure S2. Enantiopurity analysis of OH-QLH$_2$ (K^+ salt), ee = 97.2%.

IV. SYNTHESIS OF NH$_2$-NpLH2 AND OH-NpLH2

7-aminonaphtho[2,1-*d*]thiazole-2-carbonitrile and 7-hydroxynaphtho[2,1-*d*]thiazole-2-carbo-nitrile, the precursors to NH$_2$-NpLH2 and OH-NpLH2, respectively, were prepared according to literature procedures.[3] The condensation reactions of the precursors and D-cysteine in the final step to the luciferin analogs were performed by a previously described method.[8] We note that in the case of the NH$_2$-NpLH2 synthesis, the reported yield of 29% for the condensation step was improved to 87%. Correspondingly, for the OH-NpLH2 synthesis, the reported yield of 28% was improved to 91%. In addition, the alternative procedure did not produce any of the reported dehydroluciferin contaminate.

OH-NpLH2

(S)-2-(7-hydroxynaphtho[2,1-d]thiazol-2-yl)-4,5-dihydrothiazole-4-carboxylic acid (OH-NpLH2). To a 25 mL round bottom flask equipped with a stir bar was added 7-hydroxynaphtho[2,1-*d*]thiazole-2-carbonitrile (14.9 mg, 0.066 mmol) and 6 mL of degassed MeOH. The mixture was stirred at room temp for 15 min and 6 mL of a solution of D-cysteine HCl monohydrate (12.8 mg, 0.073 mmol; in $NaHPO_4$ buffer, pH 8) was added dropwise over 5 min. The suspension was allowed to stir under an argon atmosphere for 30 min before being diluted with 6 mL of 50 mM degassed $NaHPO_4$ buffer at pH 8. The suspension was acidified to pH 2 (litmus) using 1 N HCl and extracted with EtOAc (6 x 10 mL). The combined organic layers were washed with ddH_2O (4 x 25 mL), brine (1 x 10 mL), and dried over solid Na_2SO_4. The product was concentrated under reduced pressure to afford **OH-NpLH2** as a yellow solid (19.8 mg, 0.060 mmol, 91%, 95% ee). ^{1}H NMR: (500 MHz, DMSO-d_6) δ 13.20 (br, 1H); 10.12 (br, 1H); 8.60 (d, *J* = 8.8 Hz, 1H); 8.02 (d, *J* = 8.8 Hz, 1H); 7.85 (d, *J* = 8.8 Hz, 1H); 7.34 (d, *J* = 2.5 Hz, 1H); 7.25 (dd, *J* = 8.8, 2.4 Hz, 1H); 5.43 (dd, *J* = 9.8, 8.3 Hz, 1H); 3.79 (dd, *J* = 11.3, 9.7 Hz, 1H); 3.70 (dd, *J* = 11.0, 8.1 Hz, 1H). ^{13}C NMR: (126 MHz, DMSO-d_6) δ 171.62, 164.65, 157.90, 157.35, 149.62, 134.23, 133.87, 127.70, 127.58, 122.33, 121.36, 120.00, 111.41, 78.53, 35.29. HRMS (ESI$^+$) calculated for $C_{15}H_{11}N_2O_3S_2$: 331.0211 $[M+H]^+$, found: 331.0201.

NH_2-LH2

(S)-2-(7-aminonaphtho[2,1-d]thiazol-2-yl)-4,5-dihydrothiazole-4-carboxylic acid (NH_2-NpLH2). To a 25 mL round bottom flask equipped with a stir bar was added 7-aminonaphtho[2,1-*d*]thiazole-2-carbonitrile (50 mg, 0.222 mmol), 30 mL of degassed MeOH, and 24 mL of degassed 50 mM $NaHPO_4$ buffer, pH 8. The mixture was stirred at room temp under an argon atmosphere for 15 min and a solution of D-cysteine HCl monohydrate (42.9 mg, 0.244 mmol; in 50 mM degassed $NaHPO_4$ buffer, pH 8) was added dropwise over 5 min. The reaction was allowed to stir at room temp overnight before being diluted with an additional 30 mL of 50 mM degassed $NaHPO_4$ buffer at pH 8. The suspension was acidified to pH 2 (litmus) using 1 N HCl and extracted with EtOAc (6 x 25 mL). The combined organic layers were washed with ddH_2O (3 x 50 mL), brine (1 x 25 mL), and dried over solid Na_2SO_4. The product was concentrated under reduced pressure, yielding **NH_2-NpLH2** as an orange solid (63.9 mg, 0.194 mmol, 87%, 98% ee). ^{1}H NMR: (500 MHz, DMSO-d_6) δ 7.89 (d, *J* = 9.3 Hz, 1H); 7.87 (d, *J* = 8.3 Hz, 1H); 7.66 (d, *J* = 8.8 Hz, 1H); 7.07 (dd, *J* = 8.6, 2.2 Hz, 1H); 6.99 (d, *J* = 2.4 Hz); 5.81 (br, 2H); 5.41 (dd, *J* = 9.8, 8.3 Hz, 1H); 3.77 (dd, *J* = 11.3, 9.8 Hz, 1H); 3.68 (dd, *J* = 11.1, 8.1 Hz, 1H). ^{13}C NMR: (126 MHz, DMSO-d_6) δ 171.72, 164.66, 156.29, 149.11, 148.54, 134.69, 134.22, 127.01, 126.93, 121.00, 119.24, 119.11, 108.07, 78.49, 35.21. HRMS (ESI$^+$) calculated for $C_{15}H_{12}N_3O_2S_2$: 330.0371 $[M+H]^+$, found: 330.0360.

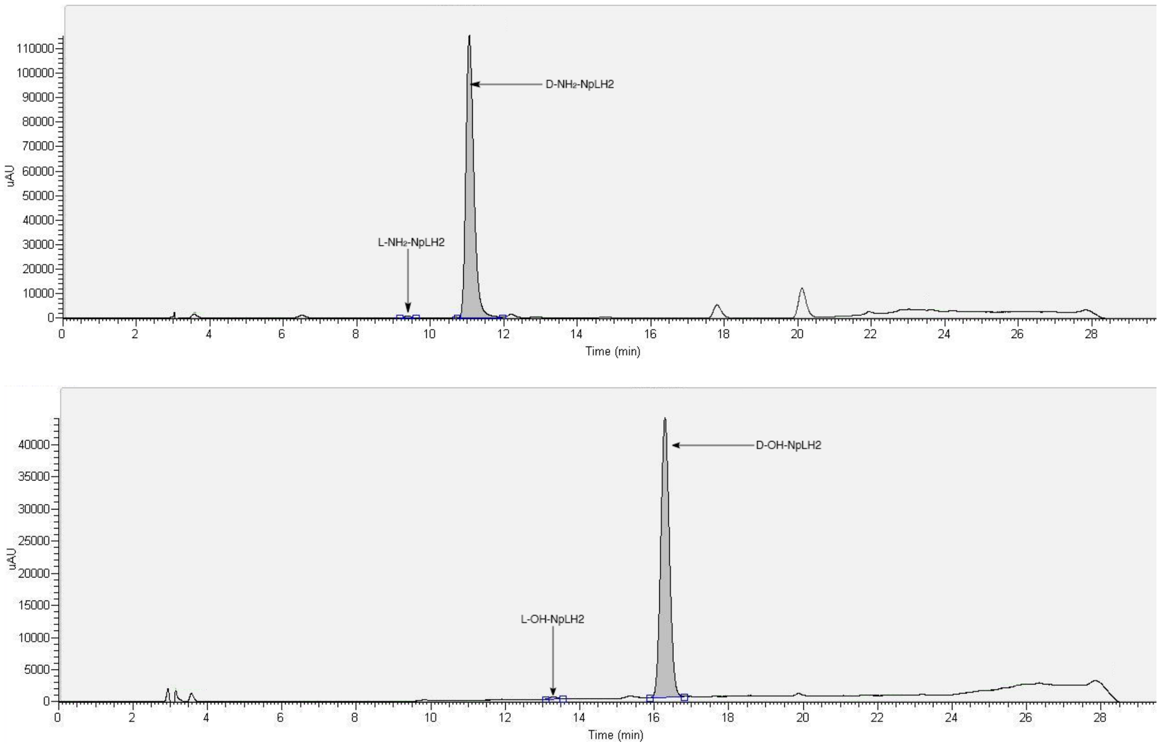

Figure S3. Enantiopurity analysis of NH_2-NpLH2. (Top) Chiral HPLC trace of our synthetic NH_2-NpLH2, ee = 98%; and (Bottom) our synthetic OH-NpLH2, ee = 95% .

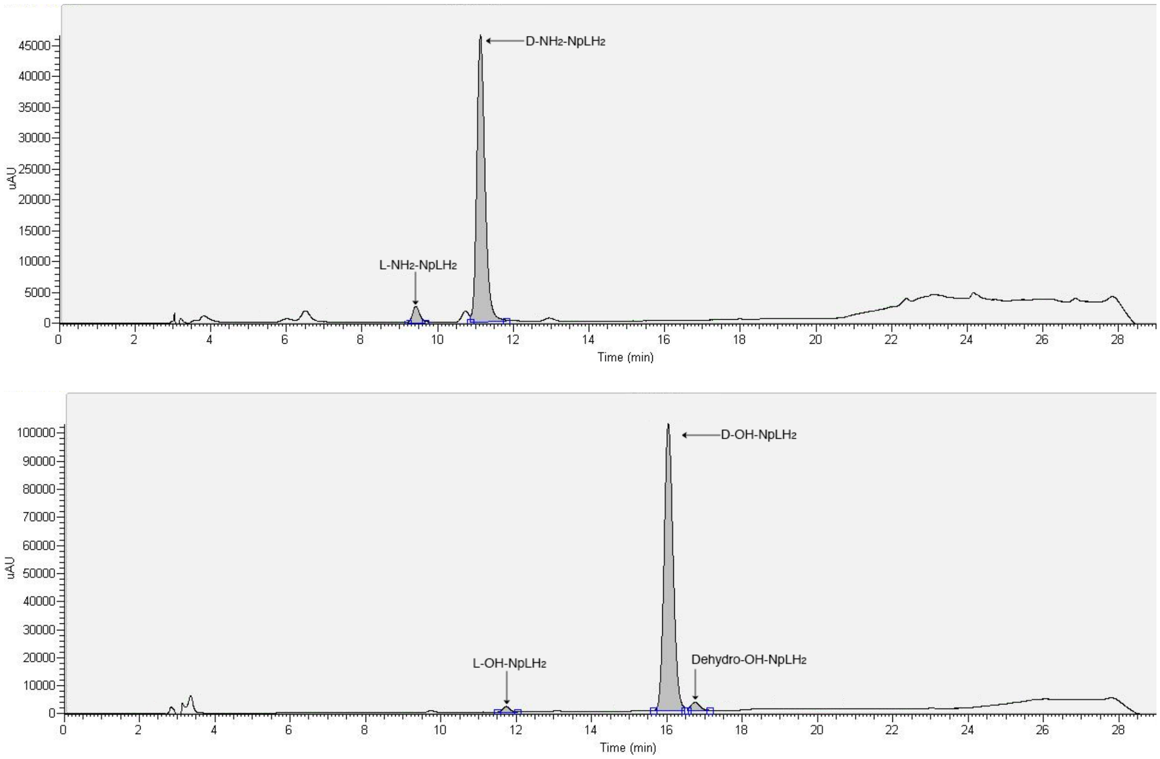

Figure S4. Enantiopurity analysis of OH-NpLH2. Chiral HPLC traces of synthetic samples obtained from Promega of (Top) NH_2-NpLH2, ee = 89%; and (Bottom) OH-NpLH2, ee = 96%. Note the presence of the dehydroluciferin contaminant, a strong inhibitor of BL.

V. NMR SPECTRA

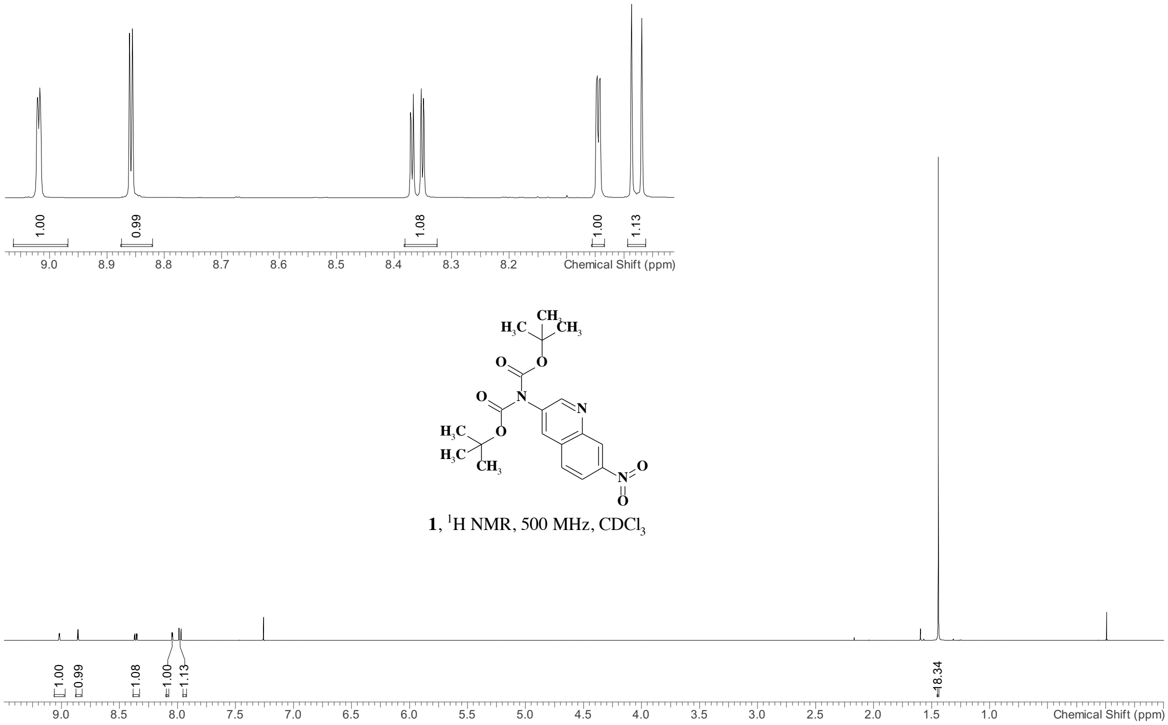

1, ^{1}H NMR, 500 MHz, $CDCl_3$

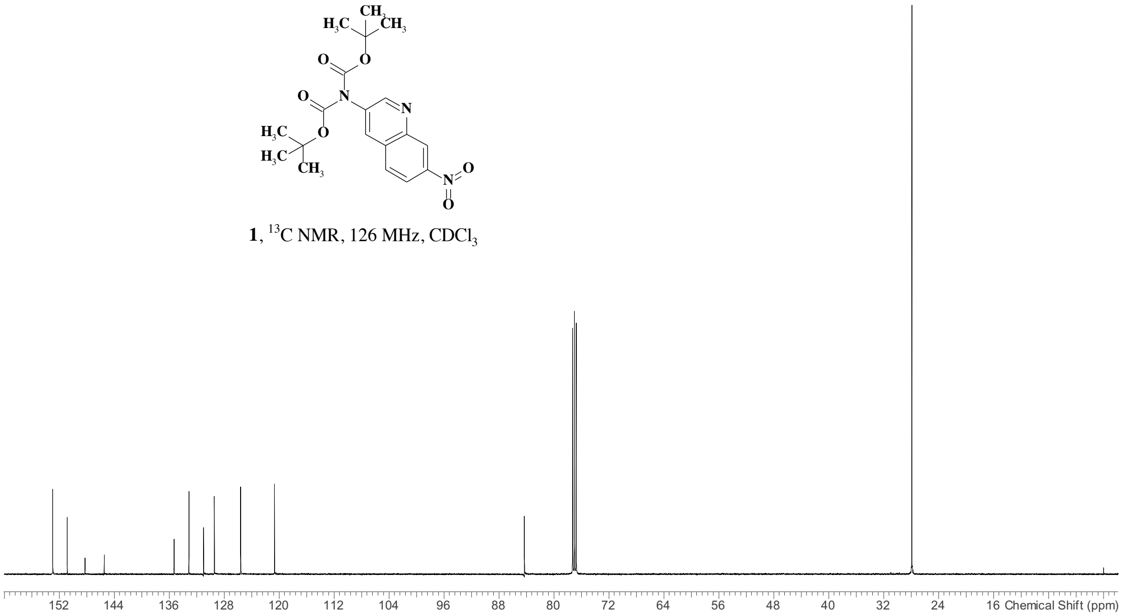

1, ^{13}C NMR, 126 MHz, $CDCl_3$

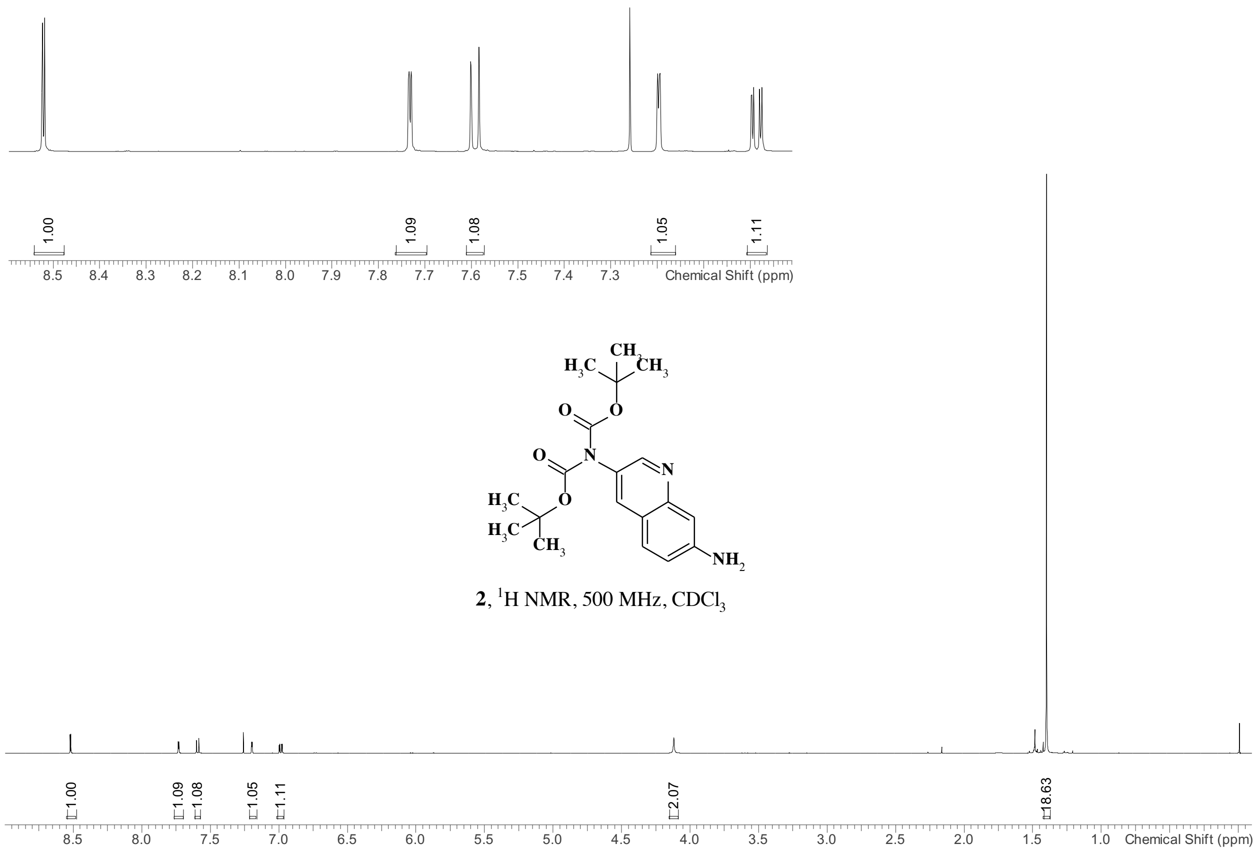

2, ^{1}H NMR, 500 MHz, $CDCl_3$

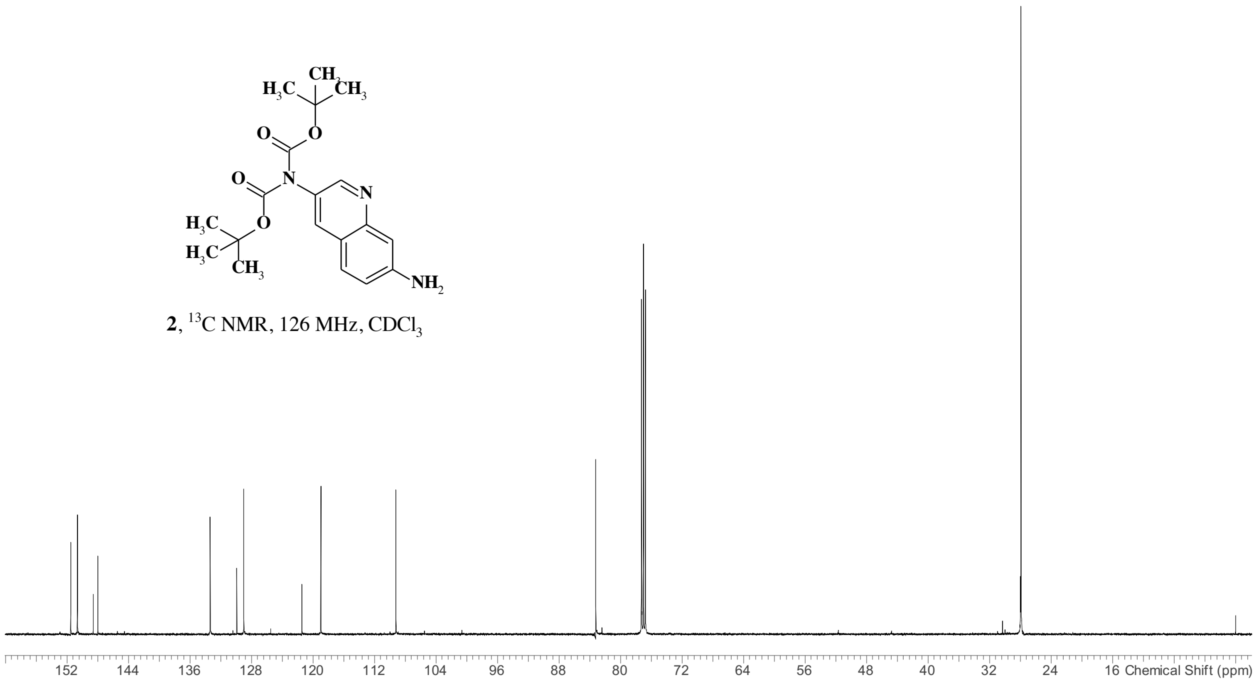

2, ^{13}C NMR, 126 MHz, $CDCl_3$

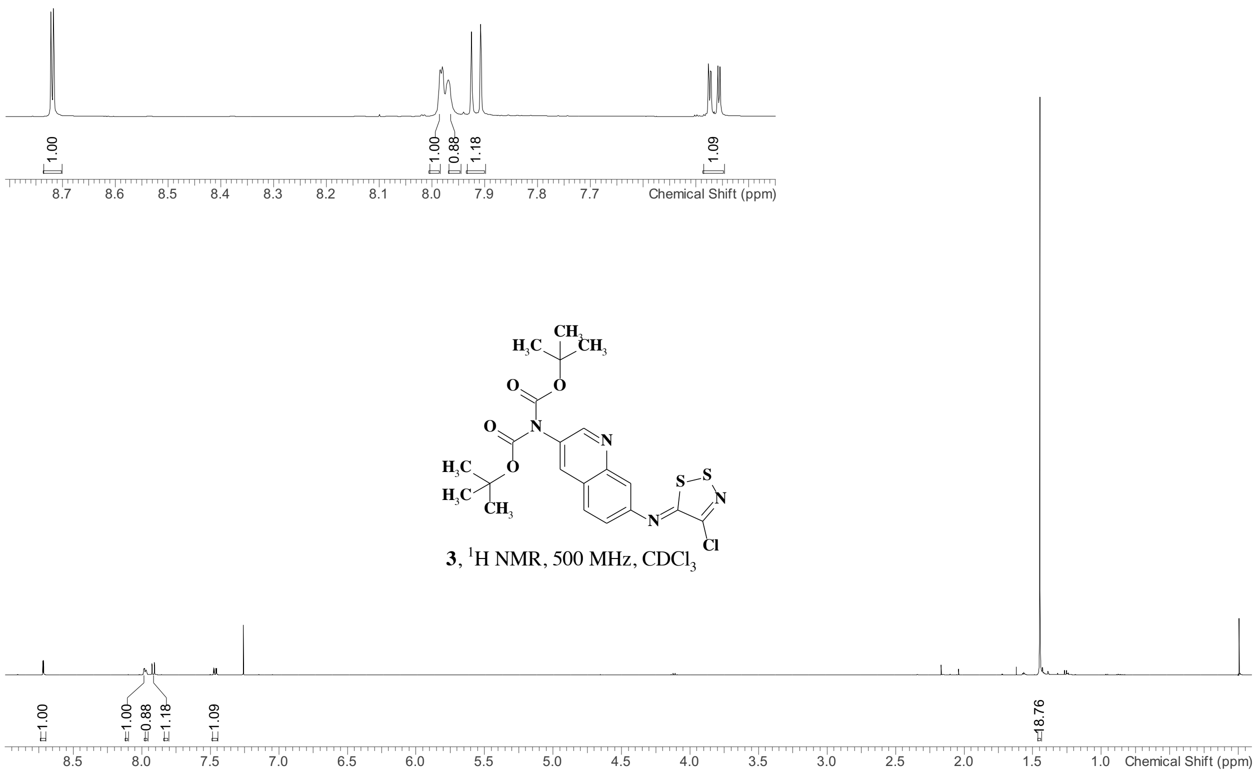

3, ^{1}H NMR, 500 MHz, $CDCl_3$

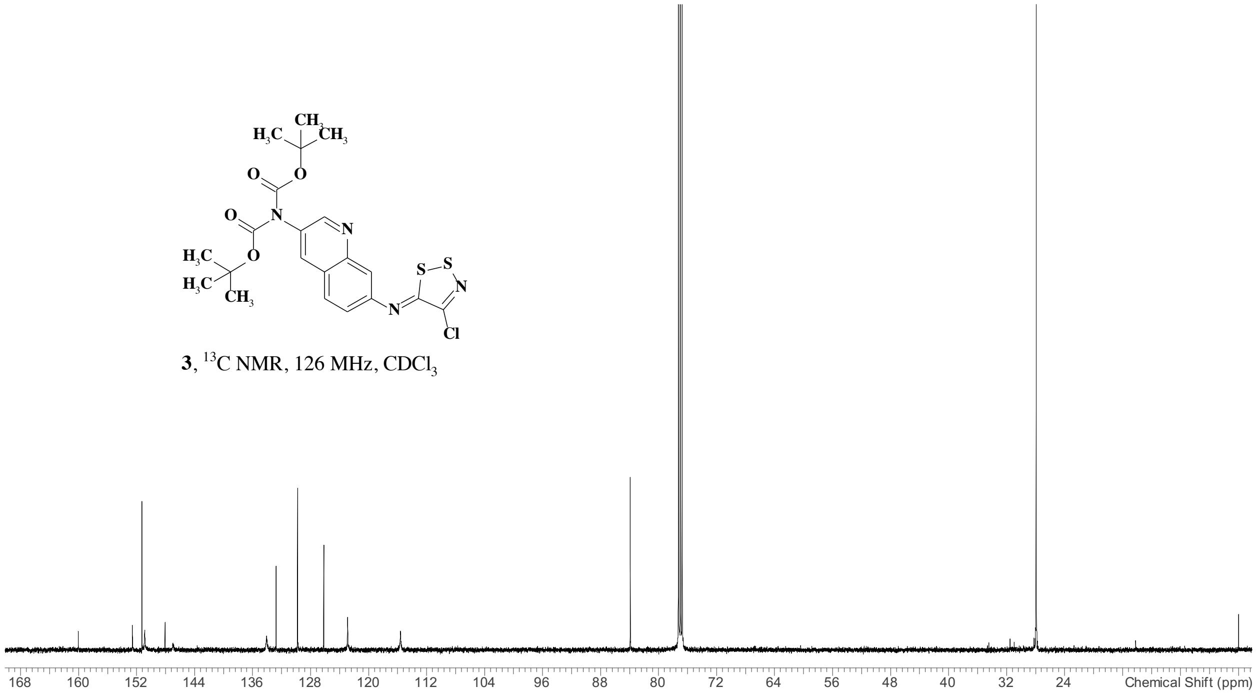

3, ^{13}C NMR, 126 MHz, $CDCl_3$

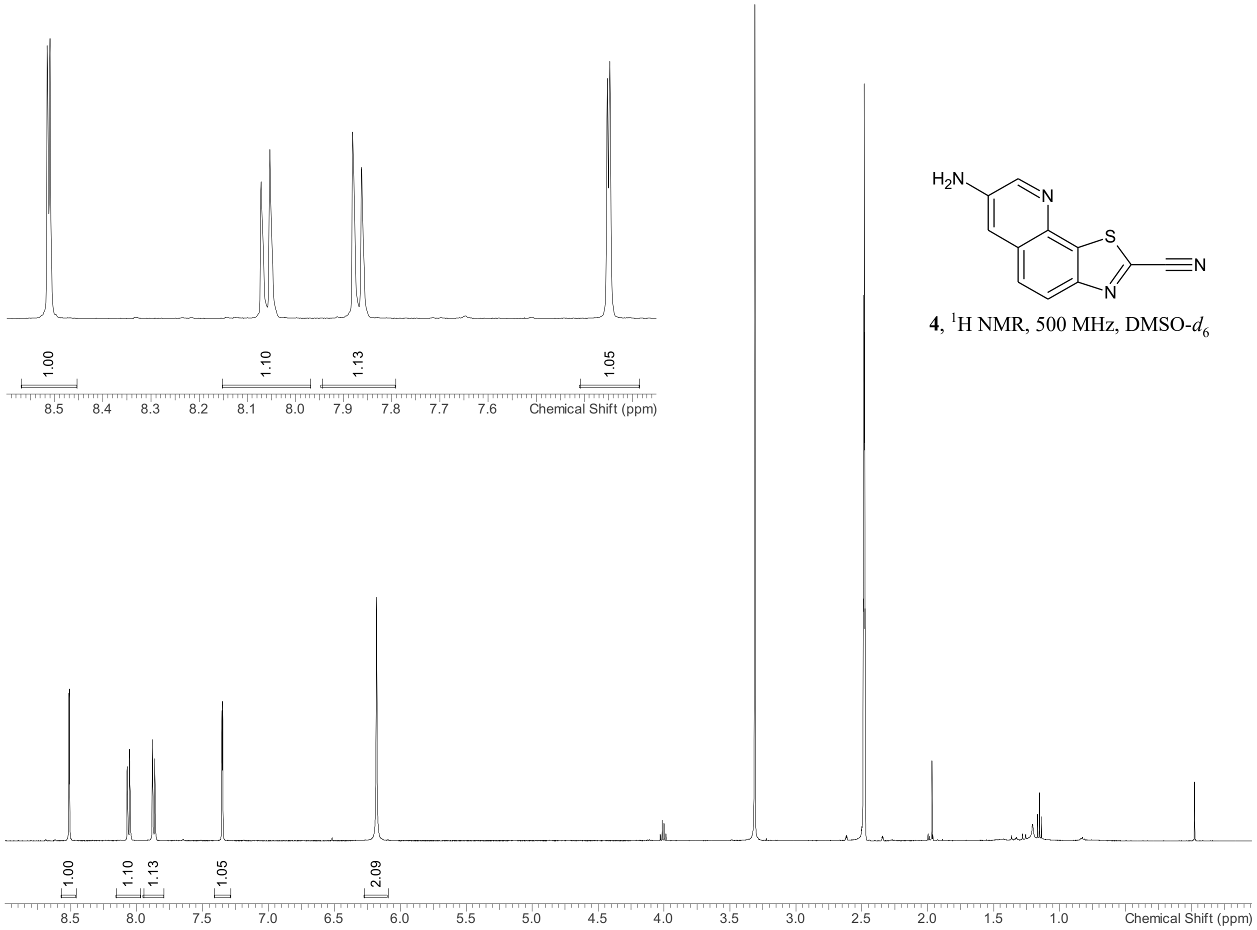

4, ^{1}H NMR, 500 MHz, DMSO-d_6

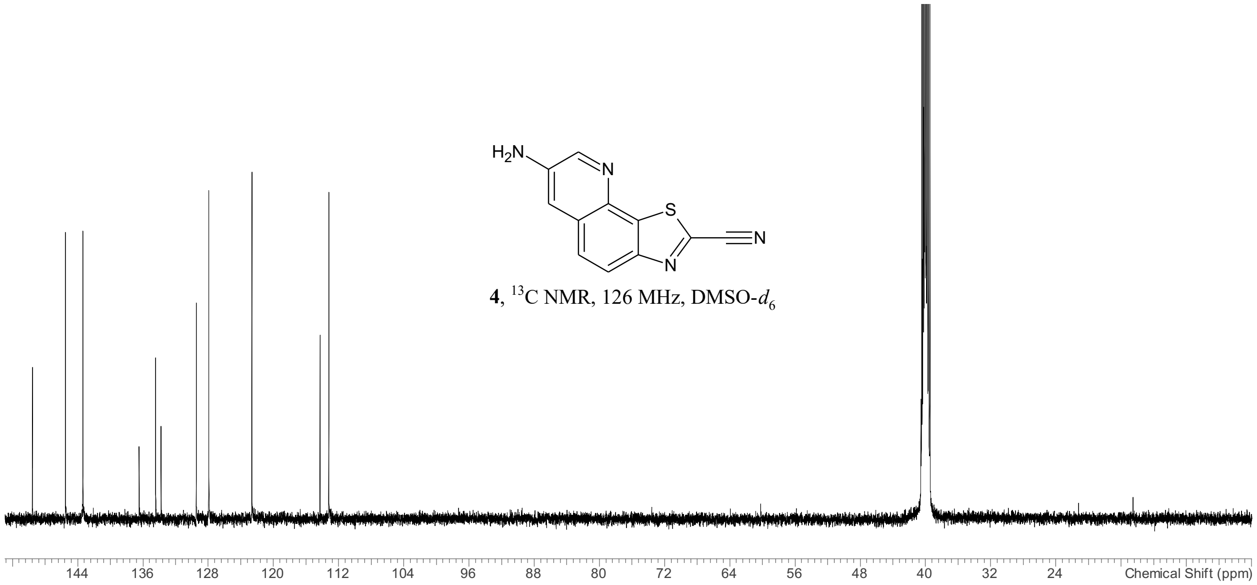

4, ^{13}C NMR, 126 MHz, DMSO-d_6

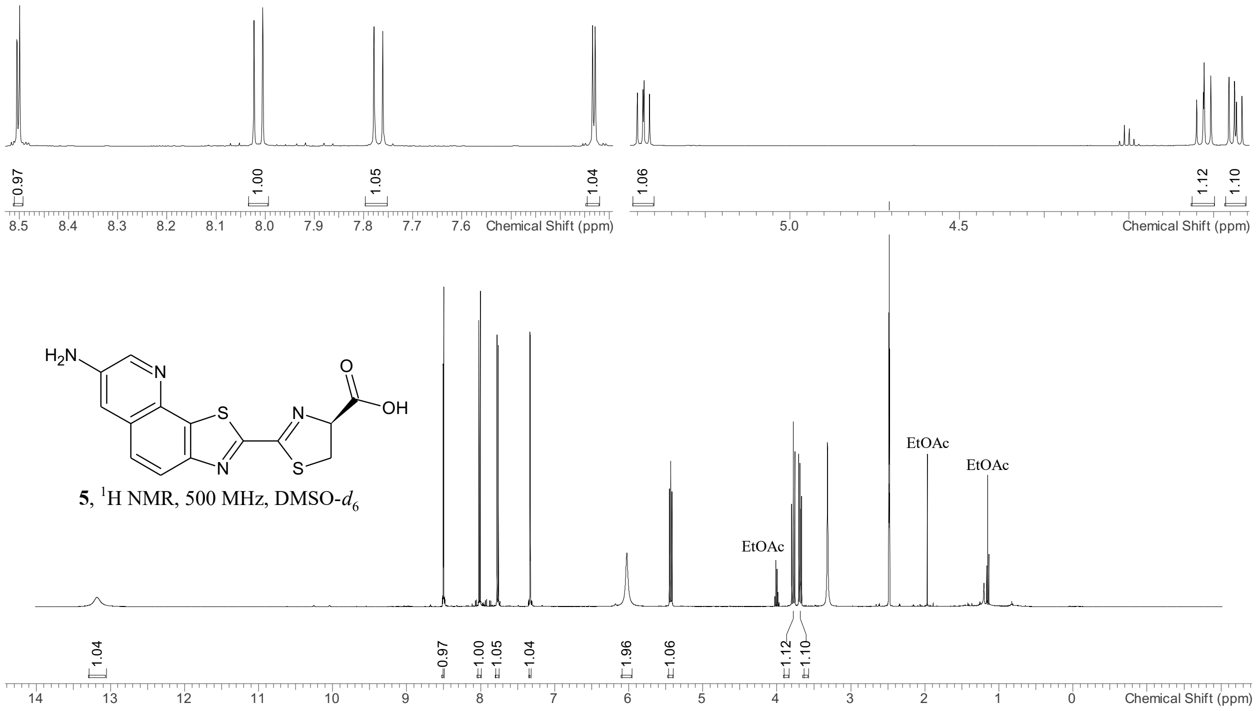

5, ^{1}H NMR, 500 MHz, DMSO-d_6

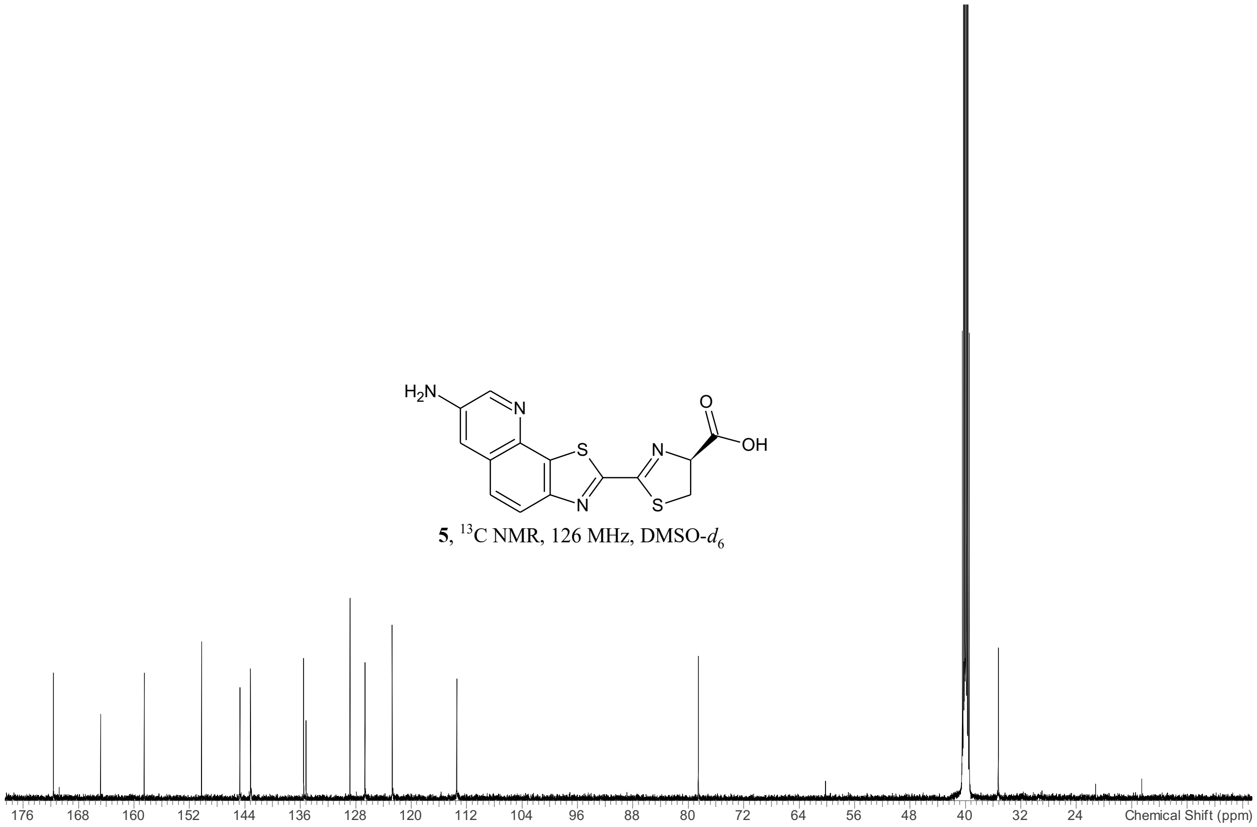

5, ^{13}C NMR, 126 MHz, DMSO-d_6

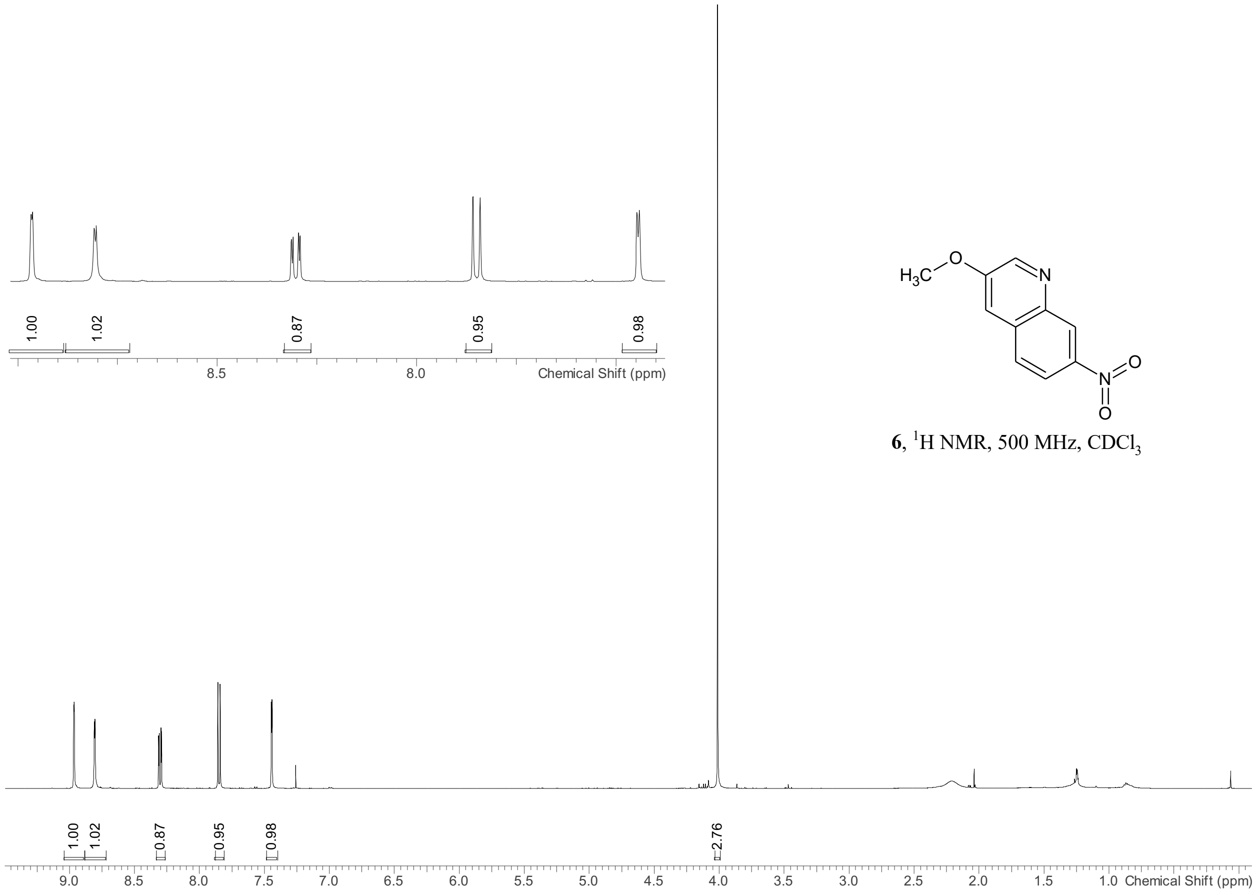

6, ^{1}H NMR, 500 MHz, $CDCl_3$

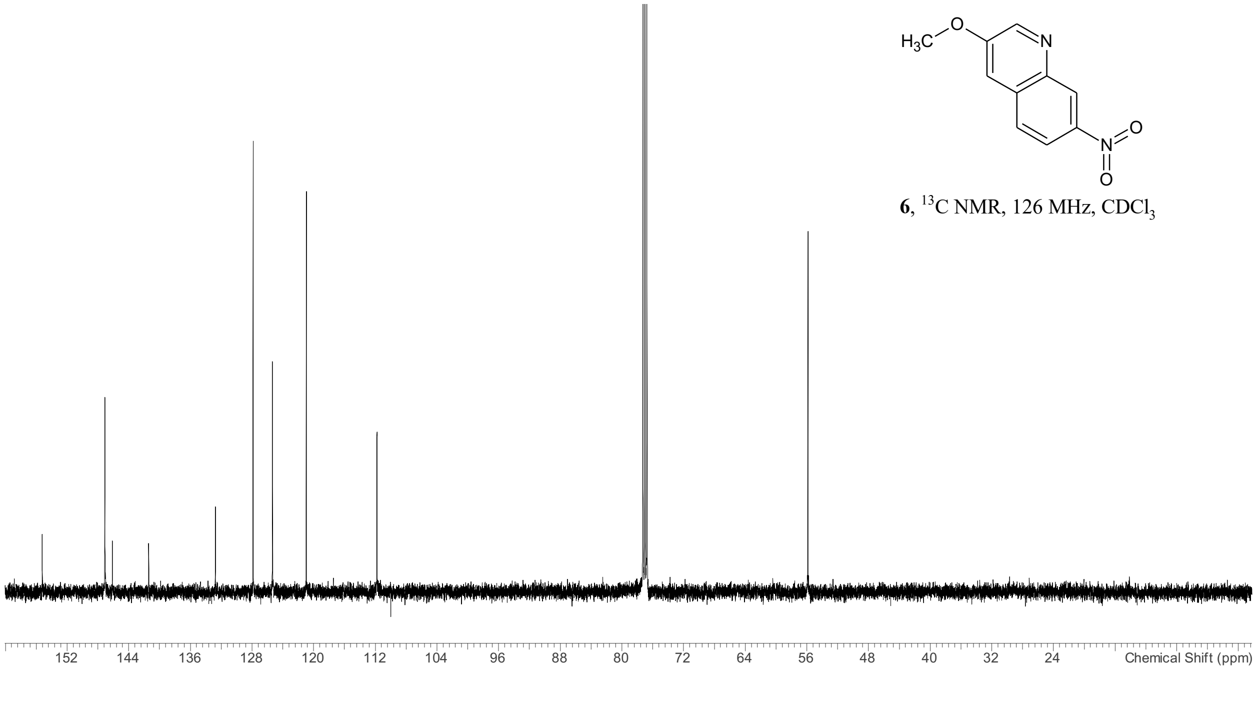

6, ^{13}C NMR, 126 MHz, $CDCl_3$

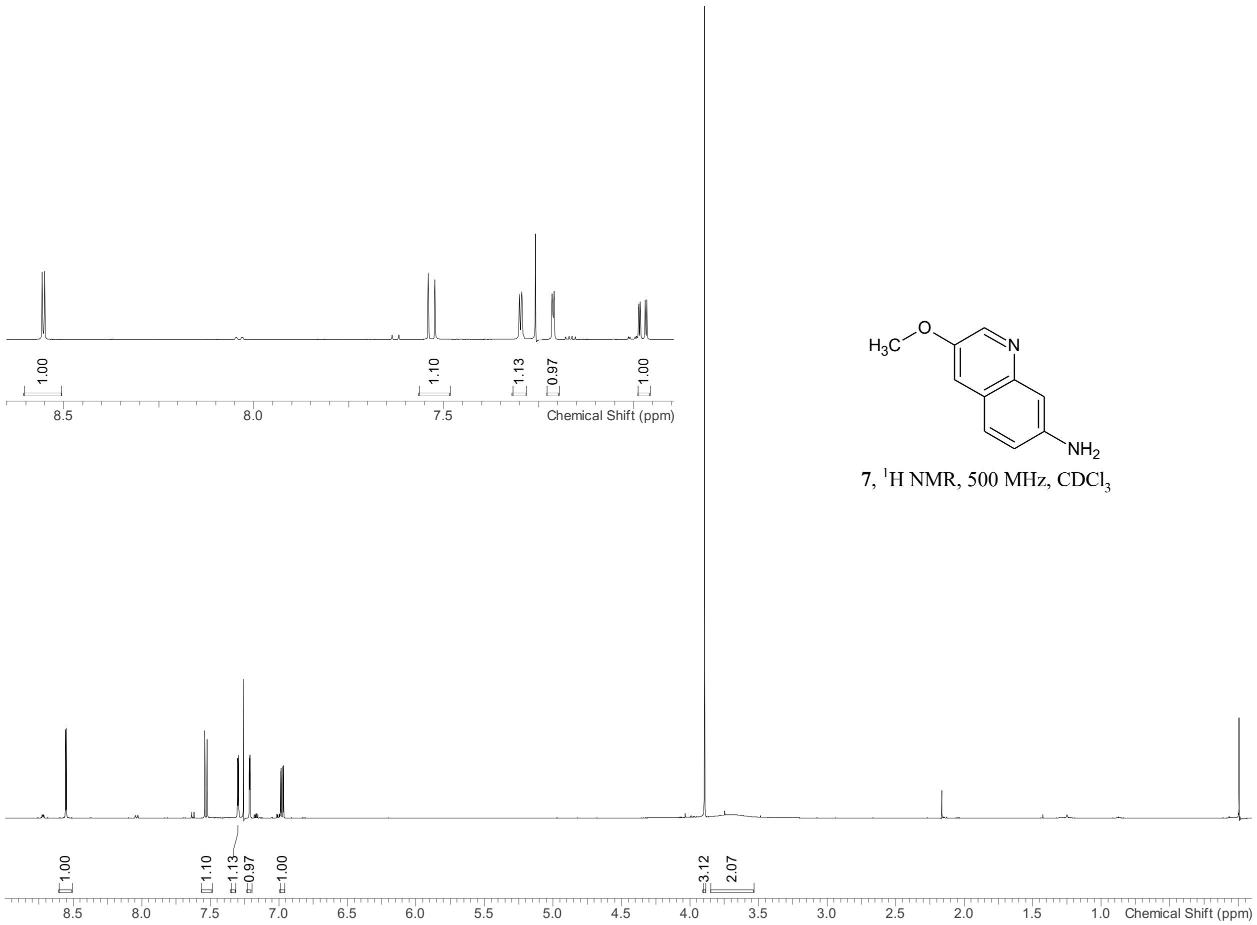

7, ^{1}H NMR, 500 MHz, $CDCl_3$

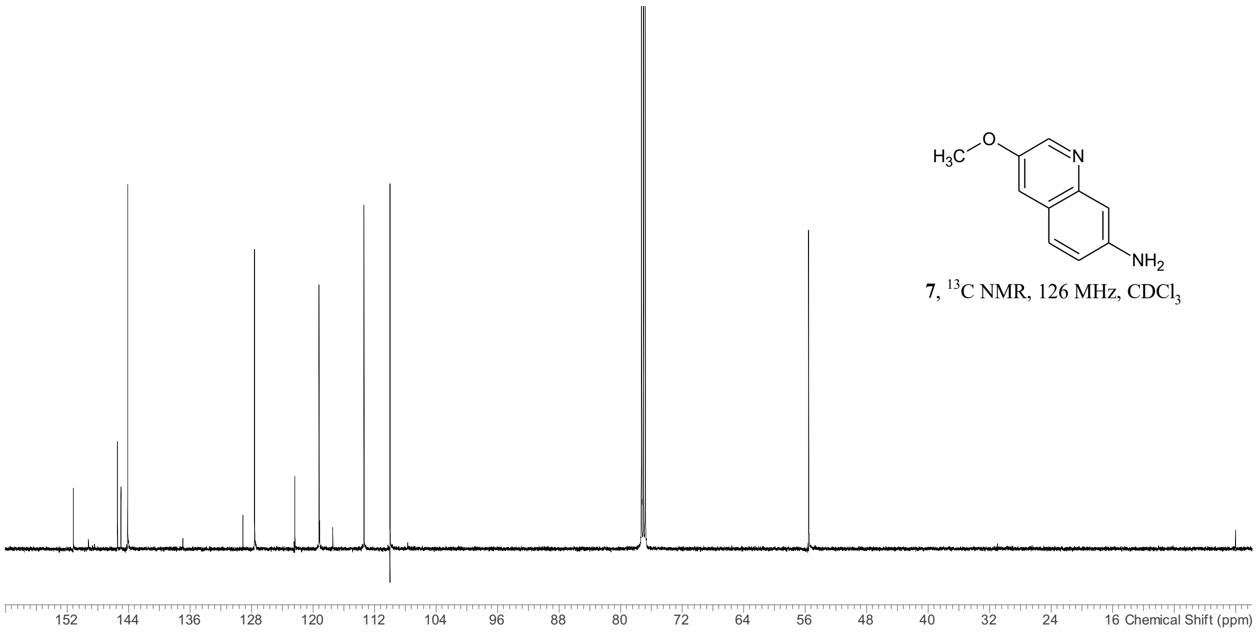

7, ^{13}C NMR, 126 MHz, $CDCl_3$

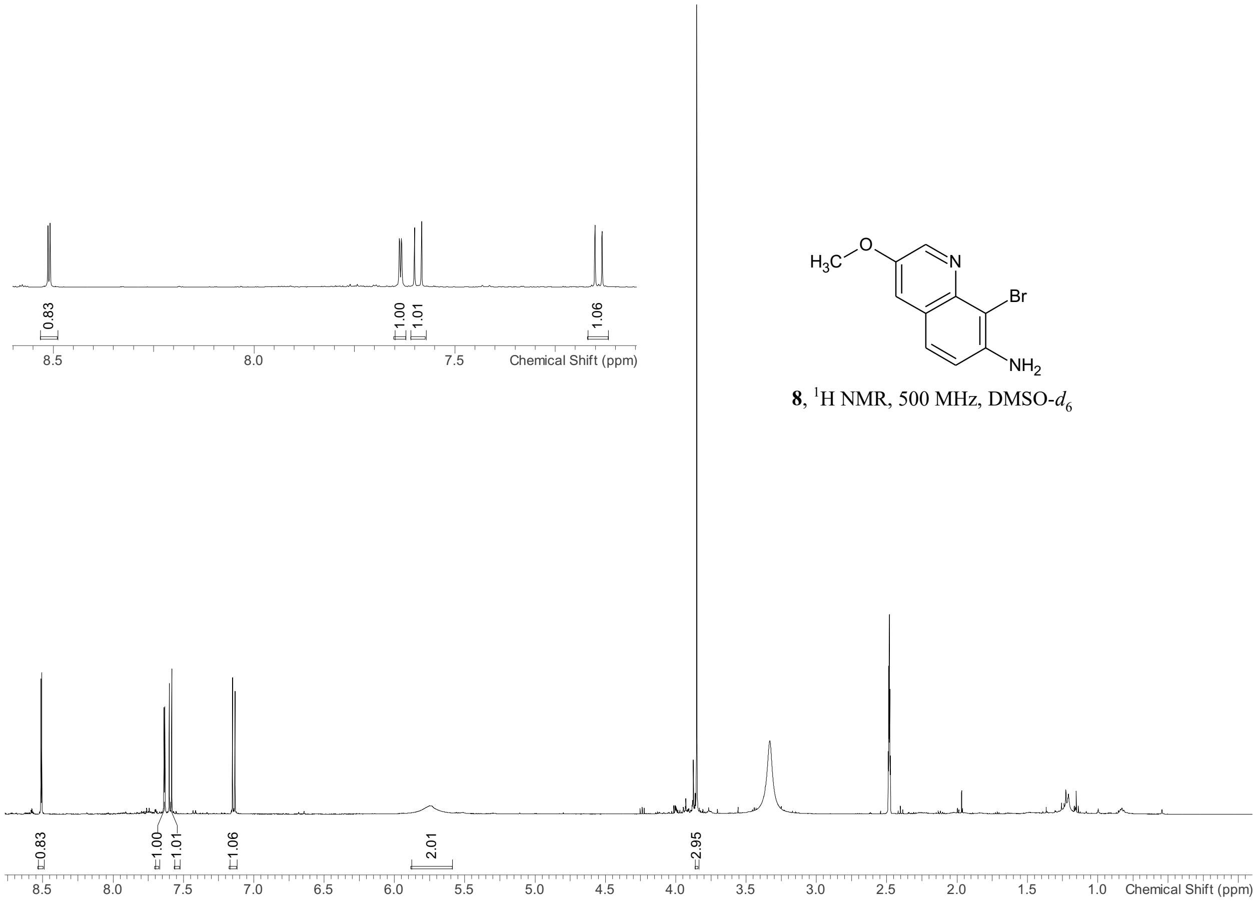

8, ^{1}H NMR, 500 MHz, DMSO-d_6

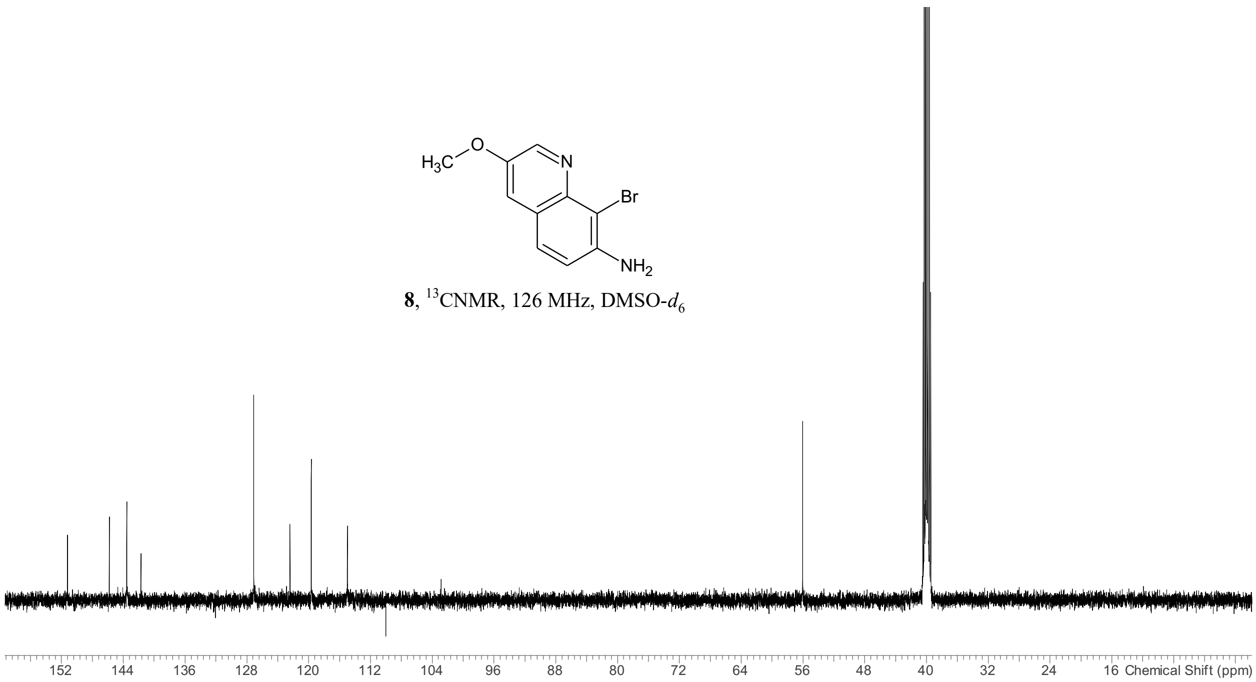

8, ^{13}CNMR, 126 MHz, DMSO-d_6

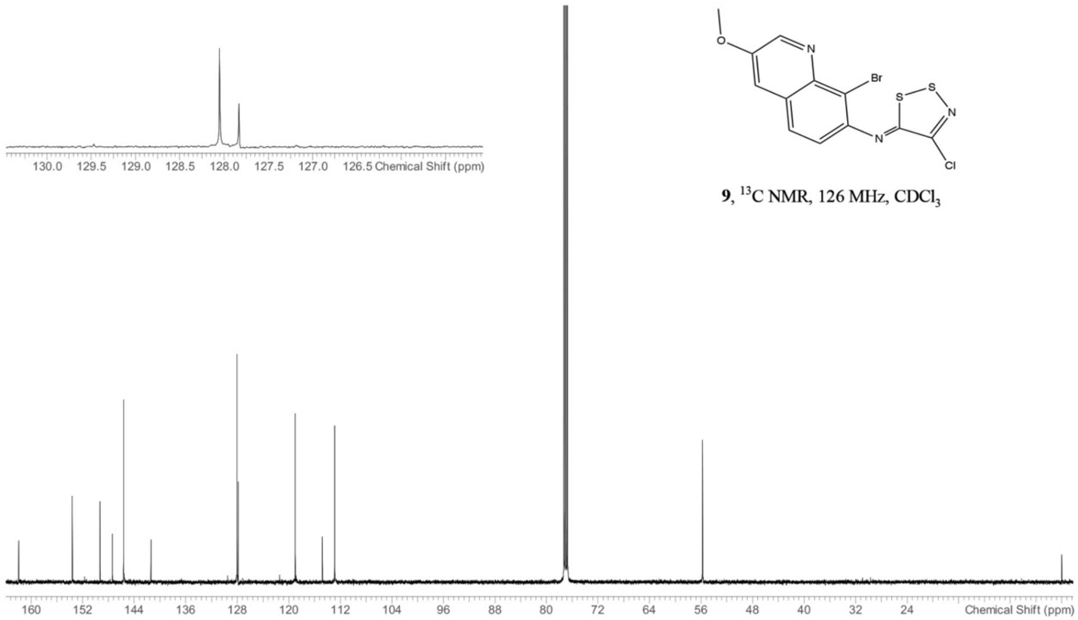
Br
S
S
N
N
Cl
130.0 129.5 129.0 128.5 128.0 127.5 127.0 126.5 Chemical Shift (ppm)
9, ^{13}C NMR, 126 MHz, $CDCl_3$
160 152 144 136 128 120 112 104 96 88 80 72 64 56 48 40 32 24 Chemical Shift (ppm)

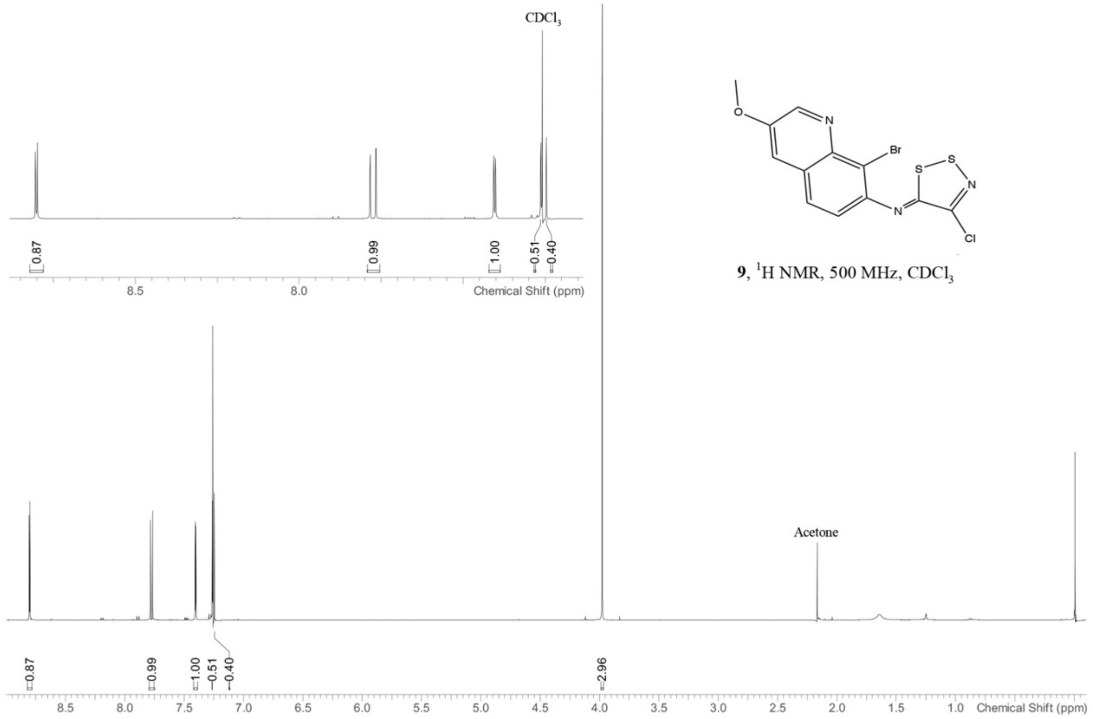
$CDCl_3$
Br
S
S
N
N
Cl
9, ^{1}H NMR, 500 MHz, $CDCl_3$
0.87
0.99
1.00
0.51
0.40
8.5 8.0 Chemical Shift (ppm)
Acetone
2.96
8.5 8.0 7.5 7.0 6.5 6.0 5.5 5.0 4.5 4.0 3.5 3.0 2.5 2.0 1.5 1.0 Chemical Shift (ppm)

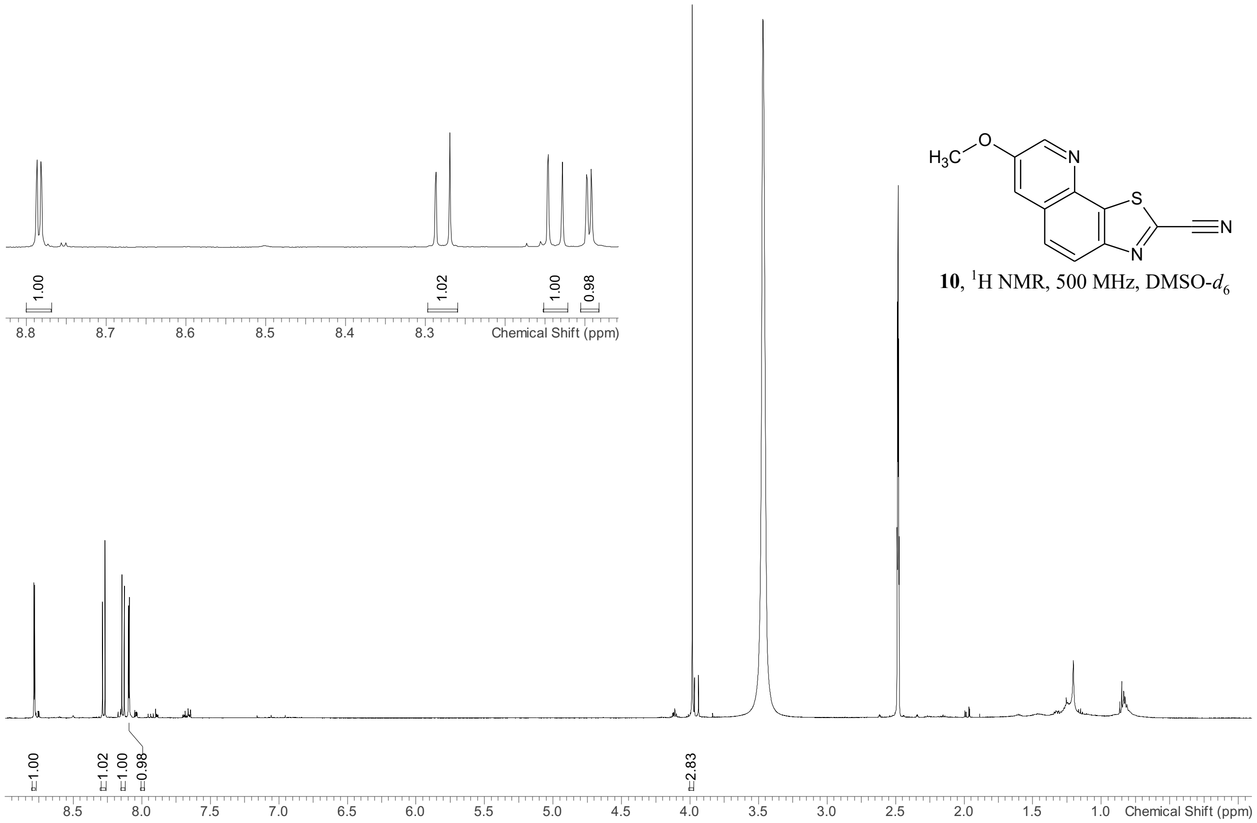

10, ^{1}H NMR, 500 MHz, DMSO-d_6

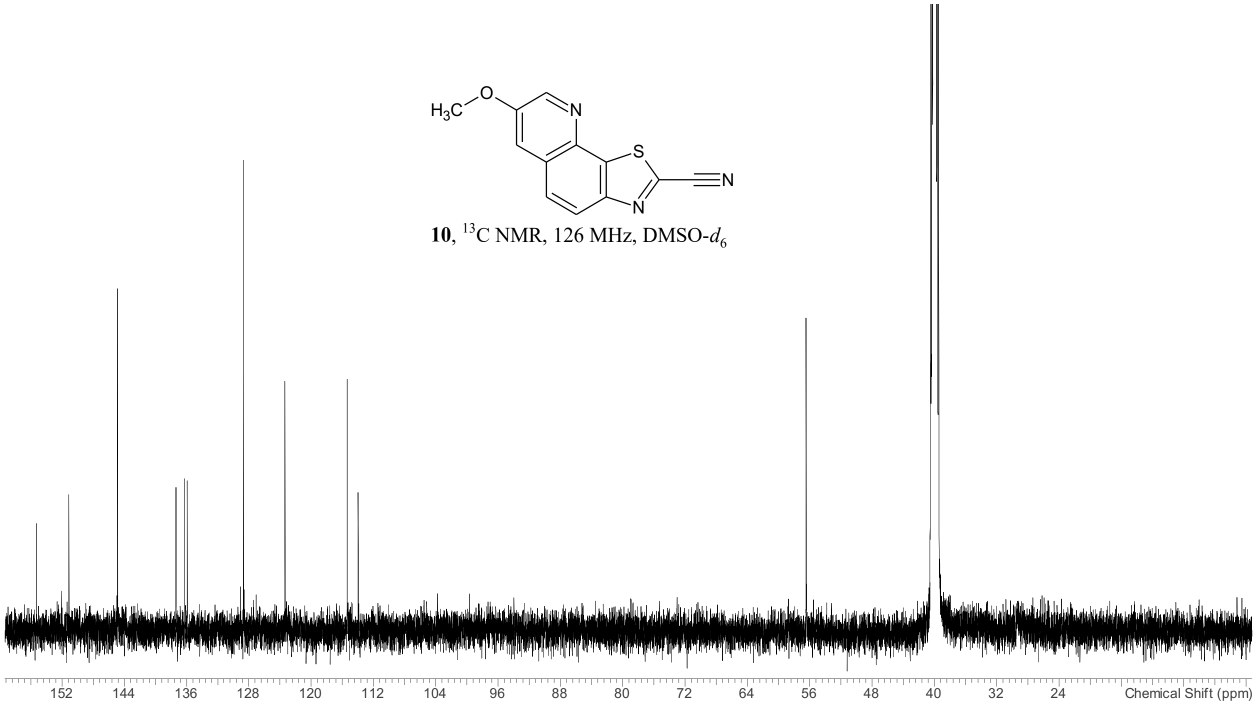

10, ^{13}C NMR, 126 MHz, DMSO-d_6

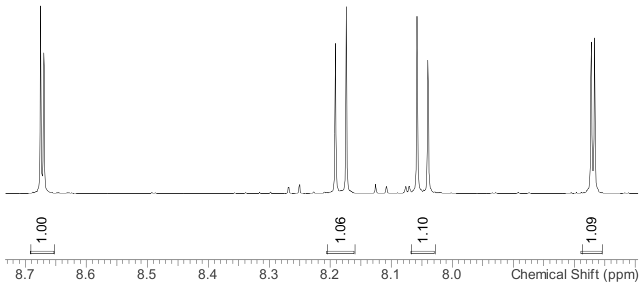

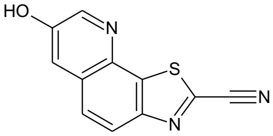

11, ^{1}H NMR, 500 MHz, DMSO-d_6

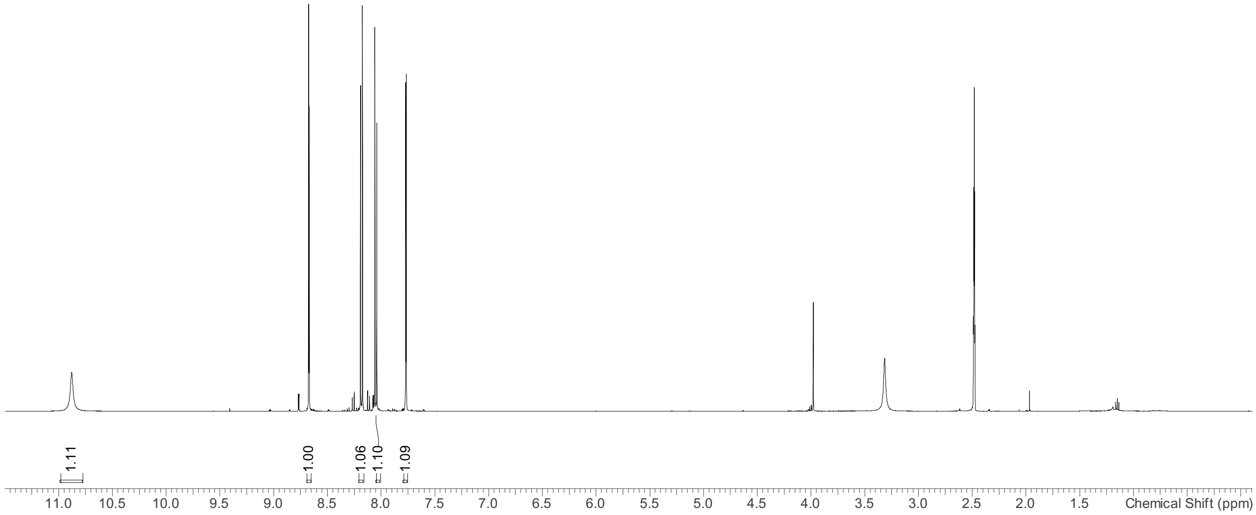

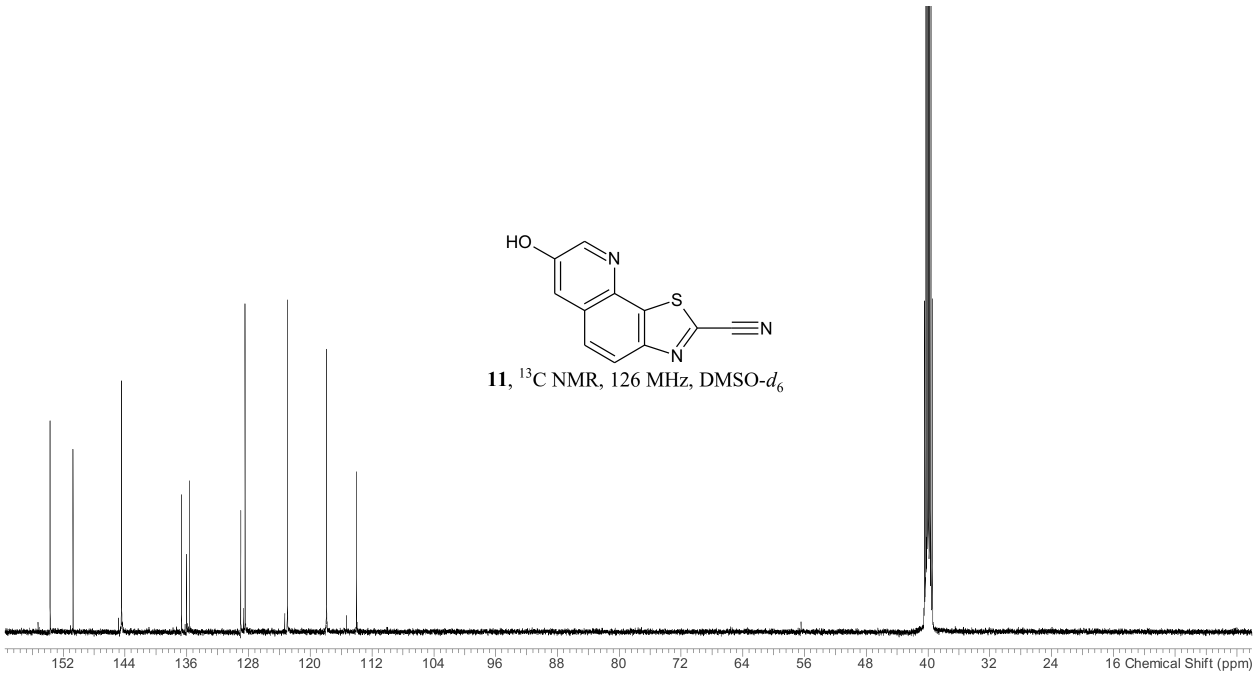

11, ^{13}C NMR, 126 MHz, DMSO-d_6

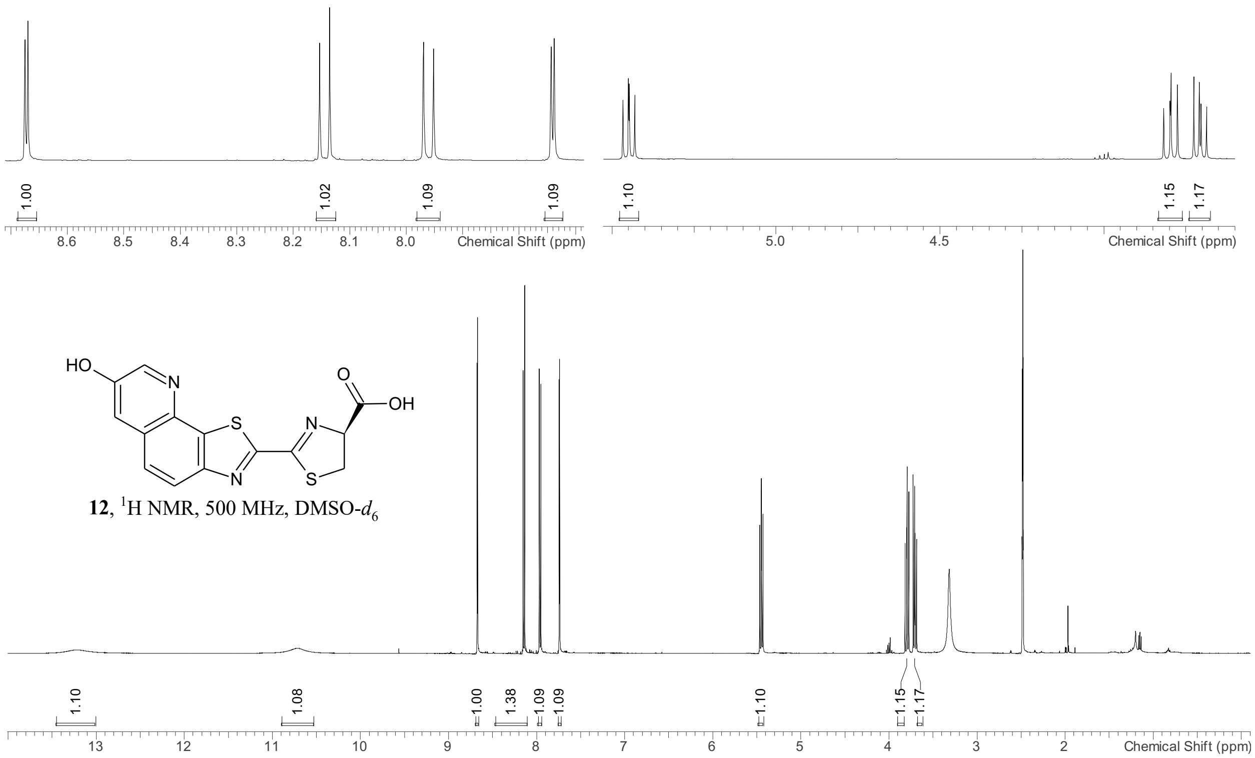

12, ^{1}H NMR, 500 MHz, DMSO-d_6

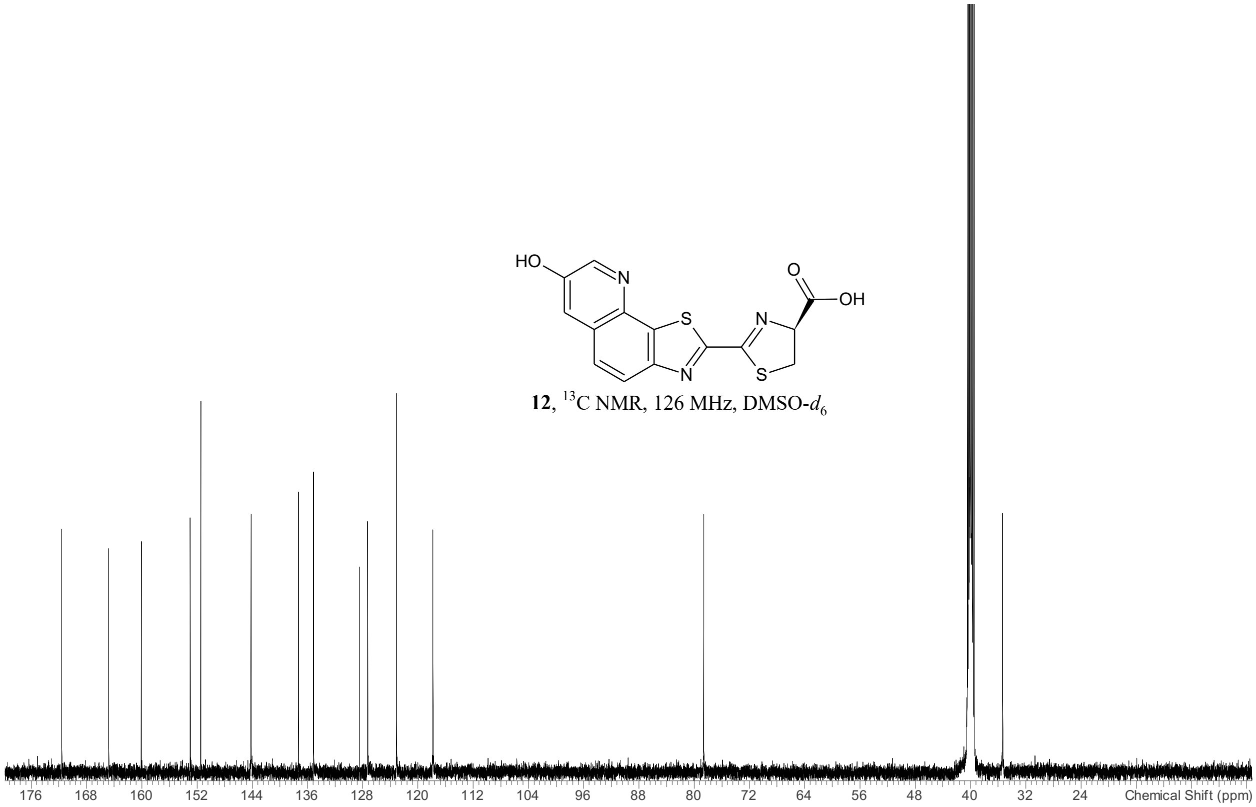

12, ^{13}C NMR, 126 MHz, DMSO-d_6

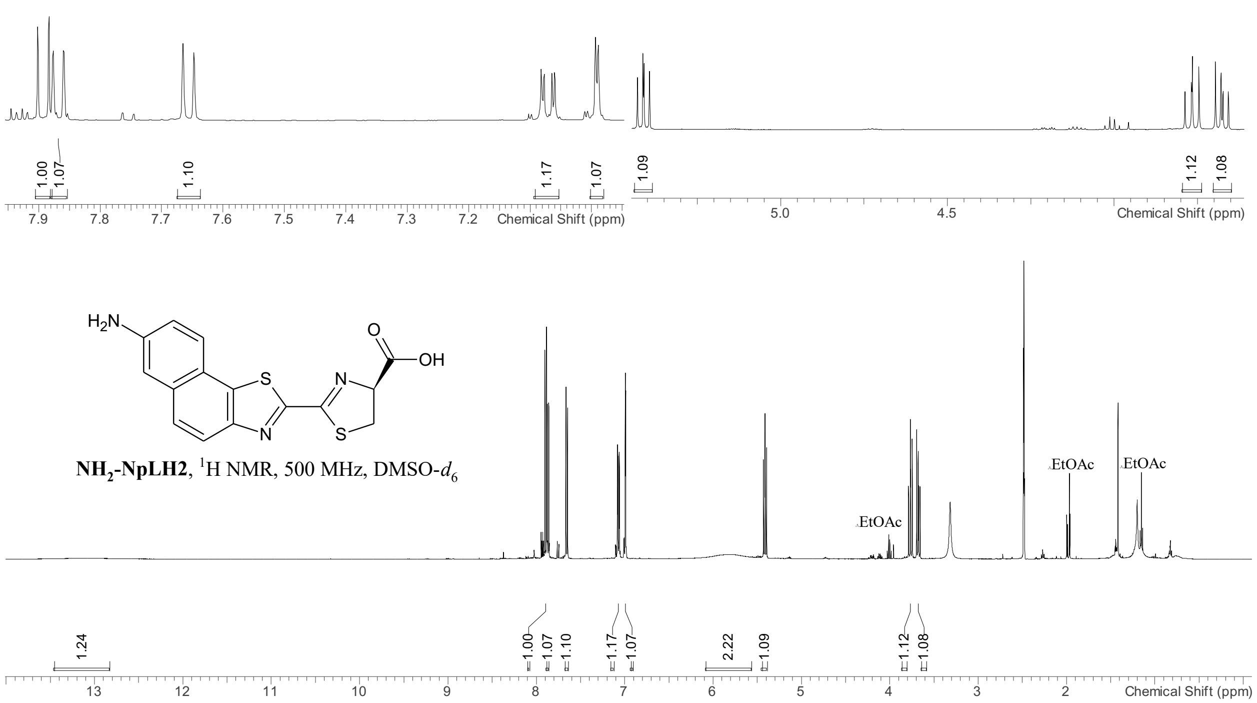

NH_2-NpLH2, ^{1}H NMR, 500 MHz, DMSO-d_6

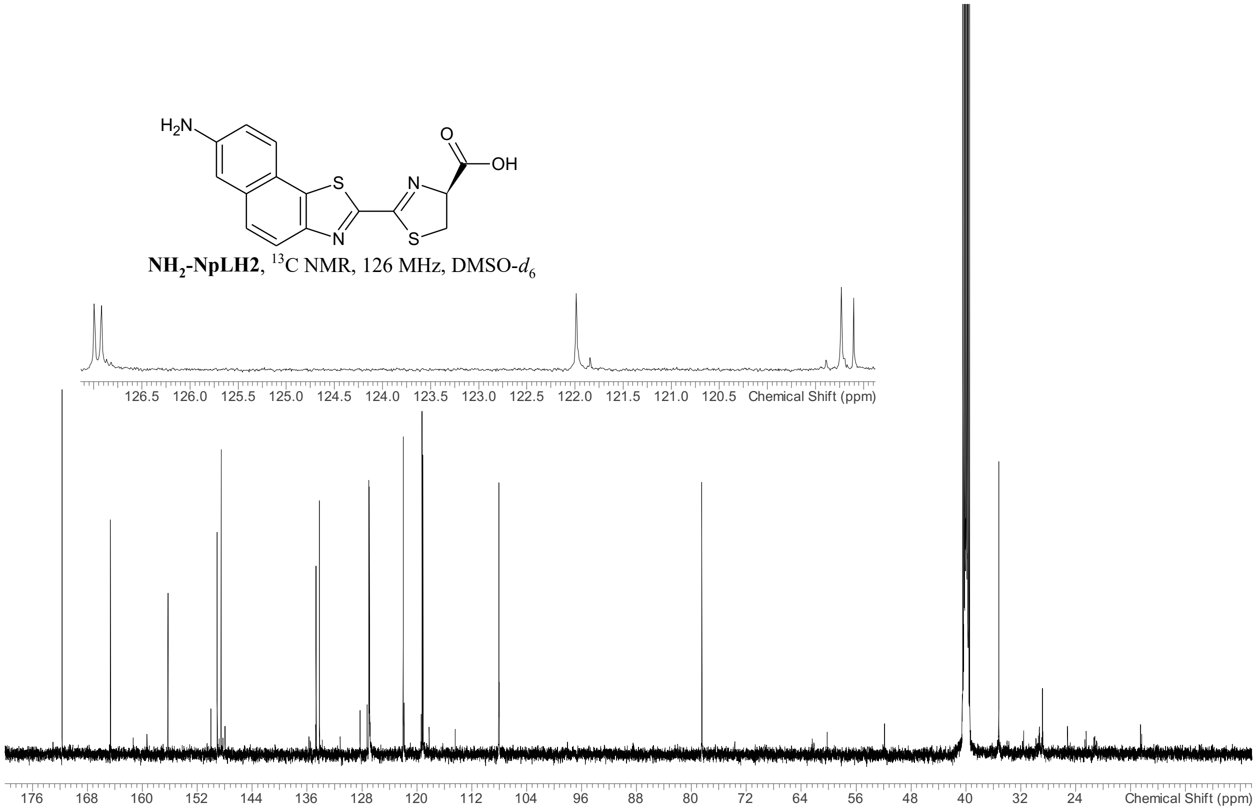

NH_2-NpLH2, ^{13}C NMR, 126 MHz, DMSO-d_6

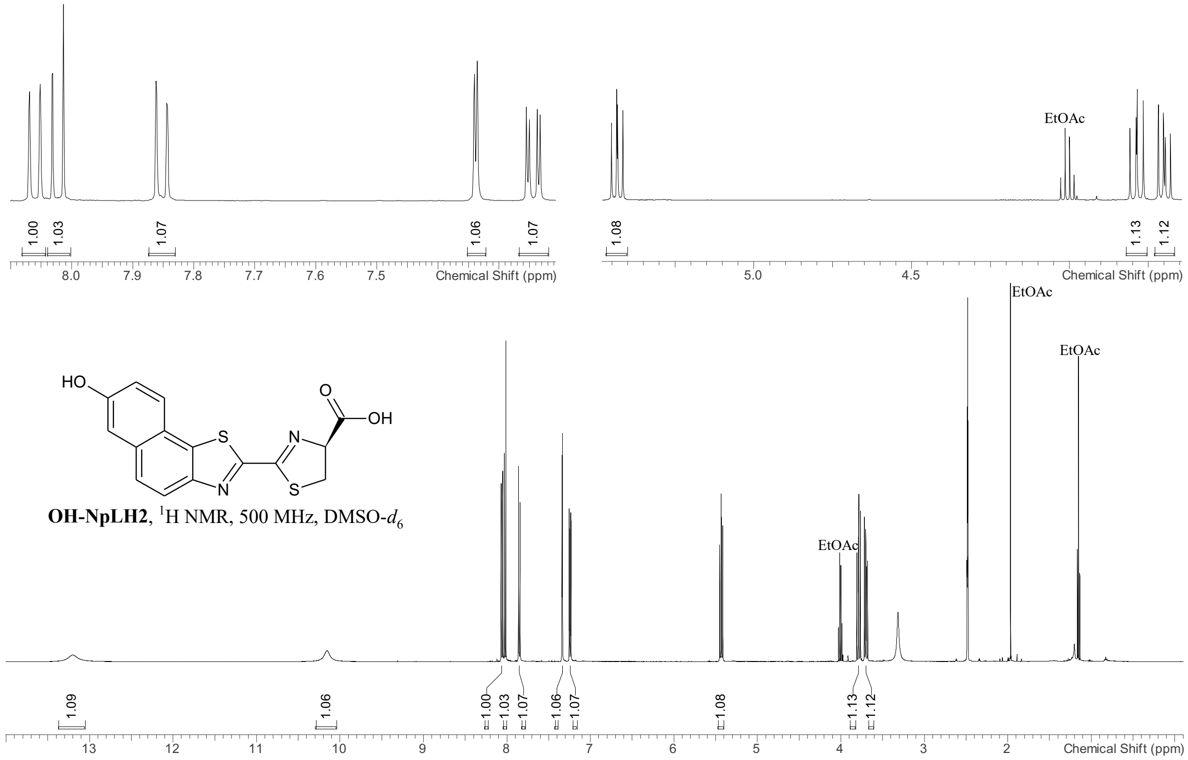

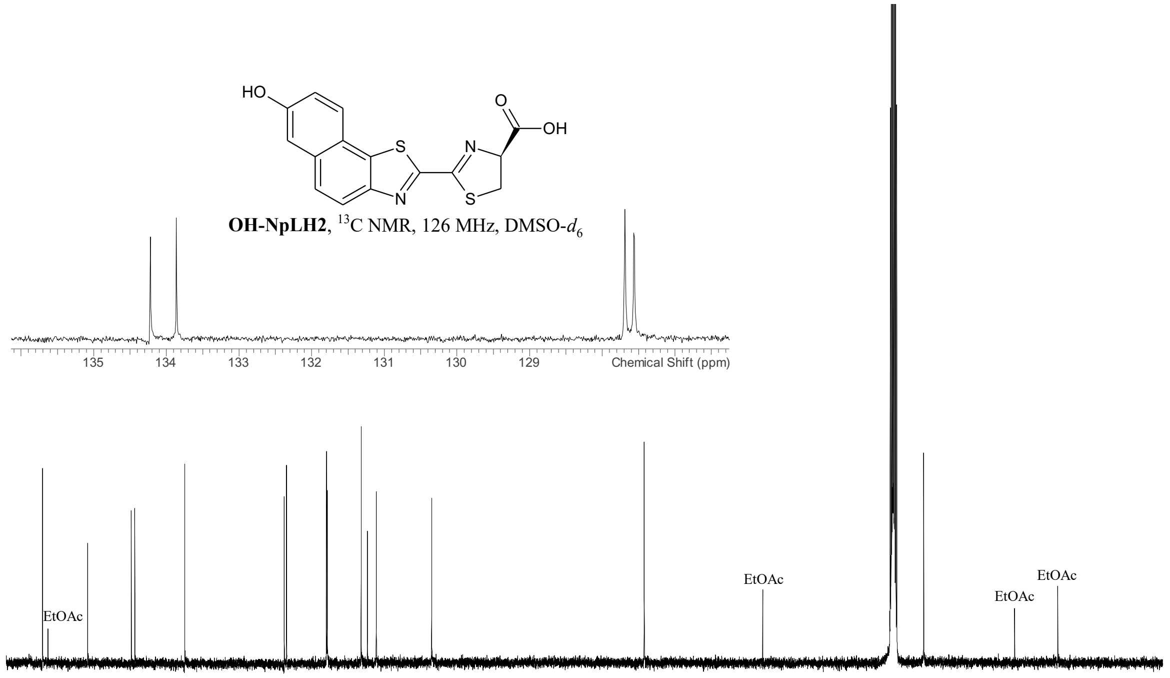

Figure S5. NMR spectra of NH_2-QLH_2, OH-QLH_2, the synthetic intermediates leading to the quinoline-containing luciferins, NH_2-NpLH2, and OH-$NpLH_2$.

VI. IR SPECTRA

A

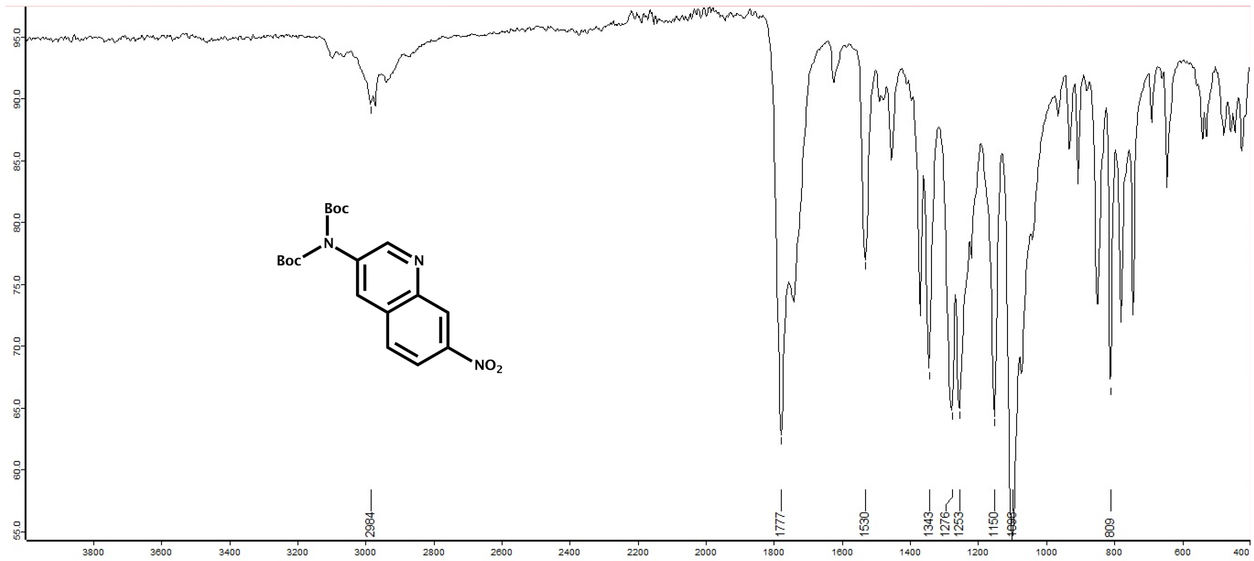

B

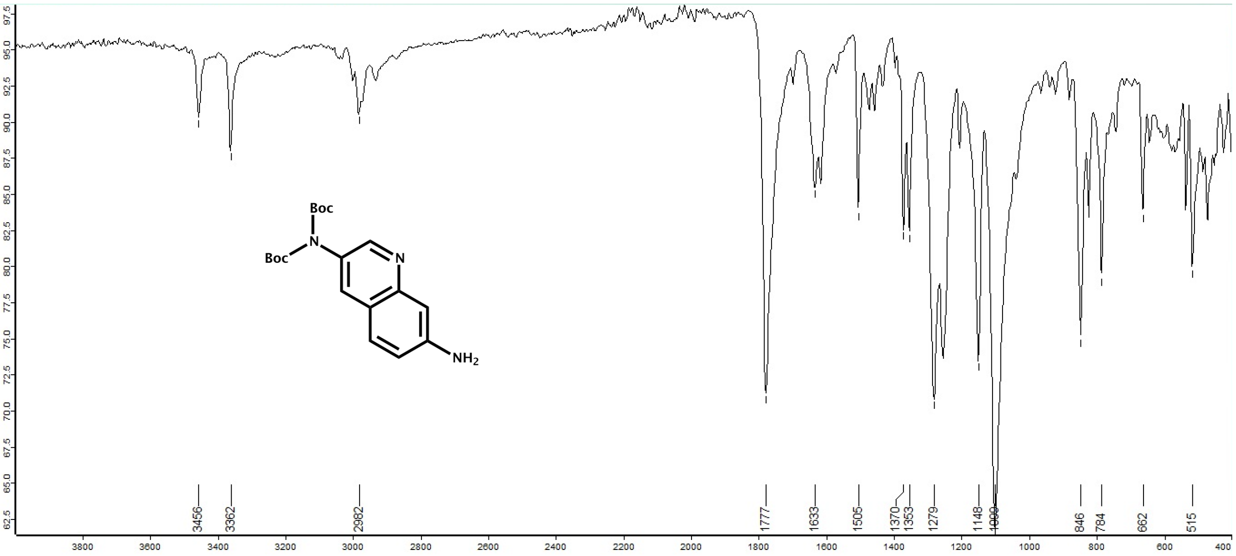

C

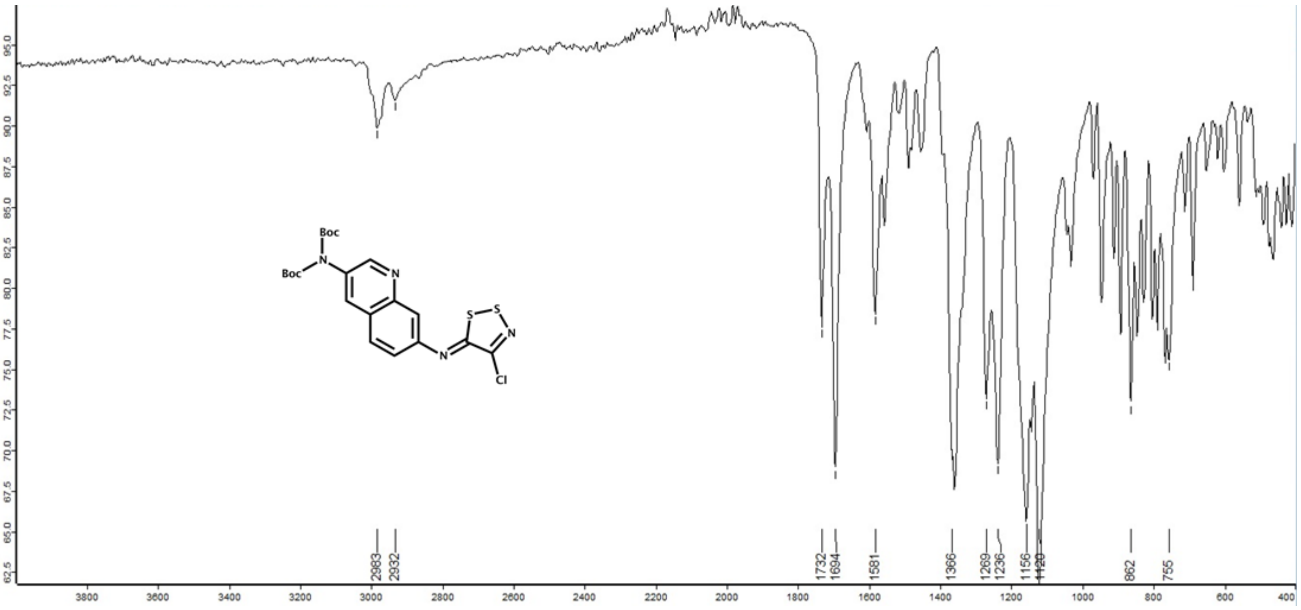
Boc
Boc
N
N
S
S
N
N
Cl
2983
2932
1732
1694
1581
1366
1269
1236
1156
1120
862
755

D

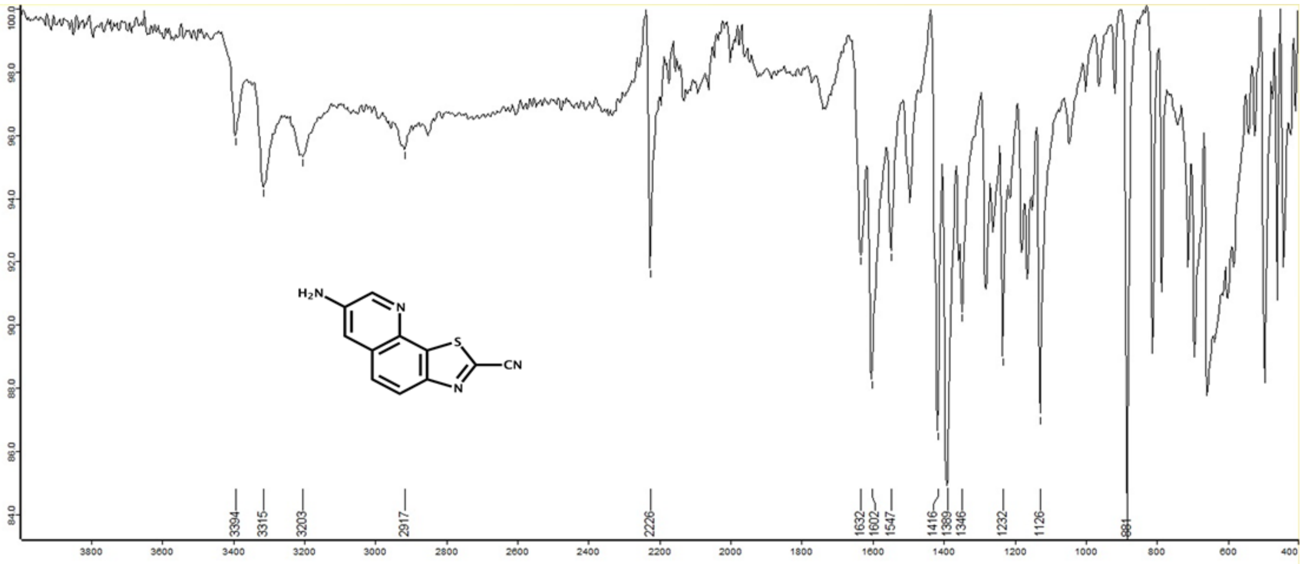
H_2N
N
S
N
CN
3394
3315
3203
2917
2226
1632
1602
1547
1416
1389
1346
1232
1126
881

E

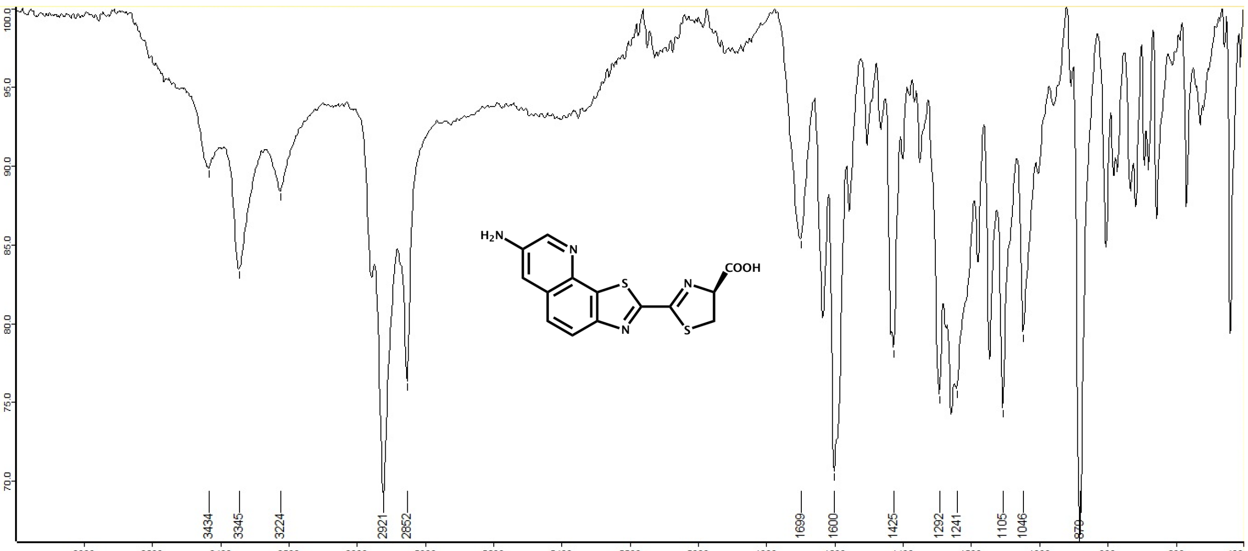

100.0
95.0
90.0
85.0
80.0
75.0
70.0
3434
3345
3224
2921
2852
1699
1600
1425
1292
1241
1105
1046
879
3800
3600
3400
3200
3000
2800
2600
2400
2200
2000
1800
1600
1400
1200
1000
800
600
400
H_2N
N
S
N
N
S
COOH

F

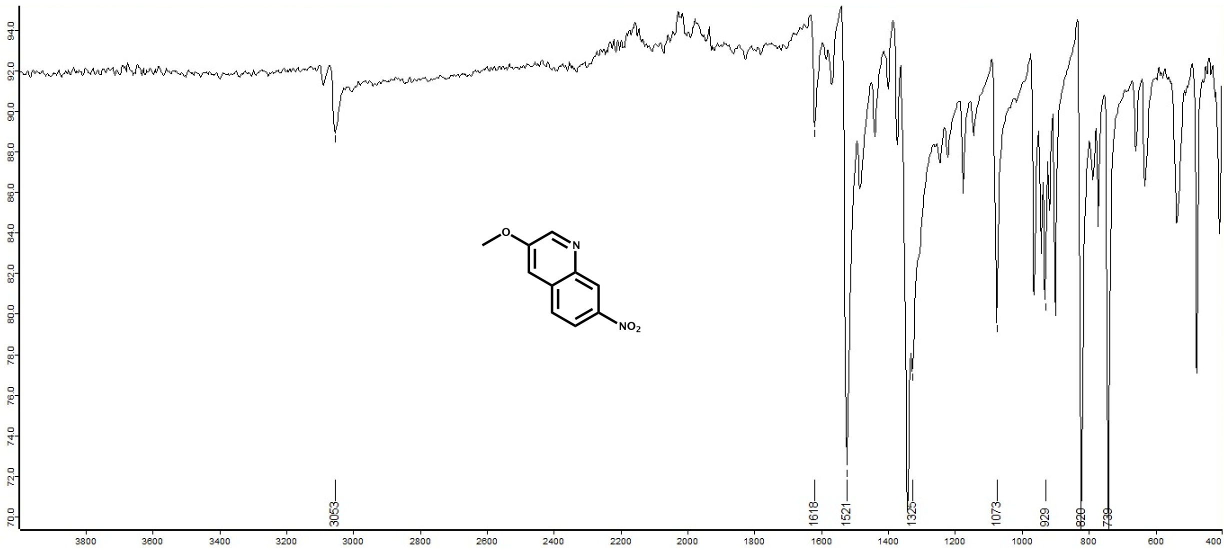

94.0
92.0
90.0
88.0
86.0
84.0
82.0
80.0
78.0
76.0
74.0
72.0
70.0
3053
1618
1521
1325
1073
929
820
739
3800
3600
3400
3200
3000
2800
2600
2400
2200
2000
1800
1600
1400
1200
1000
800
600
400
O
N
NO_2

G

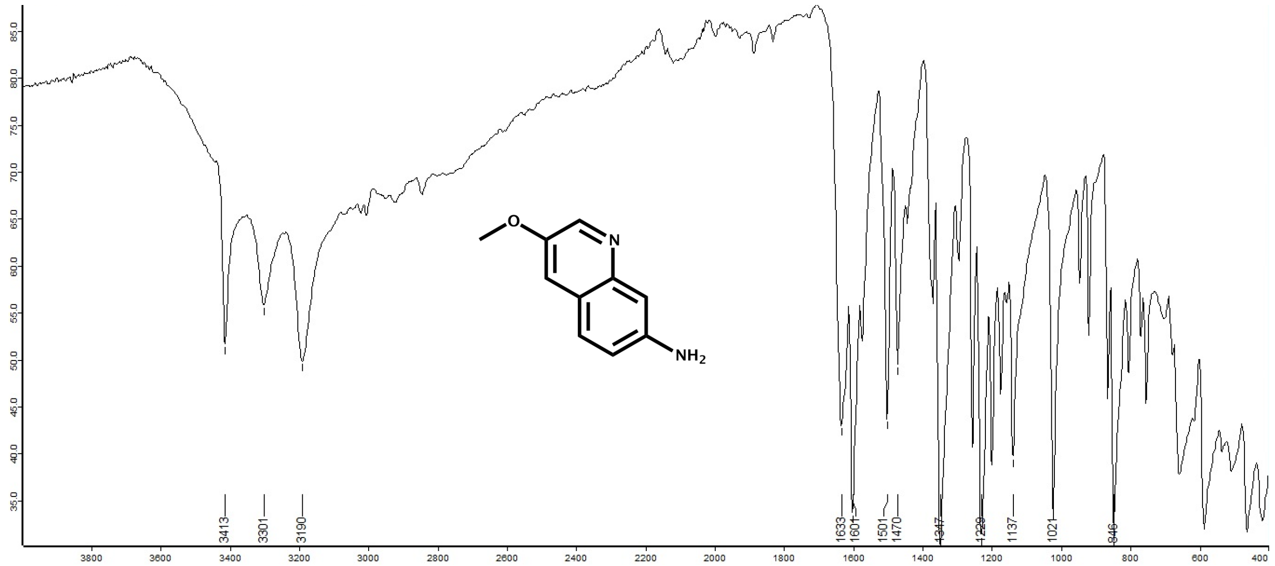

O
N
NH2
3413
3301
3190
1633
1601
1501
1470
1347
1229
1137
1021
846

H

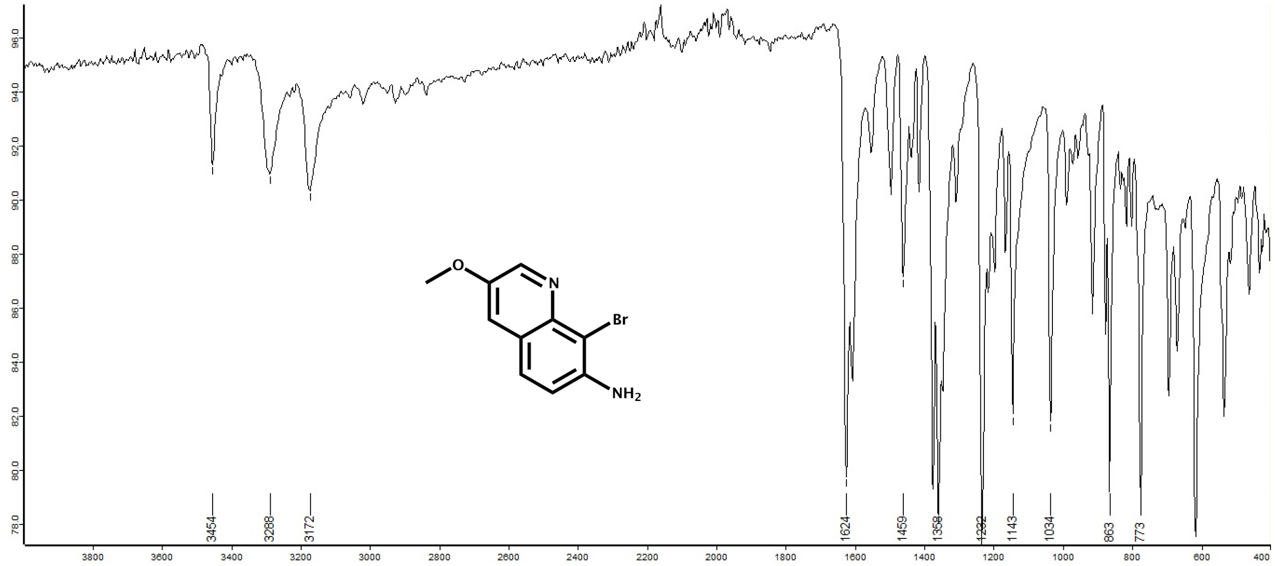

O
N
Br
NH2
3454
3288
3172
1624
1459
1358
1232
1143
1034
863
773

I

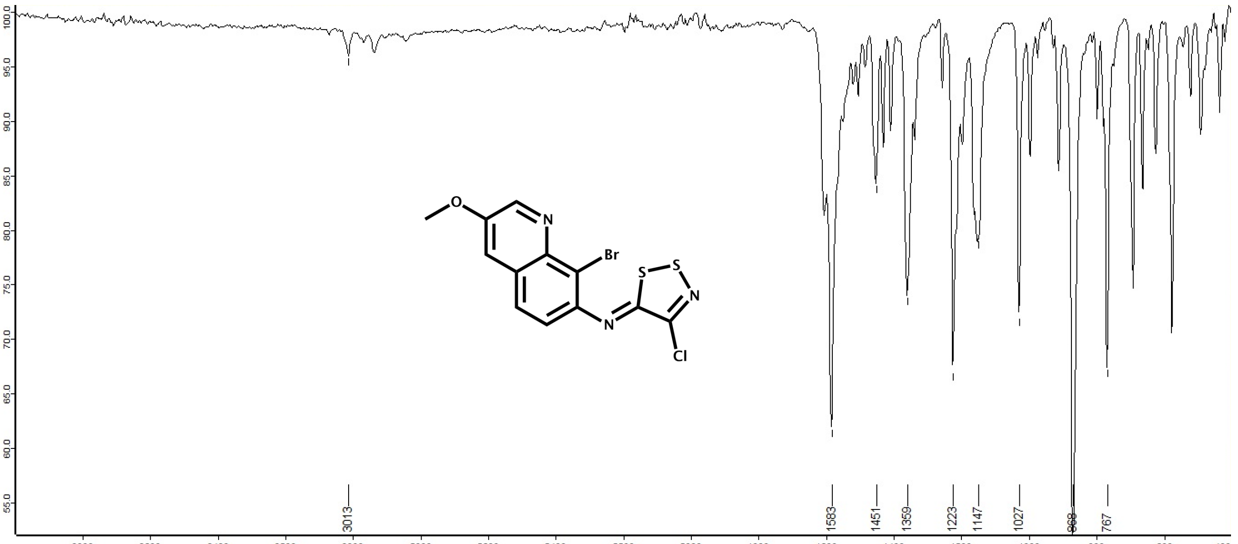
Br
S
S
N
N
Cl
O
N
3013
1583
1451
1359
1223
1147
1027
868
767

J

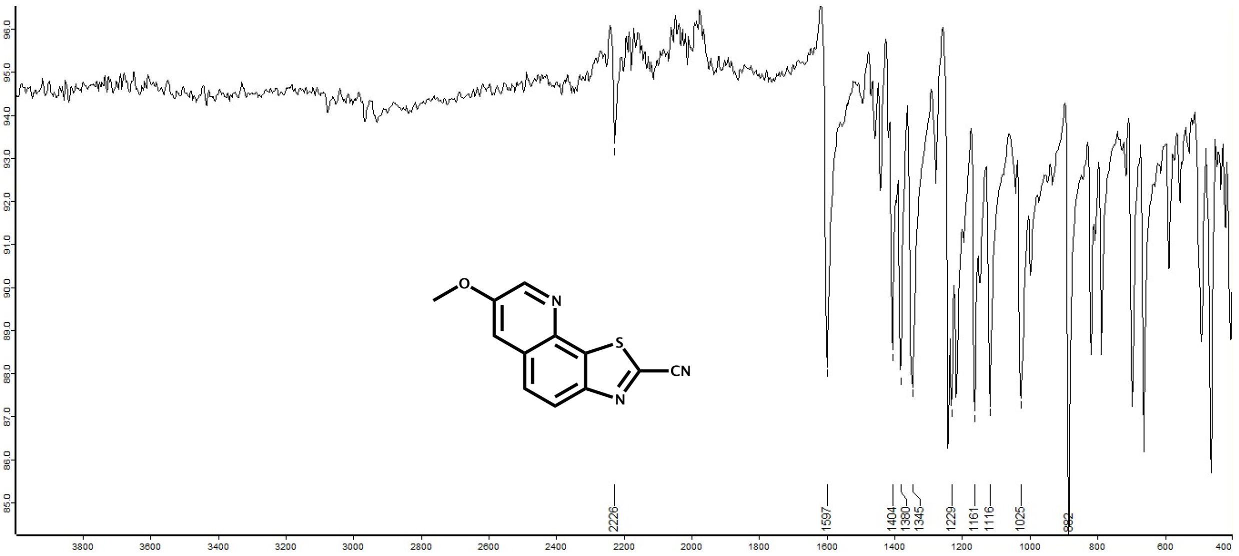
O
N
S
CN
N
2226
1597
1404
1380
1345
1229
1161
1116
1025
882

K

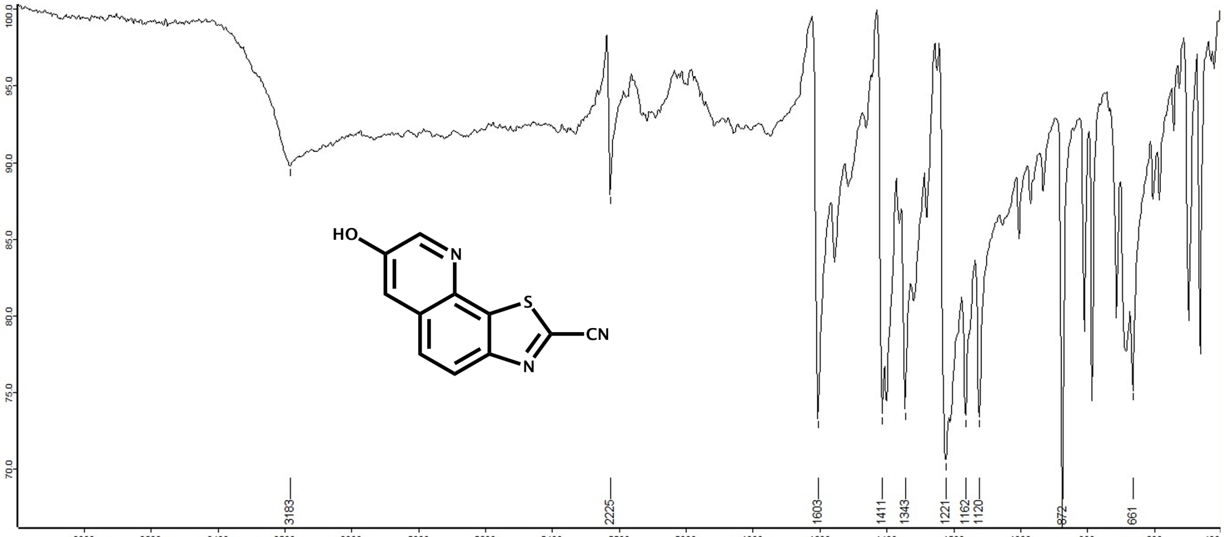

L

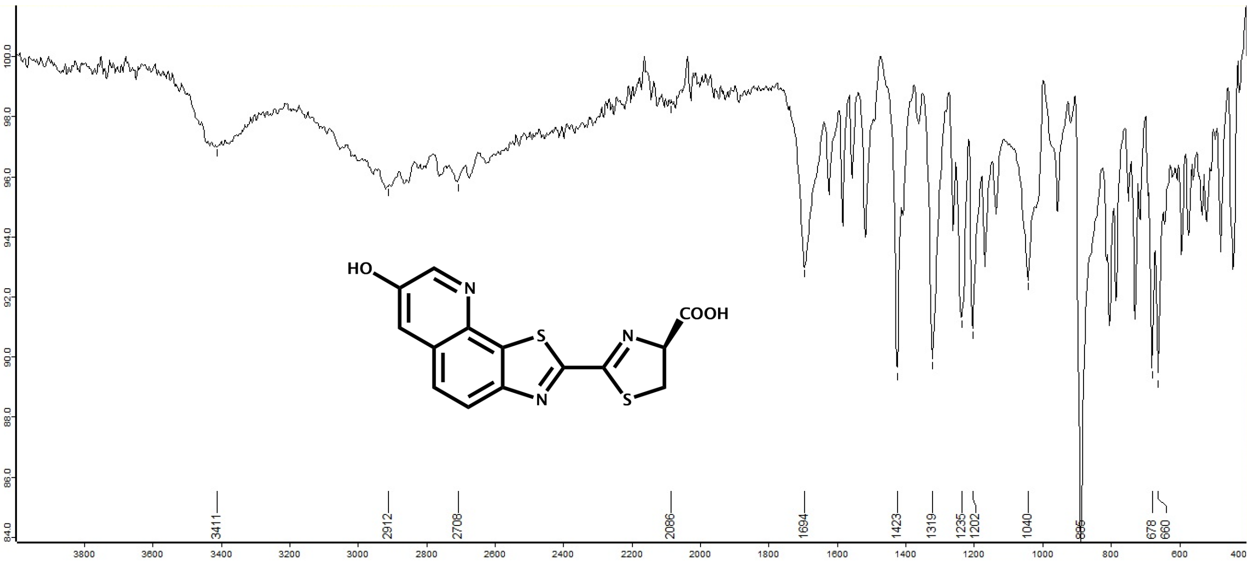

Figure S6. IR spectra of NH_2-QLH_2 and OH-QLH_2 and the synthetic intermediates compounds leading to the final products. (A) **1**; (B) **2**; (C) **3**; (D) **4**; (E) **5**; (F) **7**; (G) **8**; (H) **9**; (I) **10**; (J) **11**; (K) **12**; (L) **13** (numbers in bold correspond to compound numbers in synthesis section above).

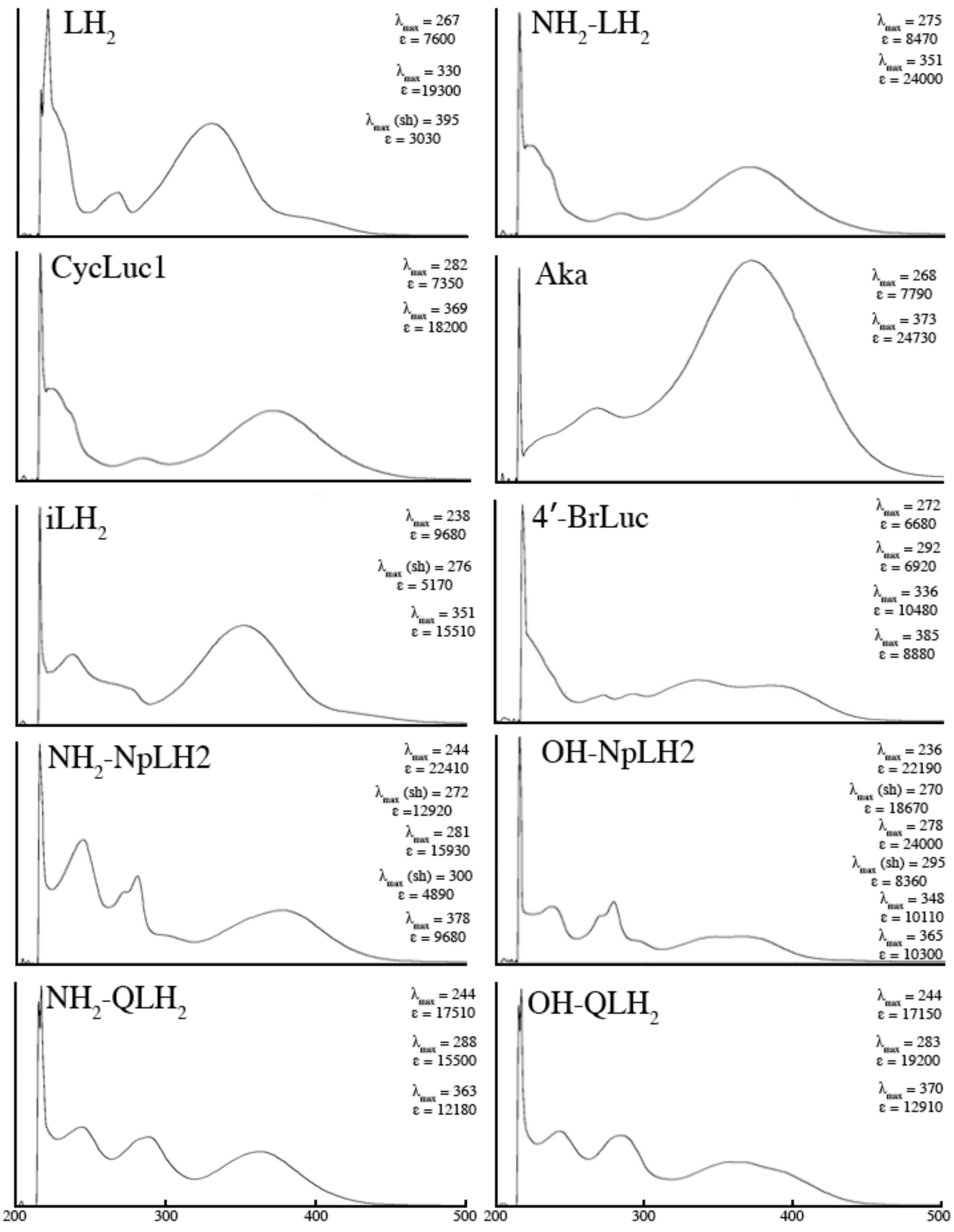

Figure S7. UV/Vis spectra of the substrates in Figure 1 acquired in 50 mM tricine buffer, pH 7.4. The y-axis represents absorbance and the x-axis represents wavelength (nm). λ_{max} values are in nm. ε values are in $M^{-1}cm^{-1}$.

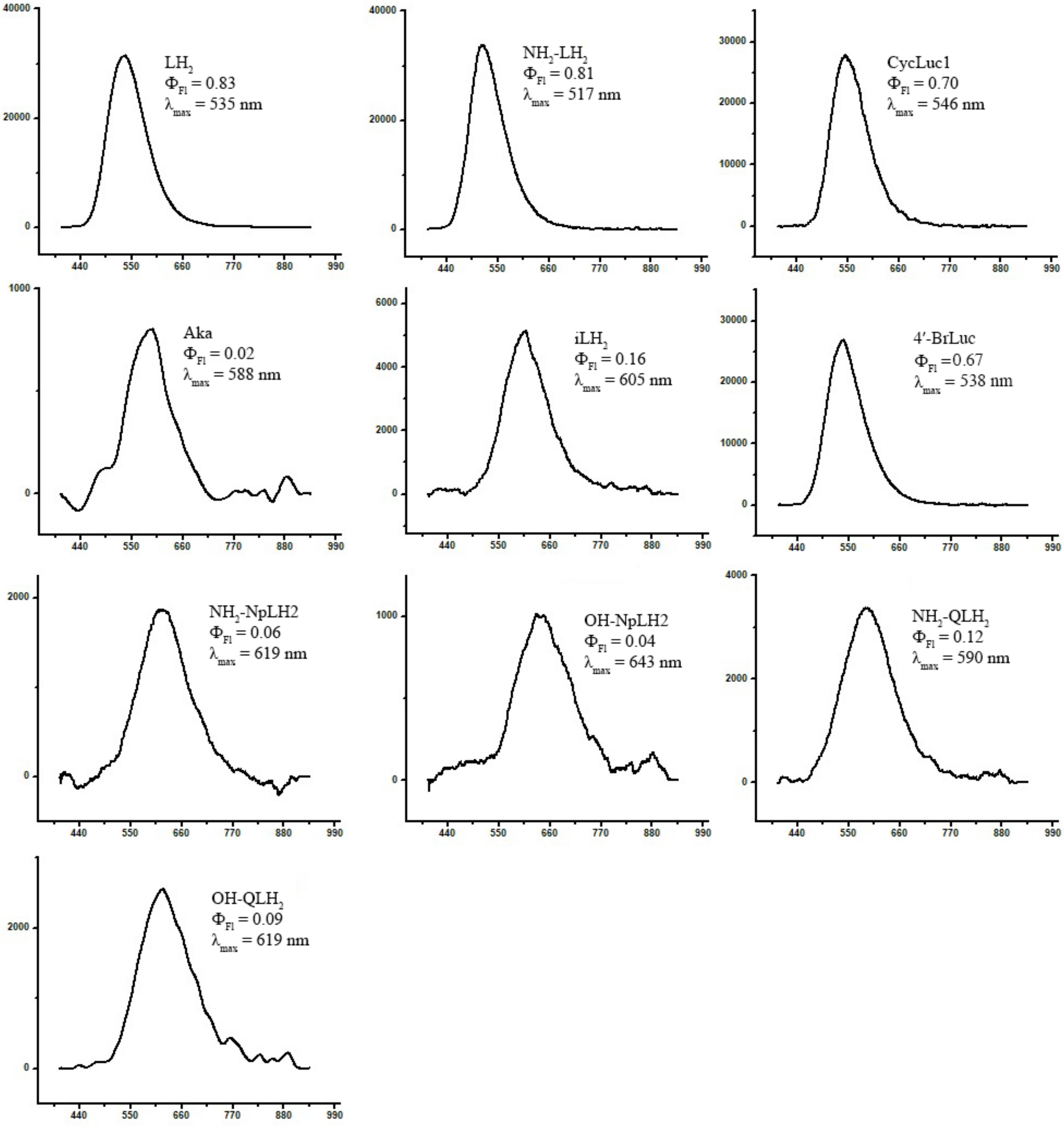

Figure S8. Fluorescence emission spectra and quantum yields of the substrates in Figure 1. Measurements were made in solutions with absorbance of 0.10 Au at 370 nm in 50 mM $NaHCO_3$, pH 11. Excitations were performed at 370 nm. The y-axis represents intensity (counts) and the x-axis represents wavelength (nm).

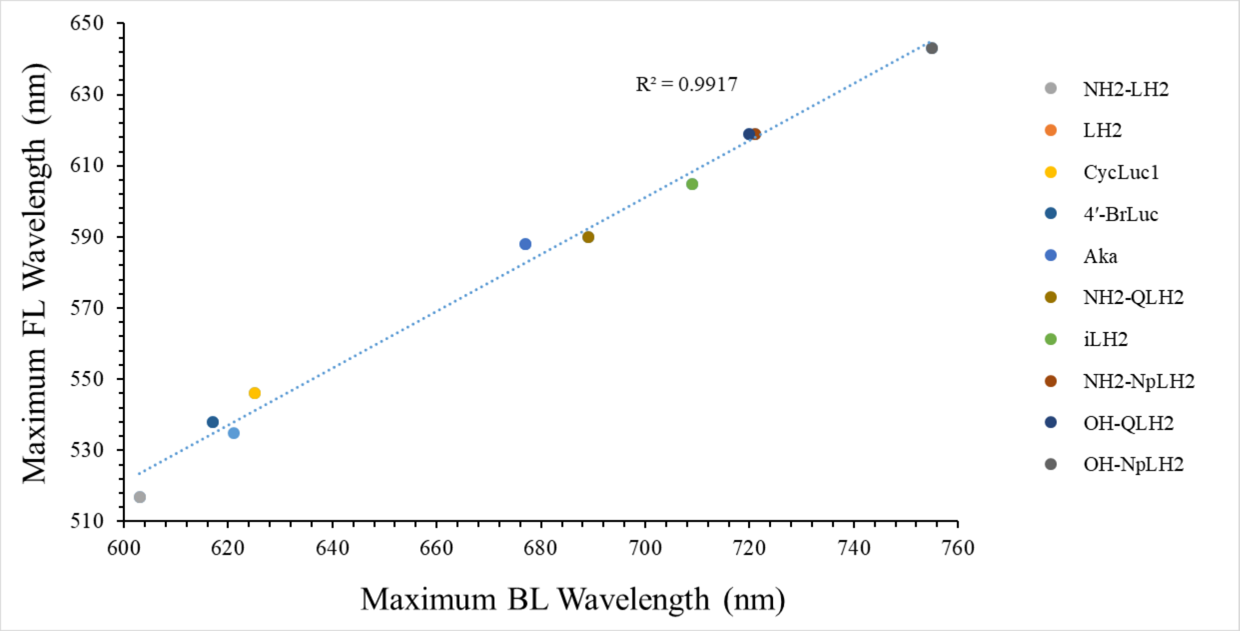

Figure S9. Comparison the maximum BL wavelength of the substrates in Figure 1 that could be achieved with the 7 enzymes used in the study and the FL maximum wavelength.

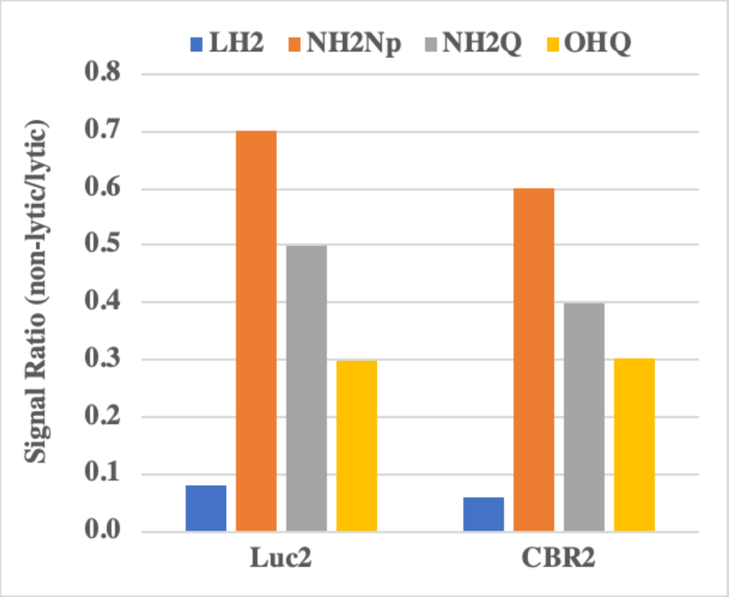

Figure S10. Membrane permeability of LH_2 and naphthyl- and quinolinyl-containing analogs with Luc2 and CBR2 transiently expressed in HEK293T cells. Data represents the ratio of BL measured as average radiance (p/s/cm²/sr) produced from non-lytic *vs* lytic assay conditions.

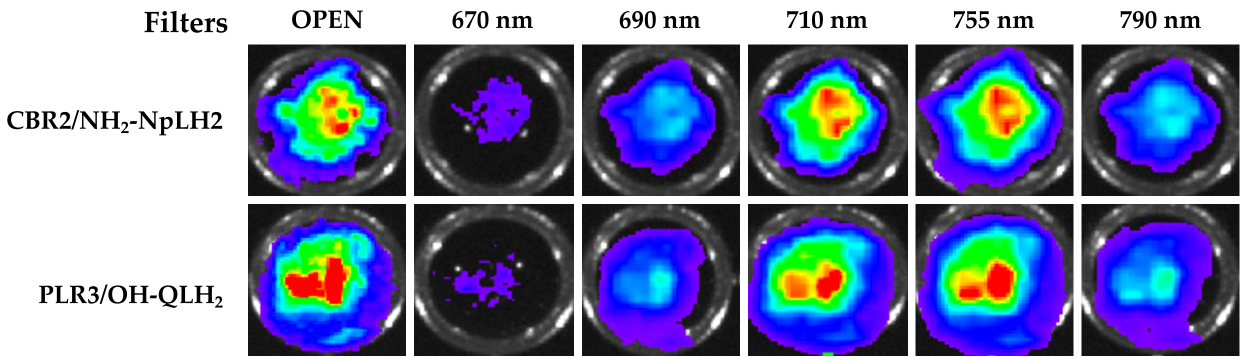

Figure S11. Comparison of BL emission intensity and wavelength distribution of CBR2/NH_2-NpLH2 and PLR3/OH-QLH_2 in HEK293T cells. BL images of live cells in a 96-well plate were recorded through filters using the IVIS Spectrum III instrument and displayed using Living Image 4.7.2 software. Cells were transfected, incubated with substrate and imaged as described in Materials and Methods.

VIII. TABLES: CHARACTERIZATION OF SUBSTRATES

Table S1. BL emission and relative activities of all Luc-substrate pairs.

LUC/SUBSTRATE	LH_2	NH_2-LH2	CycLuc1	Aka	iLH_2	4´-BrLuc	NH_2-NpLH2	OH-NpLH2	NH_2-QLH_2	OH-QLH_2
**Luc2**	100 ± 4	9.1 ± 0.48	8.3 ± 0.19	1.9 ± 0.01	0.07 ± 0.001	8.3 ± 0.78	0.18 ± 0.003	b.d.	0.08 ± 0.001	0.03 ± 0.003
Live Cell	100 ± 9.7	36 ± 3.9	47 ± 4.4	10 ± 1.0	0.06 ± 0.007	4.8 ± 0.3	0.58 ± 0.06	b.d.	0.18 ± 0.015	0.03 ± 0.003
Biolum λmax (nm), 23 °C	562 (84)	603 (85)	609 (61)	677 (90)	709 (96)	617 (70)	708 (135)	b.d.	678 (139)	720 (154)
Biolum λmax (nm), 37 °C	600,574 (105)	602 (80)	606 (64)	674 (88)	708 (99)	618 (70)	706 (126)	b.d.	678 (130)	719 (133)
Live Cell optimal filter	570	620	620	670	710	620	710	b.d.	670	710
**RedFluc**	50.7 ± 1.5	27.40 ± 0.63	14.25 ± 3.3	2.6 ± 0.12	0.17 ± 0.01	37 ± 0.48	0.91 ± 0.01	b.d	1.10 ± 0.01	1.93 ± 0.11
Live Cell	27 ± 2.7	43 ± 2.1	48 ± 3.2	15 ± 1.5	0.05 ± .01	7.5 ± 1.5	0.45 ± 0.04	b.d.	0.17 ± 0.01	0.41 ± 0.02
Biolum λmax (nm), 23 °C	612 (60)	595 (73)	605 (68)	671 (77)	718 (95)	616(66)	694 (112)	b.d	660 (117)	715 (107)
Biolum λmax (nm), 37 °C	613 (63)	594 (76)	604 (69)	670 (83)	720 (85)	616 (75)	694 (116)	b.d	659 (122)	715 (107)
Live Cell optimal filter	620	620	620	670	710	620	670	b.d	670	710
**Akaluc**	0.10 ± 0.01	0.84 ± 0.02	4.17 ± 0.21	1.62 ± 0.03	b.d.	b.d.	b.d.	b.d.	b.d.	b.d.
Live Cell	4.2 ± 0.63	8.5 ± 0.8	114 ± 22	152 ± 19	0.04 ± 0.001	0.04 ± 0.002	0.05 ± 0.004	b.d.	b.d.	b.d.
Biolum λmax (nm), 23 °C	598 (73)	588 (75)	595 (70)	642 (93)	682 (95)	613 (81)	682 (116)	b.d	b.d	b.d
Biolum λmax (nm), 37 °C	597 (73)	587 (78)	593 (70)	643 (89)	681 (104)	614 (82)	685 (131)	b.d	b.d	b.d
Live Cell optimal filter	620	570	620	670	670	n.d.	670	b.d	b.d	b.d.
**Mut51**	1.87 ± 0.02	1.77 ± 0.07	4.85 ± 0.22	0.61 ± 0.05	0.03 ± 0.001	1.9 ± 0.08	0.06 ± 0.004	b.d.	b.d.	b.d.
Live Cell	0.94 ± 0.06	2.4 ± 0.21	20 ± 1.3	1.2 ± 0.1	b.d.	2.3 ± 0.7	0.16 ± 0.02	b.d.	b.d.	b.d.
Biolum λmax (nm), 23 °C	568 (101)	598 (78)	608 (69)	672 (84)	708 (109)	617 (77)	701 (118)	b.d	665 (122)	b.d
Biolum λmax (nm), 37 °C	612 (86)	594 (79)	608 (69)	670 (86)	714 (102)	618 (76)	700 (124)	b.d	668 (115)	b.d
Live Cell optimal filter	620	620	620	670	b.d.	620	710	b.d	620	620
**Fluc_Red**	47.11 ± 2.6	11.52 ± 0.32	4.13 ± 0.18	1.04 ± 0.02	0.14 ± 0.02	9.5 ± 0.2	0.14 ± 0.001	b.d.	0.04 ± 0.003	0.17 ± 0.005
Live Cell	272 ± 28	119 ± 12	111 ± 11	109 ± 12	0.56 ± 0.05	29 ± 1.5	2.2 ± 0.25	0.04 ± 0.002	0.28 ± 0.02	0.93 ± 0.07
Biolum λmax (nm), 23 °C	602 (76)	595 (80)	603 (60)	662 (85)	707 (93)	616 (73)	690 (125)	727 (122)	660 (141)	701 (121)
Biolum λmax (nm), 37 °C	604 (68)	593 (76)	603 (65)	661 (88)	707 (86)	616 (74)	693 (125)	733 (136)	665 (117)	703 (123)
Live Cell optimal filter	620	570	620	670	710	620	670	b.d.	670	710
**CBR2**	52.1 ± 0.26	10.1 ± 0.24	2.69 ± 0.26	0.62 ± 0.03	0.14 ± 0.002	5.4 ± 0.37	0.42 ± 0.01	0.48 ± 0.01	0.25 ± 0.001	0.89 ± 0.01
Live Cell	158 ± 15	168 ± 31	25 ± 0.2	10 ± 0.05	1.3 ± 0.04	25 ± 3.9	3.2 ± 0.09	2.2 ± 0.13	0.76 ± 0.02	1.3 ± 0.11
Biolum λmax (nm), 23 °C	621 (65)	598 (71)	625 (62)	686 (77)	730 (89)	616 (89)	721 (120)	750 (117)	689 (123)	719 (126)
Biolum λmax (nm), 37 °C	619 (64)	600 (72)	622 (66)	575,684 (76)	727 (87)	619 (73)	718 (117)	749 (116)	688 (123)	721 (118)
Live Cell optimal filter	620	620	620	670	710	620	710	755	670	710
**PLR3**	74.3 ± 8	13.7 ± 0.95	13.2 ± 0.24	0.82 ± 0.08	0.21 ± 0.01	44 ± 2	0.27 ± 0.001	0.004 ± 0.0001	1.23 ± 0.003	2.82 ± 0.06
Live Cell	148 ± 15	118 ± 6.5	257 ± 12	16 ± 1.1	0.32 ± 0.03	60 ± 8.7	2.3 ± 0.13	b.d.	2.9 ± 0.14	4.1 ± 0.35
Biolum λmax (nm), 23 °C	613 (61)	593 (72)	606 (62)	667 (88)	723 (109)	615 (63)	693 (110)	755 (104)	649 (111)	718 (107)
Biolum λmax (nm), 37 °C	611 (62)	593 (76)	602 (65)	665 (91)	718 (108)	615 (69)	691 (103)	751 (107)	649 (113)	716 (107)
Live Cell optimal filter	620	570	620	670	710	620	670	b.d.	670	710

BL activities, reported on the top line for each enzyme entry/substrate entry were obtained with purified Lucs and are reported relative to the Luc2-LH_2 value defined as 100. Live cell BL activities are reported relative to the Luc2-LH_2 value defined as 100 and were determined from the mean radiance (p/s/cm²/sr) values. The detailed protocol can be found in the Materials and Methods of the main text. BL emission spectra measured in *in vitro* assays were obtained as described in Materials and Methods of main text. Bandwidths at full width at half-maximum values are shown in parentheses. Live cell optimal filter data is reported as the highest BL activity obtained from the following filter range: 520 ± 20 nm, 570 ± 20 nm, 620 ± 20 nm, 670 ± 20 nm, 710 ± 20 nm, 755 ± 15 nm and 790 ± 20 nm. b.d; below detection having a relative specific activity of < 0.03.

Table S2. Calculated logP (ACD NMR) and C_{18} retention time (method described in the materials and methods) of the substrates in Figure 1.

Substrate	Log*P*	Retention Time (min)
LH_2	0.87 ± 1.05	5.54
NH_2-LH_2	0.32 ± 1.05	1.58
CycLuc1	1.01 ± 1.45	2.37
Aka	2.06 ± 0.78	4.13
iLH_2	1.33 ± 1.08	5.85
4′-BrLuc	2.01 ± 1.08	14.69
NH_2-NpLH2	1.55 ± 1.05	5.75
OH-NpLH2	2.10 ± 1.05	
NH_2-QLH_2	0.88 ± 1.27	8.56
OH-QLH_2	1.46 ± 1.33	11.47

IX. REFERENCES

1. Appel, R., Janssen, H., Siray, M., and Knoch, F. (1985) Synthese und Reaktionnen des 4,5-Dichlor-1,2,3-dithiazolium-chlorids, *Chem. Ber. 118*, 1632-1643.
2. McCutcheon, D. C., Porterfield, W. B., and Prescher, J. A. (2015) Rapid and scalable assembly of firefly luciferase substrates, *Organic & Biomolecular Chemistry 13*, 2117-2121.
3. Hall, M. P., Woodroofe, C. C., Wood, M. G., Que, I., Van'T Root, M., Ridwan, Y., Shi, C., Kirkland, T. A., Encell, L. P., Wood, K. V., Löwik, C., and Mezzanotte, L. (2018) Click beetle luciferase mutant and near infrared naphthyl-luciferins for improved bioluminescence imaging, *Nat Commun 9*.
4. Branchini, B. R., Ablamsky, D. M., Davis, A. L., Southworth, T. L., Butler, B., Fan, F., Jathoul, A. P., and Pule, M. A. (2010) Red-emitting luciferases for bioluminescence reporter and imaging applications, *Anal. Biochem. 396*, 290-297.
5. Branchini, B. R., Southworth, T. L., Fontaine, D. M., Davis, A. L., Behney, C. E., and Murtiashaw, M. H. (2014) A Photinus pyralis and Luciola italica Chimeric Firefly Luciferase Produces Enhanced Bioluminescence, *Biochemistry 53*, 6287-6289.
6. Branchini, B. R., Southworth, T. L., Murtiashaw, M. H., Wilkinson, S. R., Khattak, N. F., Rosenberg, J. C., and Zimmer, M. (2005) Mutagenesis evidence that the partial reactions of firefly bioluminescence are catalyzed by different conformations of the luciferase C-terminal domain, *Biochemistry 44*, 1385-1393.
7. Branchini, B. R., Southworth, T. L., Fontaine, D. M., Murtiashaw, M. H., McGurk, A., Talukder, M. H., Qureshi, R., Yetil, D., Sundlov, J. A., and Gulick, A. M. (2017) Cloning of the Orange Light-Producing Luciferase from Photinus scintillansA New Proposal on how Bioluminescence Color is Determined, *Photochem. Photobiol. 93*, 479-485.
8. Mofford, D. M., Reddy, G. R., and Miller, S. C. (2014) Aminoluciferins Extend Firefly Luciferase Bioluminescence into the Near-Infrared and Can Be Preferred Substrates over D-Luciferin, *J. Am. Chem. Soc. 136*, 13277-13282.
